# Synthesis of Benzothiophene-3-carboxylic Esters by Palladium Iodide-Catalyzed Oxidative Cyclization–Deprotection–Alkoxycarbonylation Sequence under Aerobic Conditions

**DOI:** 10.1021/acs.joc.2c00686

**Published:** 2022-05-10

**Authors:** Raffaella Mancuso, Simona Cuglietta, Romina Strangis, Bartolo Gabriele

**Affiliations:** Laboratory of Industrial and Synthetic Organic Chemistry (LISOC), Department of Chemistry and Chemical Technologies, University of Calabria, Via Pietro Bucci 12/C, 87036 Arcavacata di Rende (CS), Italy

## Abstract

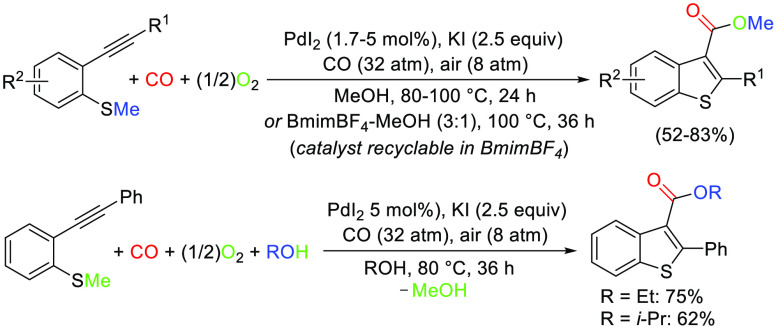

A palladium-catalyzed carbonylative approach to benzothiophene-3-carboxylic esters, starting from simple and readily available building blocks [2-(methylthio)phenylacetylenes, CO, an alcohol, and O_2_ (from air)], is reported. The process is catalyzed by the simple PdI_2_/KI catalytic system to give the desired products in fair to high yields (57–83%). Interestingly, the reaction also works nicely in the ionic liquid BmimBF_4_ as the solvent, with the possibility to recycle the catalytic system several times without appreciable loss of activity.

The use of carbon monoxide as a C-1 building block for the direct synthesis of carbonylated heterocycles represents a major tool in current organic synthesis.^1^ Among carbonylation processes, palladium(II)-catalyzed oxidative heterocyclization–alkoxycarbonylation reactions constitute a powerful methodology for the preparation of a variety of important heterocyclic derivatives starting from simple and readily available acyclic substrates.^2^

In this field, in the course of the recent years, our group has contributed a number of examples, based on the successful use of a particularly simple catalytic system, consisting of palladium iodide in conjunction with an excess of potassium iodide (“PdI_2_/KI catalyst”).^3^ A main characteristic of our system, besides avoiding the use of additional ligands or promoters apart from KI, is that it works very well with the simplest and most convenient oxidant possible, that is, oxygen (from air), without any need for other inorganic or organic oxidants (such as copper chloride or benzoquinone).^3^

A possible limitation on the use of the PdI_2_/KI catalytic system in an oxidative S-heterocyclization–carbonylation process can be related to the well-known instability of sulfur-based nucleophiles (such as thiols) under aerobic conditions. Very recently, however, we have shown that it is possible to overcome this limitation by suitably “masking” the thiol group of the acyclic substrate by simple methylation. In fact, we found that 1-(methylthio)-3-yn-2-ols could be conveniently converted into thiophene-3-carboxylic esters by a sequence of steps involving 5-endo-dig S-cyclization, iodide-promoted demethylation, dehydrative alkoxycarbonylation, and Pd(0) aerobic reoxidation.^4^

In this Note, we report a useful extension of this kind of reactivity to the use of readily available 2-(methylthio)phenylacetylenes, which allows synthesizing high-value-added benzothiophene-3-carboxylic esters^5^ in a multicomponent fashion and using O_2_ (from air) as the sole benign external oxidant.^6^

On the basis of our previous results on PdI_2_/KI-catalyzed oxidative carbonylation of 1-(methylthio)-3-yn-2-ols to give thiophene-3-carboxylic esters,^4^ in this work we have assessed the reactivity of 2-(methylthio)phenylacetylenes **1** (readily available by Sonogashira coupling between 2-halothioanisoles and terminal alkynes, as described in the Supporting Information) under similar conditions, with the aim of synthesizing benzothiophene-3-carboxylic esters **2**. According to our mechanistic hypothesis, the formation of the desired products **2** can occur through an ordered sequence in steps involving the following: (a) triple bond coordination to the metal center; (b) 5-endo-dig S-cyclization, by intramolecular nucleophilic attack of the *o*-methythio group on the coordinated triple bond, to give sulfonium iodide intermediate **I**; (c) demethylation of **I** by the iodide anion, with formation of MeI and intermediate **II**; (d) reaction of MeI with water (initially present in the reaction mixture as an impurity and then also formed in the Pd(0) reoxidation step) with formation of MeOH and HI; (e) carbon monoxide insertion into the palladium–carbon bond of **II** to give **III**; (f) nucleophilic displacement by attack of an external alcohol ROH (either MeOH or a higher alcohol, used as the solvent) on **III**, to yield the benzothiophene-3-carboxylic ester **2**, another 1 mol of HI, and Pd(0); (g) Pd(0) reoxidation to PdI_2_, through oxidation of 2 mol of HI by oxygen, to give water and I_2_, followed by oxidative addition of the latter to Pd(0) (Scheme 1; anionic iodide ligands are not shown for clarity).

**Scheme 1:**
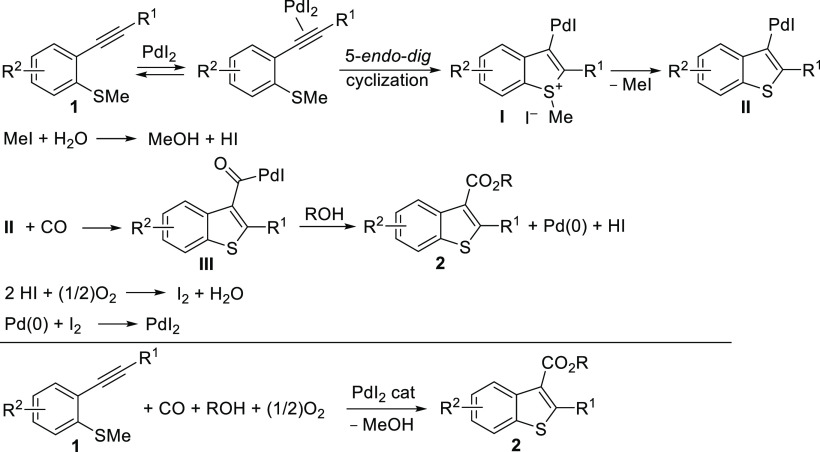
Mechanistic Hypothesis Leading to Benzothiophene-3-carboxylic Esters **2** by PdI_2_-Catalyzed Oxidative Carbonylation of 2-(Methylthio)phenylacetylenes **1** and Stoichiometry of the Process Anionic iodide ligands are omitted for clarity.

To verify our work hypothesis, we first synthesized methyl(2-(phenylethynyl)phenyl)sulfane **1a** (R = Ph) and used it as model substrate to optimize reaction conditions. The first reaction was carried out under conditions similar to those already successfully employed for the synthesis of thiophene-3-carboxylates,^4^ that are 5 mol % of PdI_2_ in the presence of an excess of KI (5 equiv) in MeOH as the solvent and external nucleophile (R = Me) (substrate initial concentration, 0.02 mmol/mL of MeOH) at 80 °C and under 40 atm of a 4:1 mixture of CO–air. After 15 h reaction time, substrate conversion (determined by isolation of unreacted **1a**) was 91%, with formation of two carbonylation products: the desired methyl 2-phenylbenzo[*b*]thiophene-3-carboxylate **2a** (43% isolated yield) and maleic diester **3a** (7% GLC yield) derived from triple bond oxidative dialkoxycarbonylation^7^ (Table S1, entry 1, Supporting Information).

To improve this initial result and make the process more selective toward **2a**, we then carried out several experiments by changing the reaction conditions; the results obtained are shown and detailed in the Supporting Information (Table S1 and related text). Based on this brief reaction optimization study (see the Supporting Information), the best conditions for the selective formation of benzothiophene-3-carboxylate **2a** are those of Table S1, entry 3. To further improve this product yield and achieve total substrate conversion, we then allowed the reaction to run under those conditions for 24 h instead of 15 h: gratifyingly, **2a** could be isolated in 80% yield (Table 1, entry 1).^8^ The process could also be carried out with lower catalyst loadings, as shown by the results reported in Table 1, entries 2 and 3 (a yield of ca. 60% of **2a** was obtained with both 2.5 mol % and 1.7 mol % of PdI_2_). Changing the reaction solvent and external nucleophile from MeOH to EtOH (R = Et) or *i*-PrOH (R = *i*-Pr) caused a slowdown of the reaction rate, so the process was carried out for 36 h rather than 24 h. The corresponding ethyl and isopropyl esters, however (**2a′** and **2a′′**, respectively), could be isolated in satisfactory yields (75% and 62%, respectively; Table 1, entries 4 and 5).

**Table 1:**
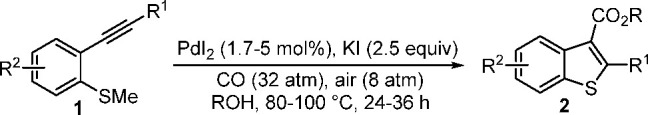

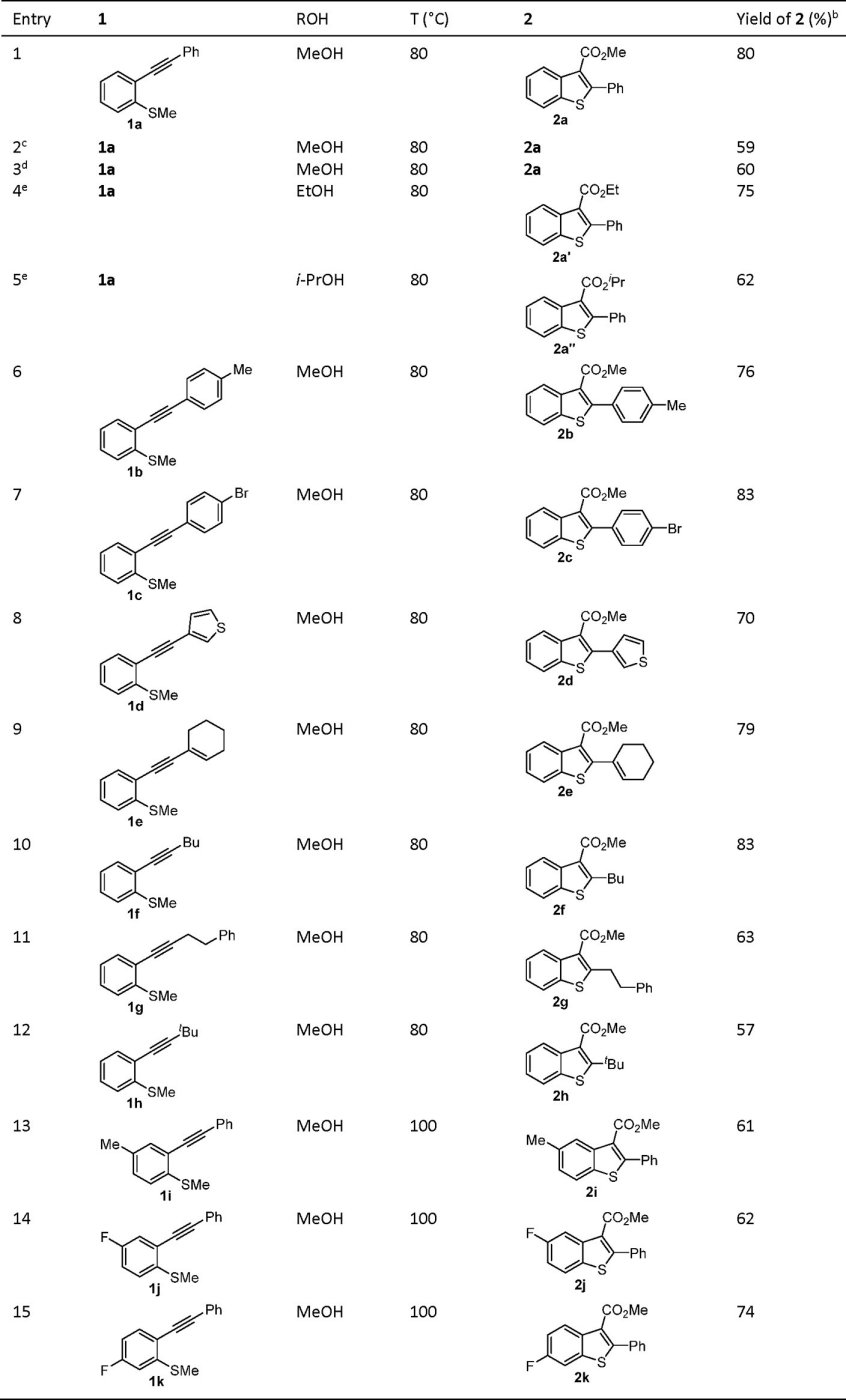
Synthesis of Benzo[*b*]thiophene-3-carboxylic Esters **2** by PdI_2_/KI-Catalyzed Alkoxycarbonylation of 2-(Methylthio)phenylacetylenes **1**^a^

a Unless otherwise noted, all reactions were carried out in ROH as the solvent (0.02 mmol of **1** per mL of ROH), in the presence of PdI_2_ (5 mol %) and KI (2.5 equiv) for 24 h, under 40 atm of a 4:1 mixture of CO–air. Substrate conversion was quantitative in all cases.

b Isolated yield based on starting **1**.

c The reaction was carried out with a KI:**1a**:PdI_2_ molar ratio of 100:40:1.

d The reaction was carried out with a KI:**1a**:PdI_2_ molar ratio of 150:60:1.

e The reaction was carried out for 36 h.

The method was then extended to other 2-(methylthio)phenylacetylenes, **1b**–**k**, bearing different substituents R^1^ on the triple bond and R^2^ on the aromatic ring. As can be seen from the results reported in Table 1, entries 6 and 7, high yields of the corresponding benzothiophene-3-carboxylates were obtained when R^1^ was an aryl group para-substituted with either an electron-releasing group (such as a methyl; yield of **2b**, 76%) or an electron-withdrawing halogen atom (such as bromine; yield of **2c**, 83%). A heteroaryl substituent such as 3-thienyl was also compatible with reaction conditions, with formation of methyl 2-(thiophen-3-yl)benzo[*b*]thiophene-3-carboxylate **2d** in 70% yield (Table 1, entry 8), as well as an alkenyl group, such as 1-cyclohexenyl (yield of **2e**, 79%, Table 1, entry 9). The process also worked nicely when R^1^ was a simple alkyl group (such as butyl; yield of **2f**, 83%, Table 1, entry 10), phenethyl (yield of **2g**, 63%; Table 1, entry 11) and even a sterically demanding group, such as *tert*-butyl, with an acceptable yield of methyl 2-(*tert*-butyl)benzo[*b*]thiophene-3-carboxylate **2h** of 57% (Table 1, entry 12). Good yields of the corresponding benzothiophene-3-carboxylic esters **2i**–**k** (61–74%) were also achieved when the aromatic ring of the substrate bore either an electron-donating (such as Me, substrate **1i**; Table 1, entry 13) or an electron-withdrawing group (such as F, substrates **1j** and **1k**; Table 1, entries 14 and 15).

To test the possibility to recycle the catalyst, we also performed the reaction of the parent substrate **1a** in an ionic liquid as the solvent, such as 1-butyl-3-methylimidazolium tetrafluoroborate (BmimBF_4_) in which the PdI_2_/KI system is perfectly soluble.^9^ The methoxycarbonylation of **1a**, carried out in a 3:1 mixture of BmimBF_4_-MeOH under the same conditions already optimized in MeOH, led to the formation of benzothiophene **2a** in 40% yield at 75% substrate conversion. Complete conversion of **1a**, with a good yield of **2a** (68%), could be obtained working at 100 °C under more concentrated conditions (0.05 rather than 0.02 mmol of **1a** per mL of solvent) for 36 h (Table S2, entry 1, run 1, Supporting Information). Recycling experiments showed that no appreciable loss of catalytic activity occurred even after the fifth recycle (Table S2, entry 1, runs 2–6). Similar results were obtained with the other substrates tested, **1b**, **1d**, and **1g**, as shown in Table S2, entries 2–4.

In conclusion, we have found that PdI_2_ in conjunction with an excess of KI is able to catalyze the oxidative alkoxycarbonylation of readily available 2-(methylthio)phenylacetylenes under aerobic conditions (with O_2_ from air as the external oxidant) to give high-value-added benzothiophene-3-carboxyxlic esters in a selective manner. The process takes place by intramolecular S-5-endo-dig cyclization followed by iodide-promoted S-demethylation, alkoxycarbonylation, and Pd(0) reoxidation to close the catalytic cycle. The catalytic system can be also conveniently recycled without appreciable loss of activity working in BmimBF_4_ as the solvent. Our method thus provides a convenient direct catalytic approach to an important class of heterocyclic derivatives in one step and in a multicomponent fashion, starting from very simple building blocks (the acyclic organic substrate, CO, an alcohol, and O_2_).

## Experimental Section

### General Experimental Methods

Solvent and chemicals were reagent grade and were used without further purification. All reactions were analyzed by TLC on silica gel 60 F254 and by GLC using capillary columns with polymethylsilicone + 5% phenylsilicone as the stationary phase. Column chromatography was performed on silica gel 60 (70–230 mesh). Evaporation refers to the removal of solvent under reduced pressure. Melting points are uncorrected. ^1^H NMR and ^13^C{^1^H}NMR spectra were recorded at 25 °C on a 300 or 500 MHz spectrometer in CDCl_3_ as the solvent and with Me_4_Si as internal standard. Chemical shifts (δ) and coupling constants (*J*) are given in ppm and in hertz (Hz), respectively. IR spectra were taken with an FT-IR spectrometer. Mass spectra were obtained using a GC-MS apparatus at 70 eV ionization voltage (normal resolution) and by electrospray ionization mass spectrometry (ESI-MS) (high resolution) with a UHD accurate-mass Q-TOF spectrometer equipped with a Dual AJS ESI source working in positive mode and were recorded in the 150–1000 *m*/*z* range. The LC-MS experimental conditions were as follows: N_2_ was employed as desolvation gas at 300 °C and a flow rate of 9 L/min. The nebulizer was set to 45 psig. The Sheat gas temperature was set at 350 °C and a flow of 12 L/min. A potential of 3.5 kV was used on the capillary for positive ion mode. The fragmentor was set to 175 V.

### Preparation of Substrates

Methyl(2-alkynyl)phenyl)sulfanes **1a**–**k** were prepared by Sonogashira coupling between commercially available 2-halothioanisoles and terminal alkynes as described in the Supporting Information.

### General Procedure for the Synthesis of Benzo[*b*]thiophen-3-carboxylic Esters **2a**–**k**, **2a′**, and **2a′′** (Table 1)

A 250 mL stainless-steel autoclave was charged in the presence of air with PdI_2_ (5.4 mg, 0.015 mmol), KI (125 mg, 0.75 mmol), and substrate **1** (0.30 mmol; **1a**, 67.5 mg; **1b**, 71.6 mg; **1c**, 91.0 mg; **1d**, 69.3 mg; **1e**, 68.6 mg; **1f**, 61.5 mg; **1g**, 75.8 mg; **1h**, 61.3 mg; **1i**, 71.5 mg; **1j**, 72.6 mg; **1k**, 72.7 mg) in ROH (15 mL). The autoclave was sealed and, while the mixture was stirred, the autoclave was pressurized with CO (32 atm) and air (up to 40 atm). After being stirred at 80 °C (substrates **1a**–**h**) or 100 °C (substrates **1i**–**k**) (jacketed autoclave with circulating thermic fluid) for 24 h (ROH = MeOH) or 36 h (ROH = EtOH, *i*-PrOH), the autoclave was cooled, degassed, and opened. The solvent was evaporated, and the products were purified by column chromatography on silica gel using as eluent hexane–AcOEt from 100:0 to 95:5 (for **2a**-**h**, **2a′** and **2a′′**) or hexane–Et_2_O from 100:0 to 99.5%–0.5% (for **2i**–**k**).

#### Methyl 2-Phenylbenzo[*b*]thiophene-3-carboxylate (**2a**)

Purified by column chromatography on silica gel using as eluent hexane to 95:5 hexane–AcOEt; yield: 64.6 mg, starting from 67.5 mg of methyl(2-(phenylethynyl)phenyl)sulfane **1a** (80%; Table 1, entry 1). Yellow solid, mp = 54–55 °C. IR (KBr): ν = 1716 (s), 1458 (w), 1433 (m), 1356 (w), 1230 (m), 1207 (m), 752 (m), 695 (w) cm^–1^; ^1^H NMR (CDCl_3_, 300 MHz): δ = 8.35 (d, *J* = 8.0, 1H), 7.81 (d, *J* = 8.0, 1H), 7.55–7.32 (m, 7H), 3.76 (s, 3H); ^13^C{^1^H}NMR (CDCl_3_, 75 MHz): δ = 164.5, 151.9, 138.6, 138.5, 134.0, 129.4, 128.9, 128.2, 125.4, 125.0, 124.6, 122.9, 121.7, 51.6; GC-MS (EI, 70 eV): *m*/*z* = 268 (M^+^, 87), 237 (100), 208 (25), 165 (33), 104 (15); HRMS-ESI (*m*/*z*): [(M + H)^+^] cald for (C_16_H_13_O_2_S)^+^: 269.0631; found: 269.0624. The spectroscopic data were in good agreement with those reported.^10^

#### Ethyl 2-Phenylbenzo[*b*]thiophene-3-carboxylate (**2a′**)

Purified by column chromatography on silica gel using as eluent hexane to 95:5 hexane–AcOEt; yield: 63.7 mg, starting from 67.3 mg of methyl(2-(phenylethynyl)phenyl)sulfane **1a** (75%; Table 1, entry 4). Yellow oil. IR (film): ν = 1709 (s), 1459 (m), 1433 (w), 1373 (m), 1345 (m), 1278 (w), 1230 (m), 1203 (m), 752 (m) cm^–1^; ^1^H NMR (CDCl_3_, 500 MHz): δ = 8.35 (d, *J* = 8.1, 1H), 7.81 (d, *J* = 8.1, 1H), 7.53–7.36 (m, 7H), 4.23 (q, *J* = 7.1, 2H), 1.12 (t, *J* = 7.1, 3H); ^13^C{^1^H}NMR (CDCl_3_, 125 MHz): δ = 164.0, 151.6, 138.59, 138.55, 134.2, 130.0, 128.8, 128.0, 125.4, 124.9, 124.6, 123.3, 121.7, 60.6, 13.8; GC-MS (EI, 70 eV): *m*/*z* = 282 (M^+^, 100), 254 (16), 237 (98), 208 (27), 165 (39), 104 (12); HRMS-ESI (*m*/*z*): [(M + H)^+^] cald for (C_17_H_15_O_2_S)^+^: 283.0787; found: 283.0788.

#### Isopropyl 2-Phenylbenzo[*b*]thiophene-3-carboxylate (**2a”**)

Purified by column chromatography on silica gel using as eluent hexane to 95:5 hexane–AcOEt; yield: 55.3 mg, starting from 67.6 mg of methyl(2-(phenylethynyl)phenyl)sulfane **1a** (62%; Table 1, entry 5). Yellow oil. IR (film): ν = 1701 (s), 1458 (m), 1369 (m), 1277 (w), 1207 (m), 1107 (m), 725 (m) cm^–1^; ^1^H NMR (CDCl_3_, 500 MHz): δ = 8.37 (d, *J* = 8.1, 1 H), 7.81 (d, *J* = 8.1, 1H), 7.53–7.36 (m, 7H), 5.15 (heptuplet, *J* = 6.3, 1 H), 1.13 (d, *J* = 6.3, 6 H); ^13^C{^1^H}NMR (CDCl_3_, 125 MHz): δ = 163.5, 151.2, 138.63, 138.61, 134.3, 129.6, 128.7, 128.0, 125.4, 124.9, 124.6, 121.7, 68.3, 21.6; GC-MS (EI, 70 eV): *m*/*z* = 296 (M^+^, 83), 254 (100), 237 (68), 208 (26), 165 (41); HRMS-ESI (*m*/*z*): [(M + H)^+^] cald for (C_18_H_17_O_2_S)^+^: 297.0944; found: 297.0943.

#### Methyl 2-(*p*-Tolyl)benzo[*b*]thiophene-3-carboxylate (**2b**)

Purified by column chromatography on silica gel using as eluent hexane to 95:5 hexane–AcOEt; yield: 64.6 mg, starting from 71.6 mg of methyl(2-(*p*-tolylethynyl)phenyl)sulfane **1b** (76%; Table 1, entry 6). Yellow solid, mp = 41–43 °C. IR (KBr): ν = 1713 (s), 1458 (w), 1431 (m), 1354 (m), 1277 (w), 1215 (s), 814 (w), 760 (w) cm^–1^; ^1^H NMR (CDCl_3_, 300 MHz): δ = 8.31 (d, *J* = 8.0, 1H), 7.81 (d, *J* = 8.0, 1H), 7.51–7.32 (m, 4H), 7.29–7.17 (m, 2H, aromatic), 3.79 (s, 3H), 2.42 (s, 3H); ^13^C{^1^H}NMR (CDCl_3_, 75 MHz): δ = 164.6, 152.1, 139.0, 138.6, 138.5, 131.0, 129.3, 128.9, 125.3, 124.9, 124.5, 122.5, 121.7, 51.6, 21.4; GC-MS (EI, 70 eV): *m*/*z* = 282 (M^+^, 100), 251 (71), 208 (16), 110 (10); HRMS-ESI (*m*/*z*): [(M + H)^+^] cald for (C_17_H_15_O_2_S)^+^: 283.0787; found: 283.0785. The spectroscopic data were in good agreement with those reported.^10,11^

#### Methyl 2-(4-Bromophenyl)benzo[*b*]thiophene-3-carboxylate (**2c**)

Purified by column chromatography on silica gel using as eluent hexane to 95:5 hexane–AcOEt; yield: 86.7 mg, starting from 91.0 mg of (2-((4-bromophenyl)ethynyl)phenyl)(methyl)sulfane **1c** (83%; Table 1, entry 7). Yellow solid, mp = 82–83 °C. IR (KBr): ν = 1716 (s), 1605 (s), 1435 (s), 1315 (m), 1246 (s), 1180 (m), 1034 (m), 1011 (w), 826 (m), 748 (m) cm^–1^; ^1^H NMR (CDCl_3_, 500 MHz): δ = 8.35 (d, *J* = 8.1, 1H), 7.81 (d, *J* = 8.1, 1H), 7.58–7.54 (m, 1H), 7.50–7.45 (m, 1 H), 7.43–7.36 (m, 3H), 3.78 (s, 3H); ^13^C{^1^H}NMR (CDCl_3_, 125 MHz): δ = 164.2, 150.5, 138.6, 138.4, 133.0, 131.4, 131.1, 125.6, 125.2, 124.8, 123.4, 123.2, 121.8, 51.6; GC-MS (EI, 70 eV): *m*/*z* = 348 [(M + 2)^+^, 79], 346 (M^+^, 75), 317 (32), 315 (32), 251 (7), 236 (100), 208 (29), 163 (22), 133 (19), 104 (27). The spectroscopic data agreed with those reported.^10^

#### Methyl 2-(Thiophen-3-yl)benzo[*b*]thiophene-3-carboxylate (**2d**)

Purified by column chromatography on silica gel using as eluent hexane to 95:5 hexane–AcOEt; yield: 57.9 mg, starting from 69.3 mg of 3-((2-(methylthio)phenyl)ethynyl)thiophene **1d** (70%; Table 1, entry 8). Yellow oil. IR (film): ν = 1713 (s), 1458 (w), 1435 (m), 1350 (m), 1273 (w), 1211 (s), 1169 (w), 1076 (w), 1034 (w), 991 (w), 779 (s) cm^–1^; ^1^H NMR (CDCl_3_, 300 MHz): δ = 8.29 (d, *J* = 8.0, 1H), 7.77 (d, *J* = 8.0, 1H), 7.63–7.53 (m, 1H), 7.48–7.25 (m, 4H), 3.85 (s, 3H); ^13^C{^1^H}NMR (CDCl_3_, 75 MHz): δ = 164.4, 145.8, 138.5, 138.0, 133.8, 128.9, 125.6, 125.4, 125.0, 124.5, 122.4, 121.6, 51.7; GC-MS (EI, 70 eV): *m*/*z* = 276 [(M + 2)^+^, 11], 274 (M^+^, 100), 245 (10), 243 (99), 214 (10), 171 (36); HRMS-ESI (*m*/*z*): [(M + H)^+^] cald for (C_14_H_11_O_2_S_2_)^+^: 275.0195; found: 275.0194.

#### Methyl 2-(Cyclohex-1-en-1-yl)benzo[*b*]thiophene-3-carboxylate (**2e**)

Purified by column chromatography on silica gel using as eluent hexane to 95:5 hexane–AcOEt; yield: 64.9 mg, starting from 68.6 mg of (2-(cyclohex-1-en-1-ylethynyl)phenyl)(methyl)sulfane **1e** (79%; Table 1, entry 9). Yellow oil. IR (film): ν = 1713 (s), 1512 (w), 1458(m), 1435 (m), 1354 (m), 1277 (m), 1215 (s), 1169 (w), 1018 (m), 745 (m) cm^–1^; ^1^H NMR (CDCl_3_, 300 MHz): δ = 8.28 (d, *J* = 8.1, 1H), 7.74 (d, *J* = 8.1, 1H), 7.47–7.26 (m, 2H), 6.03–5.95 (m, 1H), 3.92 (s, 3H), 2.45–2.30 (m, 2H), 2.30–2.16 (m, 2H), 1.86–1.63 (m, 4H); ^13^C{^1^H}NMR (CDCl_3_, 75 MHz): δ = 164.5, 155.6, 138.4, 137.5, 132.6, 130.1, 125.1, 124.6, 124.2, 121.7, 121.4, 51.6, 29.8, 25.7, 22.8, 21.7; GC-MS (EI, 70 eV): *m*/*z* = 272 (M^+^, 76), 240 (100), 213 (36), 185 (50), 184 (42), 178 (19), 172 (14), 171 (25), 147 (24), 115 (26); HRMS-ESI (*m*/*z*): [(M + H)^+^] cald for (C_16_H_17_O_2_S)^+^: 273.0944; found: 273.0952.

#### Methyl 2-Butylbenzo[*b*]thiophene-3-carboxylate (**2f**)

Purified by column chromatography on silica gel using as eluent hexane to 95:5 hexane–AcOEt; yield: 62.1 mg, starting from 61.5 mg of (2-(hex-1-yn-1-yl)phenyl)(methyl)sulfane **1f** (83%; Table 1, entry 10). Yellow oil. IR (film): ν = 1713 (s), 1519 (w), 1458 (m), 1435 (m), 1211 (s), 1092 (w), 1011 (m), 791 (w), 748 (m) cm^–1^; ^1^H NMR (CDCl_3_, 500 MHz): δ = 8.37 (d, *J* = 8.1, 1H), 7.74 (d, *J* = 8.1, 1H), 7.43–7.37 (m, 1H), 7.34–7.28 (m, 1H), 3.96 (s, 3H), 3.30–3.24 (m, 2H), 1.79–1.70 (m, 2H), 1.46 (sextuplet, *J* = 7.4, 2H), 0.97 (t, *J* = 7.4, 3H); ^13^C{^1^H}NMR (CDCl_3_, 125 MHz): δ = 164.4, 158.6, 138.5, 137.2, 125.1, 124.5, 124.3, 122.0, 121.7, 51.4, 33.6, 30.4, 22.6, 13.9; GC-MS (EI, 70 eV): *m*/*z* = 248 (M^+^, 94), 217 (29), 206 (84), 187 (83), 175 (65), 161 (19), 147 (100), 115 (40); HRMS-ESI (*m*/*z*): [(M + H)^+^] cald for (C_14_H_17_O_2_S)^+^: 249.0944; found: 249.0963.

#### Methyl 2-Phenethylbenzo[*b*]thiophene-3-carboxylate (**2g**)

Purified by column chromatography on silica gel using as eluent hexane to 95:5 hexane–AcOEt; yield: 56.2 mg, starting from 75.8 mg of methyl(2-(4-phenylbut-1-yn-1-yl)phenyl)sulfane **1g** (63%; Table 1, entry 11). Yellow oil. IR (film): ν = 1711 (s), 1496 (w), 1454 (m), 1433 (m), 1358 (m), 1215 (s), 1029 (m), 856 (w), 748 (m), 699 (w) cm^–1^; ^1^H NMR (CDCl_3_, 500 MHz): δ = 8.41–8.37 (m, 1 H), 7.75–7.72 (m, 1H), 7.44–7.39 (m, 1H), 7.35–7.27 (m, 3H), 7.26–7.19 (m, 3H); ^13^C{^1^H}NMR (CDCl_3_, 125 MHz): δ = 164.2, 156.8, 140.8, 138.4, 137.3, 128.54, 128.50, 126.3, 125.2, 124.6, 124.5, 122.4, 121.8, 51.5, 37.6, 32.7; GC-MS (EI, 70 eV): *m*/*z* = 296 (M^+^, 44), 264 (14), 236 (4), 205 (100), 175 (35), 147 (11), 91 (25). The spectroscopic data were in good agreement with those reported.^10^

#### Methyl 2-(*tert*-Butyl)benzo[*b*]thiophene-3-carboxylate (**2h**)

Purified by column chromatography on silica gel using as eluent hexane to 95:5 hexane–AcOEt; yield: 42.6 mg, starting from 61.3 mg of (2-(3,3-dimethylbut-1-yn-1-yl)phenyl)(methyl)sulfane **1h** (57%; Table 1, entry 12). Yellow oil. IR (film): ν = 1724 (s), 1458 (w), 1435 (w), 1366 (w), 1342 (w), 1223 (m), 1204 (m), 1034 (w), 984 (w), 733 (w) cm^–1^; ^1^H NMR (CDCl_3_, 300 MHz): δ = 7.77–7.70 (m, 2H), 7.40–7.26 (m, 2H), 3.99 (s, 3H), 1.52 (s, 9H); ^13^C{^1^H}NMR (CDCl_3_, 75 MHz): δ = 167.1, 158.9, 139.1, 136.4, 124.7, 124.3, 123.5, 122.4, 121.6, 52.1, 35.9, 31.4; GC-MS (EI, 70 eV): *m*/*z* = 248 (M^+^, 44), 233 (47), 201 (100), 173 (7), 129 (12), 115 (13); HRMS-ESI (*m*/*z*): [(M + H)^+^] cald for (C_14_H_17_O_2_S)^+^: 249.0944; found: 249.0957.

#### Methyl 5-Methyl-2-phenylbenzo[*b*]thiophene-3-carboxylate (**2i**)

Purified by column chromatography on silica gel using as eluent hexane–Et_2_O from 100:0 to 99.5%–0.5%; yield: 51.6 mg, starting from 71.5 mg of methyl(4-methyl-2-(phenylethynyl)phenyl)sulfane **1i** (61%; Table 1, entry 13). Yellow solid, mp = 92–93 °C. IR (KBr): ν = 1713 (s), 1528 (w), 1443 (m), 1350 (w), 1273 (w), 1234 (m), 1211 (m), 1165 (m), 1080 (w), 1026 (m), 802 (m) cm^–1^; ^1^H NMR (CDCl_3_, 500 MHz): δ = 8.13 (s, 1 H), 7.69 (d, *J* = 8.2, 1H), 7.53–7.47 (m, 2 H), 7.45–7.39 (m, 3 H), 7.26–7.20 (m, 1 H), 3.75 (s, 3 H), 2.51 (s, 3 H); ^13^C{^1^H}NMR (CDCl_3_, 75 MHz): δ = 164.7, 151.8, 138.9, 135.9, 135.3, 134.2, 129.4, 128.8, 128.2, 126.8, 124.4, 121.43, 121.37, 51.5, 21.7; GC-MS (EI, 70 eV): *m*/*z* = 282 (M^+^, 89), 251 (100), 224 (6), 208 (22), 179 (12); HRMS-ESI (*m*/*z*): [(M + H)^+^] cald for (C_17_H_15_O_2_S)^+^: 283.0787; found: 283.0792.

#### Methyl 5-Fluoro-2-phenylbenzo[*b*]thiophene-3-carboxylate (**2j**)

Purified by column chromatography on silica gel using as eluent hexane–Et_2_O from 100:0 to 99.5%–0.5%; yield: 53.2 mg, starting from 72.6 mg of (4-fluoro-2-(phenylethynyl)phenyl)(methyl)sulfane **1j** (62%; Table 1, entry 14). Yellow oil. IR (film): ν = 1713 (s), 1605 (m), 1566 (w), 1443 (s), 1350 (m), 1265 (m), 1227 (s), 1165 (m), 1026 (w), 748 (w) cm^–1^; ^1^H NMR (CDCl_3_, 500 MHz): δ = 8.07 (dd, *J* = 10.4, 2.4, 1 H), 7.73 (dd, *J* = 8.8, 4.9, 1H), 7.52–7.47 (m, 2 H), 7.46–7.41 (m, 3 H), 7.14 (td, *J* = 8.8, 2.4, 1 H), 3.76 (s, 3 H); ^13^C{^1^H}NMR (CDCl_3_, 75 MHz): δ = 164.0, 161.3 (d, *J* = 242.4), 154.7, 139.8 (d, *J* = 10.1), 133.9 (d, *J* = 18.2), 133.8, 129.2, 128.9, 128.2, 122.9 (d, *J* = 9.4), 113.9 (*J* = 25.4), 110.7 (d, *J* = 25.0), 51.6; GC-MS (EI, 70 eV): *m*/*z* = 286 (M^+^, 80), 255 (100), 226 (30), 207 (8), 183 (38); HRMS-ESI (*m*/*z*): [(M + H)^+^] cald for (C_16_H_12_FO_2_S)^+^: 287.0537; found: 287.0540.

#### Methyl 6-Fluoro-2-phenylbenzo[*b*]thiophene-3-carboxylate (**2k**)

Purified by column chromatography on silica gel using as eluent hexane–Et_2_O from 100:0 to 99.5%–0.5%; yield: 63.4 mg, starting from 72.7 mg of (5-fluoro-2-(phenylethynyl)phenyl)(methyl)sulfane **1k** (74%; Table 1, entry 15). Yellow solid, mp 90–93 °C. IR (film): ν = 1706 (s), 1597 (w), 1535 (w), 1466 (m), 1435 (w), 1358 (w), 1242 (m), 1204 (m), 1065 (w), 748 (m) cm^–1^; ^1^H NMR (CDCl_3_, 500 MHz): δ = 8.32 (dist dd, *J* = 10.8, 5.2, 1 H), 7.58–7.39 (m, 6 H), 7.22 (dist td, *J* = 10.9, 1.9, 1 H), 3.75 (s, 3 H); ^13^C{^1^H}NMR (CDCl_3_, 75 MHz): δ = 164.2, 160.5 (d, *J* = 246.1), 151.6, 139.4 (d, *J* = 10.0), 135.0, 133.8, 129.4, 129.0, 128.2, 126.0 (d, *J* = 7.3), 122.4, 114.3 (*J* = 23.5), 107.8 (d, *J* = 25.4), 51.6; GC-MS (EI, 70 eV): *m*/*z* = 286 (M^+^, 88), 255 (100), 226 (28), 183 (41), 113 (18); HRMS-ESI (*m*/*z*): [(M + H)^+^] cald for (C_16_H_12_FO_2_S)^+^: 287.0537; found: 287.0537.

### Carbonylation of Methyl(2-(phenylethynyl)phenyl)sulfane **1a** under Unoptimized Conditions Leading to a Mixture of **2a** and **3a** (Table S1, entry 9, Supporting Information)

A 250 mL stainless-steel autoclave was charged in the presence of air with PdI_2_ (5.4 mg, 0.015 mmol), KI (125 mg, 0.75 mmol), and substrate **1a** (67.4 mg, 0.30 mmol) in MeOH (6 mL). The autoclave was sealed and, while the mixture was stirred, the autoclave was pressurized with CO (32 atm) and air (up to 40 atm). After being stirred at 80 °C (jacketed autoclave with circulating thermic fluid) for 15 h, the autoclave was cooled, degassed, and opened. The solvent was evaporated, and the products **2a** and **3a** were separated by column chromatography on silica gel using as eluent hexane to 95:5 hexane–AcOEt (order of elution: **2a**, **3a**). While methyl 2-phenylbenzo[*b*]thiophene-3-carboxylate **2a** was obtained pure, dimethyl 2-(2-(methylthio)phenyl)-3-phenylmaleate **3a** was isolated together with several eluting solvent impurities and could not be purified further. However, the ^1^H NMR spectrum clearly showed all the expected signals, including those related to the different methoxycarbonyl groups, and the GC-MS spectrum evidenced the expected molecular ion, as detailed below.

#### Dimethyl 2-(2-(Methylthio)phenyl)-3-phenylmaleate (**3a**)

Purity: ca. 60%, by ^1^H NMR. Yield: 23.6 mg of crude product, corresponding to ca. 14 mg of **3a**, starting from 67.4 mg of methyl(2-(phenylethynyl)phenyl)sulfane **1a** (14%; Table S1, entry 9). Yellow oil. IR (film): ν = 1724 (s), 1462 (w), 1435 (m), 1254 (w), 1142 (m), 745 (m), cm^–1^ ; ^1^H NMR (CDCl_3_, 300 MHz): δ = 7.76–7.15 (m, 9 H), 3.64 (s, 3 H), 3.53 (s, 3H), 2.41 (s, 3H); GC-MS (EI, 70 eV): *m*/*z* = 342 (M^+^, 58), 297 (100), 283 (48), 268 (29), 251 (21), 237 (59), 223 (60), 208 (25), 165 (29), 149 (33), 147 (32), 91 (23), 59 (33).

### Procedure for the Synthesis of Methyl 2-Phenylbenzo[*b*]thiophene-3-carboxylate **2a** in Larger Scale

A 250 mL stainless steel autoclave was charged in the presence of air with PdI_2_ (20.2 mg, 0.056 mmol), KI (465.1 mg, 2.80 mmol), and a solution of methyl(2-(phenylethynyl)phenyl)sulfane **1a** (251.3 mg, 1.12 mmol) in MeOH (56 mL). The autoclave was sealed and, while the mixture was stirred, the autoclave was pressurized with CO (32 atm) and air (up to 40 atm). After being stirred at 80 °C (jacketed autoclave with circulating thermic fluid) for 24 h, the autoclave was cooled, degassed, and opened. The solvent was evaporated, and product **2a** was purified by column chromatography on silica gel using as eluent hexane to hexane–AcOEt 95:5 (yield: 244 mg, 81% based on starting **1a**).

### General Procedure for the Synthesis of Benzo[*b*]thiophen-3-carboxylic Esters **2a**, **2b**, **2d**, and **2g** in BmimBF_4_ (Table S2, Supporting Information)

A 250 mL stainless-steel autoclave was charged in the presence of air with PdI_2_ (5.4 mg, 0.015 mmol), KI (125 mg, 0.75 mmol), a solution of substrate **1** (0.30 mmol; **1a**, 67.4 mg; **1b**, 71.5 mg; **1d**, 69.0 mg; **1g**, 75.6 mg) in MeOH (1.5 mL), and BmimBF_4_ (4.5 mL). The autoclave was sealed and, while the mixture was stirred, the autoclave was pressurized with CO (32 atm) and air (up to 40 atm). After being stirred at 100 °C (jacketed autoclave with circulating thermic fluid) for 36 h, the autoclave was cooled, degassed, and opened. The mixture was then extracted with Et_2_O (6 × 10 mL), and the residue (still containing the catalyst dissolved in the ionic liquid) was used as such for the next recycle (see below). The collected ethereal phases were concentrated, and the product was purified by column chromatography on silica gel using as eluent hexane to hexane–AcOEt 95:5 to give pure benzo[*b*]thiophen-3-carboxylic esters **2a**, **2b**, **2d**, and **2g**. The isolated yields obtained in each experiment are given in Table S2 (Supporting Information).

#### Catalyst Recycling Procedure

After removal of Et_2_O under vacuum, the residue obtained as described above, still containing the catalyst dissolved in the ionic liquid, was transferred into the autoclave. A solution of **1** (0.30 mmol) in MeOH (1.5 mL) was added, and then the same procedure described above was followed.

## Supporting Information Available

Tables S1 and optimization of reaction conditions, Table S2, preparation and characterization of substrates **1a**–**k**, copies of HRMS, ^1^H NMR, and ^13^C{^1^H}NMR spectra for substrates **1a**–**k** and products **2a**–**k**, **2a′**, **2a′′** (PDF)
