# Supplementary material for: Synthesis of Benzothiophene-3-carboxylic Esters by Palladium Iodide-Catalyzed Oxidative Cyclization–Deprotection–Alkoxycarbonylation Sequence under Aerobic Conditions
Source: J Org Chem. 2022 May 10;88(8):5180–6. doi: 10.1021/acs.joc.2c00686 (PMC10127270; doi:10.1021/acs.joc.2c00686)
Supplement: Supplementary file 1 — jo2c00686_si_001.pdf [file jo2c00686_si_001.pdf]

Supporting Information for

Synthesis of Benzothiophene-3-Carboxylic Esters by Palladium Iodide Catalyzed Oxidative Cyclization-Deprotection-Alkoxycarbonylation Sequence under Aerobic Conditions

Raffaella Mancuso,* Simona Cuglietta, Romina Strangis, and Bartolo Gabriele*

Laboratory of Industrial and Synthetic Organic Chemistry (LISOC), Department of Chemistry and Chemical Technologies, University of Calabria, Via Pietro Bucci 12/C, 87036 Arcavacata di Rende (CS), Italy

raffaella.mancuso@unical.it; bartolo.gabriele@unical.it

Table of Contents

**Page S2–S3**	**Table S1 and Optimization of Reaction Conditions**
**Page S4**	**Table S2**
**Pages S5–S7**	**Preparation and Characterization of Substrates 1a-k**
**Pages S8–S15**	**Copies of HRMS spectra**
**Pages S16−S63**	**Copies of ^{1}H NMR and ^{13}C{^{1}H}NMR Spectra**
**Page S64**	**References**

Table S1 and Optimization of Reaction Conditions

Table S1. PdI_2/KI-catalyzed oxidative carbonylation of methyl(2-(phenylethynyl)phenyl)sulfane **1a** under different conditions.[a]

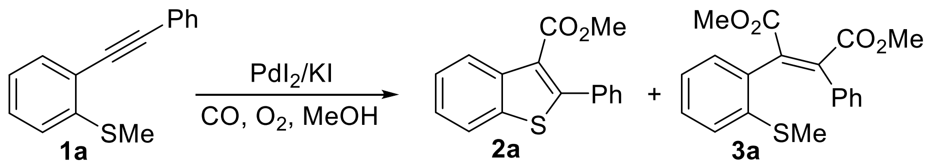

Entry	KI:**1a**:PdI_2 molar ratio	Substrate concentration[b]	T (°C)	P_{CO} (atm)	P_{air} (atm)	Conversion of **1a** (%)[c]	Yield of **2a** (%)[d]	Yield of **3a** (%)[e]
1	100:20:1	0.02	80	32	8	91	43	7
2	150:20:1	0.02	80	32	8	91	45	7
3	50:20:1	0.02	80	32	8	82	65	traces
4	25:20:1	0.02	80	32	8	68	45	7
5	50:20:1	0.02	80	48	12	100	55	10
6	50:20:1	0.02	80	16	4	65	36	12
7	50:20:1	0.02	100	32	8	100	55	5
8	50:20:1	0.02	70	32	8	50	18	2
9	50:20:1	0.05	80	32	8	100	49	20[f]

[a] All reactions were carried out in MeOH for 15 h.
[b] Mmol of **1a** per mL of solvent.
[c] Determined by isolation of unreacted **1a**.
[d] Isolated yield based on starting **1a**.
[e] GLC yield based on starting **1a**.
[f] Isolated yield was 14%, based on starting **1a**. See the Experimental Section for details.

Entry 1 of Table S1 reports the results obtained with the first experiment, carried out in MeOH for 15 h at 80 °C under 40 atm of a 4:1 mixture of CO−air, using 5 mol% of PdI_2 in the presence of 5 equiv of KI (substrate initial concentration, 0.02 mmol / mL of MeOH): carbonylation products methyl 2-phenylbenzo[*b*]thiophene-3-carboxylate **2a** and maleic diester **3a** were obtained in 43% isolated yield and 7% GLC yield, respectively. An augment of KI amount to 7.5 equiv led to practically the same results as those of the parent experiment (Table S1, entry 2). On the other hand, a more selective reaction toward **2a** was observed by lowering the KI equiv to 2.5: in fact, **2a** was isolated in 65% yield, with only traces of **3a**, at 82% **1a** conversion (Table S1, entry 3). A further decrease of the amount of KI to 1.25 equiv, however, led to inferior results (Table S1, entry 4). Substrate conversion was faster when the process was carried out under a higher total pressure (60 atm), although selectivity toward **2a** did not improve (substrate conversion was quantitate, with yields of **2a** and **3a** of 55% and 10%, respectively; Table S1, entry 5). Decreasing the pressure to 20 atm caused a quite slower reaction, still with no improvement in **2a** selectivity (Table S1, entry 6). Quite predictably, a total **1a** conversion was obtained at 100 °C, with yields of **2a** and **3a** of 55% and 5%, respectively (Table S1, entry 7), while at 70 °C the substrate conversion was only 50% (Table S1, entry 8). Finally, quantitative substrate conversion was also observed when performing the reaction under more concentrated conditions (0.05 mmol of **1a** per mL of MeOH), although the **2a**:**3a** ratio decreased (yields were 49% and 20%, respectively, Table S1, entry 9).

Table S2. Synthesis of benzo[*b*]thiophene-3-carboxylic esters **2** by PdI_2/KI-catalyzed methoxycarbonylation of 2-(methylthio)phenylacetylenes **1** in $BmimBF_4$.[a]

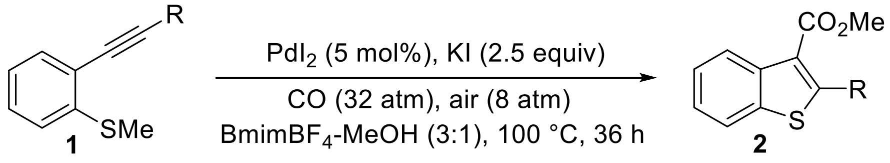

Entry	1	2	Yield of **2**[b] (%)					
			Run 1	Run 2	Run 3	Run 4	Run 5	Run 6
1	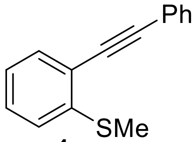 **1a**	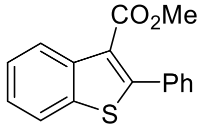 **2a**	68	67	67	65	66	64
2	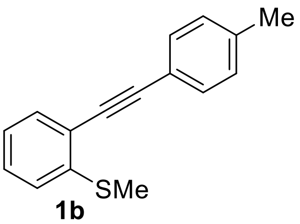 **1b**	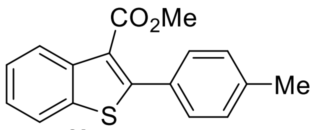 **2b**	65	66	65	64		
3	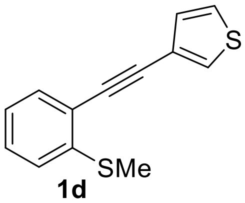 **1d**	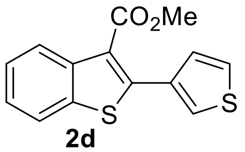 **2d**	55	53	51	53		
4	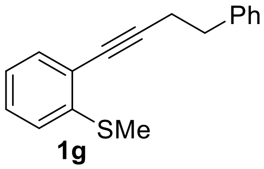 **1g**	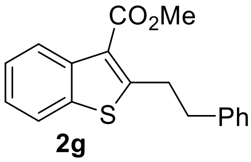 **2g**	52	52	50	51		

[a] All reactions were carried out in a $BmimBF_4$-MeOH mixture (3:1, v/v) as the solvent (0.05 mmol of **1** per mL of solvent), in the presence of PdI_2 (5 mol%) and KI (2.5 equiv) at 100 °C for 36 h, under 40 atm of a 4:1 mixture of CO–air. Substrate conversion was quantitative in all cases.

[b] Based on starting **1**. The first run corresponds to the parent experiment, the next runs to recycles. See the Experimental Section for details.

Preparation and Characterization of Substrates 1a-h

Methyl(2-alkynyl)phenyl)sulfanes **1a-h** were prepared by Sonogashira coupling of 2-iodothioanisole (commercially available), (2-bromo-4-methylphenyl)(methyl)sulfane (prepared by methylation of commercially available 2-bromo-4-methylbenzenethiol, according to a literature procedure),[1] (2-bromo-4-fluorophenyl)(methyl)sulfane (prepared by methylation of commercially available 2-bromo-4-fluorobenzenethiol, according to a literature procedure),[1] and (2-bromo-5-fluorophenyl)(methyl)sulfane (prepared by methylation of commercially available 2-bromo-5-fluorobenzenethiol, according to a literature procedure),[1] with terminal alkynes, as described below.

General Procedure for the Synthesis of Methyl(2-alkynyl)phenyl)sulfanes 1a-k

The whole procedure was carried out under nitrogen. A solution of 2-iodothioanisole (1.0 g, 4.0 mmol), $PdCl_2(PPh_3)_2$ (60 mg, 0.085 mmol), CuI (20 mg, 0.11 mmol), and the terminal alkyne (4.8 mmol; phenylacetylene, 490 mg; 1-ethynyl-4-methylbenzene, 560 mg; 1-bromo-4-ethynylbenzene, 868 mg; 3-ethynylthiophene, 520 mg; 1-ethynylcyclohexene, 510 mg; 1-hexyne, 395 mg; but-3-yn-1-ylbenzene, 625 mg; 3,3-dimethyl-1-butyne, 395 g) in anhydrous triethylamine (16 mL) was allowed to stir under nitrogen at 25 °C for 5 h. Water (50 mL) was then added, and the mixture extracted with diethyl ether (3 × 50 mL). The organic layer was washed with a saturated solution of NH_4Cl (100 mL) and water until neutral pH. After drying over Na_2SO_4, the solvent was evaporated, and the residue purified by column chromatography on silica gel using hexane-AcOEt (9:1) as eluent.

Procedure for the Synthesis of Methyl(2-alkynyl)phenyl)sulfanes 1i-k

The whole procedure was carried out under nitrogen. A solution of substituited 2-iodothioanisole (1.5 mmol; 2-bromo-5-methylbenzenethiol, 304 mg; 2-bromo-4-fluorobenzenethiol, 310 mg; 2-bromo-5-fluorobenzenethiol, 310 mg), $PdCl_2(PPh_3)_2$ (105.5 mg, 0.15 mmol), CuI (34.5 mg, 0.18 mmol), and phenylacetylene (3.0 mmol; 306.5 mg) in anhydrous diisopropylamine (15 mL) was allowed to stir under nitrogen at 80 °C for 24 h. Water (50 mL) was then added, and the mixture extracted with diethyl ether (3 × 50 mL). The organic layer was washed with a saturated solution of NH_4Cl (100 mL) and water until neutral pH. After drying over Na_2SO_4, the solvent was evaporated, and the residue purified by column chromatography on silica gel using hexane to 99:1 hexane–AcOEt as eluent.

Methyl(2-(phenylethynyl)phenyl)sulfane (**1a**). Purified by column chromatography on silica gel using as eluent hexane–AcOEt (9:1); yield: 720 mg, 80% based on 2-iodothioanisole. Yellow oil. IR (film): ν = 2214 (vw), 1597 (w), 1489 (m), 1462 (m), 1435 (m), 1254 (w), 1069 (m), 752 (s), 667 (m) cm^{-1}; ^1H NMR ($CDCl_3$, 300 MHz): δ = 7.62-7.54 (m, 2H), 7.51-7.44 (m, 1H), 7.39-7.05 (m, 6H), 2.49 (s, 3H); $^{13}C\{^1H\}$NMR ($CDCl_3$, 75 MHz): δ = 141.7, 132.2, 131.6, 128.8, 128.4, 128.3, 124.2, 124.0, 123.2, 121.2, 95.8, 86.9, 15.0; GC-MS (EI, 70 eV): *m/z* = 224 (M^+, 100), 223 (78), 221 (20), 208 (19), 178 (11), 165 (11), 147 (49); HRMS-ESI (*m/z*): $[(M+H)^+]$ cald for $(C_{15}H_{13}S)^+$: 225.0732; found: 255.0751. The spectroscopic data agreed with those reported.[2]

Methyl(2-(p-tolylethynyl)phenyl)sulfane (**1b**). Purified by column chromatography on silica gel using as eluent hexane–AcOEt (9:1); yield: 868 mg, 91% based on 2-iodothioanisole. Yellow solid, mp = 41-43 °C. IR (KBr): ν = 2210 (w), 1582 (w), 1508 (m), 1462 (m), 1435 (m), 1065 (w), 818 (s), 748 (s) cm^{-1}; ^1H NMR ($CDCl_3$, 300 MHz): δ = 7.57-7.41 (m, 1H), 7.46 (dist d, *J* = 8.0, 2H), 7.31-7.02 (m, 3H), 7.14 (dist d, *J* = 8.0, 2H), 2.48 (s, 3H), 2.35 (s, 3H); $^{13}C\{^1H\}$NMR ($CDCl_3$, 75 MHz): δ = 141.6, 138.5, 132.1, 131.5, 129.1, 128.6, 124.2, 124.1, 121.5, 120.1, 96.1, 86.3, 21.5, 15.1; GC-MS (EI, 70 eV): *m/z* = 238 (M^+, 100), 237 (71), 223 (27), 221 (39), 189 (13), 178 (12), 183 (8), 147 (54), 118 (22); HRMS-ESI (*m/z*): $[(M+H)^+]$ cald for $(C_{16}H_{15}S)^+$: 239.0889; found: 239.0899. The spectroscopic data were in good agreement with those reported.[3]

(2-((4-Bromophenyl)ethynyl)phenyl)(methyl)sulfane (**1c**). Purified by column chromatography on silica gel using as eluent hexane–AcOEt (9:1); yield: 1.06 g, 87% based on 2-iodothioanisole. Yellow solid, mp = 52-53 °C. IR (KBr): ν = 2214 (vw), 1578 (w), 1485 (s), 1435 (m), 1393 (m), 1069 (m), 1037 (w), 1011 (m), 822 (s), 748 (s) cm^{-1}; ^{1}H NMR ($CDCl_3$, 300 MHz): δ = 7.50-7.38 (m, 5H), 7.35-7.26 (m, 1H), 7.20-7.06 (m, 2H), 2.50 (s, 3H); $^{13}C\{^{1}H\}$NMR ($CDCl_3$, 75 MHz): δ = 141.8, 133.0, 132.2, 131.6, 129.0, 124.3, 124.0, 122.6, 122.1, 120.9, 94.7, 88.0, 15.0; GC-MS (EI, 70 eV): *m/z* = 304 [$(M+2)^+$, 100], 303 (82), 302 (M^+, 98), 301 (64), 223 (28), 222 (42), 221 (68), 208 (38), 176 (16), 163 (33), 147 (92), 111 (63); HRMS-ESI (*m/z*): [$(M+H)^+$] cald for $(C_{15}H_{12}BrS)^+$: 302.9838; found: 302.9822. The spectroscopic data were in good agreement with those reported.[3]

3-((2-(Methylthio)phenyl)ethynyl)thiophene (**1d**). Purified by column chromatography on silica gel using as eluent hexane–AcOEt (9:1); yield: 785 mg, 85% based on 2-iodothioanisole. Yellow oil. IR (film): ν = 2207 (w), 1582 (w), 1462 (m), 1435 (m), 1354 (w), 1123 (w), 1065 (m), 941 (w), 868 (m), 833 (m), 779 (s), 748 (s) cm^{-1}; ^{1}H NMR ($CDCl_3$, 300 MHz): δ = 7.57-7.52 (m, 1H), 7.49-7.42 (m, 1H), 7.32-7.18 (m, 3H), 7.18-7.04 (m, 2H), 2.48 (s, 3H); $^{13}C\{^{1}H\}$NMR ($CDCl_3$, 75 MHz): δ = 141.5, 132.2, 129.9, 128.8, 128.7, 125.3, 124.2, 124.0, 122.1, 121.2, 91.0, 86.3, 15.0; GC-MS (EI, 70 eV): *m/z* = 332 [$(M+2)^+$, 10], 231 (23), 230 (M^+, 100), 214 (8), 197 (20), 184 (19), 171 (25), 147 (19); HRMS-ESI (*m/z*): [$(M+H)^+$] cald for $(C_{13}H_{11}S_2)^+$: 231.0297; found: 231.0304. The spectroscopic data were in goog agreement with those reported.[4]

(2-(Cyclohex-1-en-1-ylethynyl)phenyl)(methyl)sulfane (**1e**). Purified by column chromatography on silica gel using as eluent hexane–AcOEt (9:1); yield: 640 mg, 70% based on 2-iodothioanisole. Yellow oil. IR (film): ν = 2195 (w), 1462 (s), 1435 (s), 1064 (w), 1038 (w), 918 (w), 841 (w), 748 (s) cm^{-1}; ^{1}H NMR ($CDCl_3$, 300 MHz): δ = 7.41-7.33 (m, 1H), 7.29-7.20 (m, 1H, aromatic), 7.16-7.01 (m, 2H, aromatic), 6.30-6.23 (m, 1H), 2.46 (s, 3H), 2.32-2.23 (m, 2H), 2.20-2.11 (m, 2H), 1.74-1.56 (m, 4H); $^{13}C\{^{1}H\}$NMR ($CDCl_3$, 75 MHz): δ = 141.2, 135.5, 132.0, 128.2, 124.1, 123.8, 121.7, 120.7, 97.9, 84.2, 29.1, 25.8, 22.3, 21.5, 15.0; GC-MS (EI, 70 eV): *m/z* = 228 (M^+,100), 213 (27), 185 (39), 171 (16), 165 (17), 147 (62), 79 (35); HRMS-ESI (*m/z*): [$(M+H)^+$] cald for $(C_{15}H_{17}S)^+$: 229.1045; found: 229.1038. The spectroscopic data were in good agreement with those reported.[5]

(2-(Hex-1-yn-1-yl)phenyl)(methyl)sulfane (**1f**). Purified by column chromatography on silica gel using as eluent hexane–AcOEt (9:1); yield: 599 mg, 73% based on 2-iodothioanisole. Yellow oil. IR (film): ν = 2228 (w), 1584 (w), 1464 (s), 1435 (m), 1076 (w), 1040 (w), 750 (s) cm^{-1}; ^{1}H NMR ($CDCl_3$, 300 MHz): δ = 7.35 (dist d, *J* = 7.5, 1H), 7.27-7.16 (m, 1H), 7.10 (dist d, *J* = 8.0, 1H), 7.03 (dist t, *J* = 7.5, 1H), 2.52-2.40 (m, 2H), 2.46 (s, 3H), 1.70-1.44 (m, 4H), 1.00-0.89 (m, 3H, Me); $^{13}C\{^{1}H\}$NMR ($CDCl_3$, 75 MHz): δ = 141.2, 132.3, 128.0, 124.1, 123.8, 122.1, 97.4, 78.1, 30.8, 22.0, 19.4, 15.0, 13.7; GC-MS (EI, 70 eV): *m/z* = 204 (M^+, 6), 189 (16), 162 (16), 147 (100), 128 (26), 115 (17); HRMS-ESI (*m/z*): [$(M+H)^+$] cald for $(C_{13}H_{17}S)^+$: 205.1045; found: 210.1055. The spectroscopic data were in good agreement with those reported.[5]

Methyl(2-(4-phenylbut-1-yn-1-yl)phenyl)sulfane (**1g**). Purified by column chromatography on silica gel using as eluent hexane–AcOEt (9:1); yield: 755 mg, 74% based on 2-iodothioanisole. Yellow oil. IR (film): ν = 2226 (vw), 1602 (w), 1496 (m), 1465 (m), 1453 (m), 1435 (m), 1076 (m), 749 (m), 698 (w) cm^{-1}; ^{1}H NMR ($CDCl_3$, 500 MHz): δ = 7.35-7.28 (m, 5H), 7.26-7.19 (m, 2H), 7.11 (dist d, *J* = 8.0, 1H), 7.07-7.01 (m, 1 H), 2.97 (dist t, *J* = 7.5, 2 H), 2.78 (dist t, *J* = 7.5, 2H), 2.45 (s, 3H); $^{13}C\{^{1}H\}$NMR ($CDCl_3$, 125 MHz): δ = 141.2, 140.7, 132.4, 128.6, 128.4, 128.2, 126.3, 124.2, 124.0, 122.0, 96.4, 78.8, 35.2, 21.9, 15.1; GC-MS (EI, 70 eV): *m/z* = 252 (M^+, 25), 251 (75), 237 (89), 222 (25), 204 (25), 128 (100), 115 (26), 91 (67); HRMS-ESI (*m/z*): [$(M+H)^+$] cald for $(C_{17}H_{17}S)^+$: 253.1045; found: 253.1066. The spectroscopic data agreed with those reported.[6]

(2-(3,3-Dimethylbut-1-yn-1-yl)phenyl)(methyl)sulfane (**1h**). Purified by column chromatography on silica gel using as eluent hexane–AcOEt (9:1); yield: 557 mg, 68% based on 2-iodothioanisole. Yellow oil. IR (film): ν = 2235 (w), 1584 (m), 1462 (s), 1435 (m), 1362 (w), 1292 (m), 1255 (w), 1199 (m), 1070 (w), 1038 (w), 748 (s) cm^{-1}; ^{1}H NMR ($CDCl_3$, 500 MHz): δ = 7.33 (dd, *J* = 7.6, 1.5, 1H), 7.22 (dist td, *J* = 7.6, 1.5, 1H), 7.09 (dist d, br, *J* = 7.6, 1H), 7.03 (td, *J* = 7.6, 1.3, 1H), 2.45 (s, 3H), 1.36 (s, 9H); $^{13}C\{^{1}H\}$NMR ($CDCl_3$, 125 MHz): δ = 141.3, 132.0, 127.9, 124.0, 123.6, 121.9, 105.5, 76.6, 31.0, 28.3, 14.9; GC-MS (EI, 70 eV): *m/z* = 204 (M^+, 100), 189

(63), 174 (62), 161 (29), 156 (29), 149 (55), 147 (51), 115 (44); HRMS-ESI (*m/z*): [(M+H)$^+$] cald for $(C_{13}H_{17}S)^+$: 205.1045; found: 205.1058. The spectroscopic data agreed with those reported.[7]

Methyl(4-methyl-2-(phenylethynyl)phenyl)sulfane (**1i**). Yield: 322.0 mg, 90% based on 2-bromo-5-methylbenzenethiol. Yellow oil. IR (film): ν = 2207 (vw), 1597 (m), 1489 (m), 1435 (s), 1065 (w), 887 (w), 810 (m), 756 (s), 687 (m) cm^{-1}; ^{1}H NMR ($CDCl_3$, 300 MHz): δ = 7.61-7.52 (m, 2 H), 7.40-7.28 (m, 4 H), 7.13-7.05 (m, 2 H), 2.49 (s, 3 H), 2.30 (s, 3 H); ^{13}C{^{1}H}NMR ($CDCl_3$, 75 MHz): δ = 138.0, 134.3, 132.9, 131.6, 129.8, 128.3, 125.0, 123.4, 121.7; GC-MS (EI, 70 eV): *m/z* = 238 (M$^+$, 100), 237 (70), 221 (32), 189 (11), 161 (46); HRMS-ESI (*m/z*): [(M+H)$^+$] cald for $(C_{16}H_{15}S)^+$: 239.0889; found: 239.0894. The spectroscopic data agreed with those reported.[8]

4-Fluoro-2-(phenylethynyl)phenyl(methyl)sulfane (**1j**). Yield: 345.0 mg, 95% based on 2-bromo-5-fluorobenzenethiol. Yellow oil. IR (film): ν = 2214 (w), 1597 (m), 1458 (s), 1258 (m), 1230 (m), 1111 (m), 949 (w), 872 (w), 810 (w), 756 (m), 687 (m) cm^{-1}; ^{1}H NMR ($CDCl_3$, 300 MHz): δ = 7.62-7.47 (m, 2 H, Ph), 7.41-7.26 (m, 3 H, Ph), 7.21 (dd, *J* = 8.8, 2.7, H-6), 7.14 (dd, *J* = 8.8, 5.3, H-3), 7.00 (td, *J* = 8.4, 2.7, H-5), 2.59 (s, 3 H, Me); ^{13}C{^{1}H}NMR ($CDCl_3$, 75 MHz): δ = 160.2 (d, *J* = 244.6, C-4), 143.2 (C-1), 136.8 (C-2), 131.7 (Ph), 128.8 (Ph), 128.4 (Ph), 126.8 (d, *J* = 8.0, C-6), 122.8 (Ph), 119.0 (d, *J* = 23.2, C-3), 116.2 (d, *J* = 21.9, C-5), 96.5, 86.0, 15.9; GC-MS (EI, 70 eV): *m/z* = 242 (M$^+$, 100), 241 (70), 226 (22), 183 (18), 165 (52); HRMS-ESI (*m/z*): [(M+H)$^+$] cald for $(C_{15}H_{12}FS)^+$: 243.0638; found: 243.0644.

5-Fluoro-2-(phenylethynyl)phenyl(methyl)sulfane (**1k**). Yield: 334.5 mg, 92% based on 2-bromo-4-fluorobenzenethiol. Yellow oil. IR (film): ν = 2207 (w), 1589 (s), 1497 (m), 1435 (m), 1389 (w), 1258 (m), 1204 (m), 1257 (w), 957 (w), 910 (m), 849 (m), 810 (m), 756 (m) cm^{-1}; ^{1}H NMR ($CDCl_3$, 500 MHz): δ = 7.59-7.51 (m, 2 H, phenyl ring), 7.48 (dd, J_{HH} = 8.4, J_{HF} = 5.9, 1 H, H-3), 7.37-7.28 (m, 3 H, phenyl ring), 6.85 (dd, J_{HF} = 9.7, J_{HH} = 2.2, 1 H, H-6), 6.78 (td, J_{HH} = J_{HF} = 8.4, J_{HH} = 2.2, 1 H, H-4); ^{13}C{^{1}H}NMR ($CDCl_3$, 75 MHz): δ = 163.0 (d, *J* = 250.8, C-5), 144.8 (d, *J* = 8.3, C-1), 143.2 (Ph), 133.7 (d, *J* = 8.7, C-3), 128.5 (Ph), 128.4 (Ph), 122.9 (Ph), 117.1 (C-2), 111.3 (d, *J* = 22.2, C-6), 111.1 (d, *J* = 26.4, C-4), 95.6, 85.9, 15.0; GC-MS (EI, 70 eV): *m/z* = 242 (M$^+$, 100), 241 (75), 183 (13), 165 (44); HRMS-ESI (*m/z*): [(M+H)$^+$] cald for $(C_{15}H_{12}FS)^+$: 243.0638; found: 243.0641.

Copies of HRMS spectra

Methyl(2-(phenylethynyl)phenyl)sulfane (**1a**)

HRMS (ESI-TOF) *m/z*: $[M+H]^+$ calcd for $(C_{15}H_{13}S)^+$: 225.0732; found, 225.0751

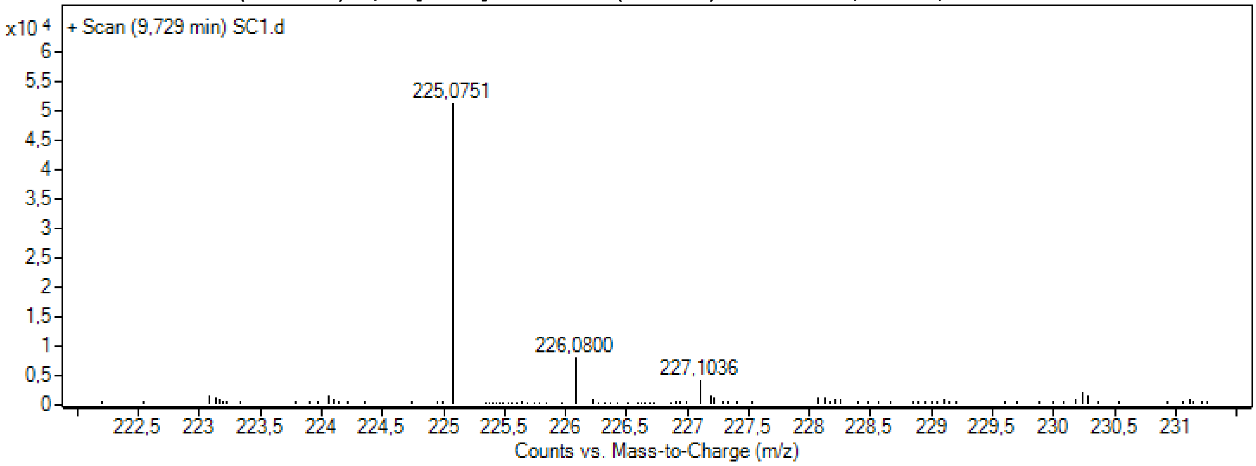

Methyl(2-(*p*-tolylethynyl)phenyl)sulfane (**1b**)

HRMS (ESI-TOF) *m/z*: $[M+H]^+$ calcd for $(C_{16}H_{15}S)^+$: 239.0889; found, 239.0899

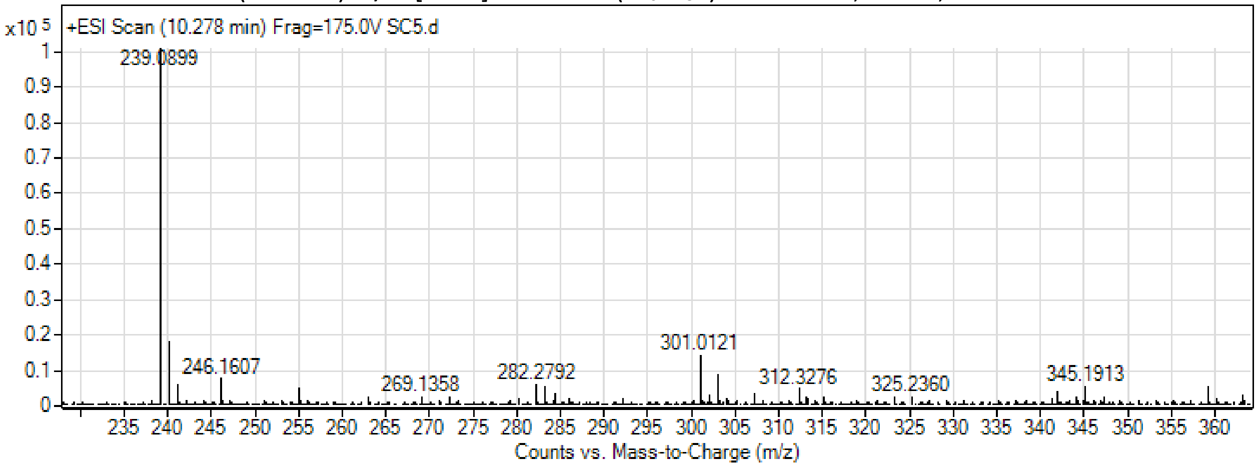

(2-((4-Bromophenyl)ethynyl)phenyl)(methyl)sulfane (**1c**)

HRMS (ESI-TOF) *m/z*: $[M+H]^+$ calcd for $(C_{15}H_{12}BrS)^+$: 302.9838; found, 302.9822

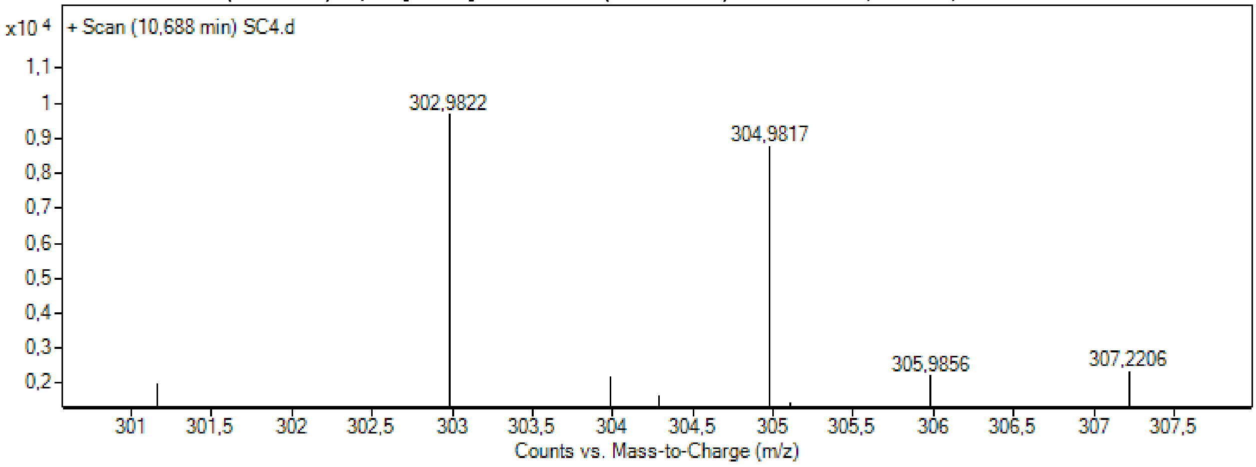

3-((2-(Methylthio)phenyl)ethynyl)thiophene (**1d**)
HRMS (ESI-TOF) *m/z*: $[M+H]^+$ calcd for $(C_{13}H_{11}S_2)^+$: 231.0297; found: 231.0304

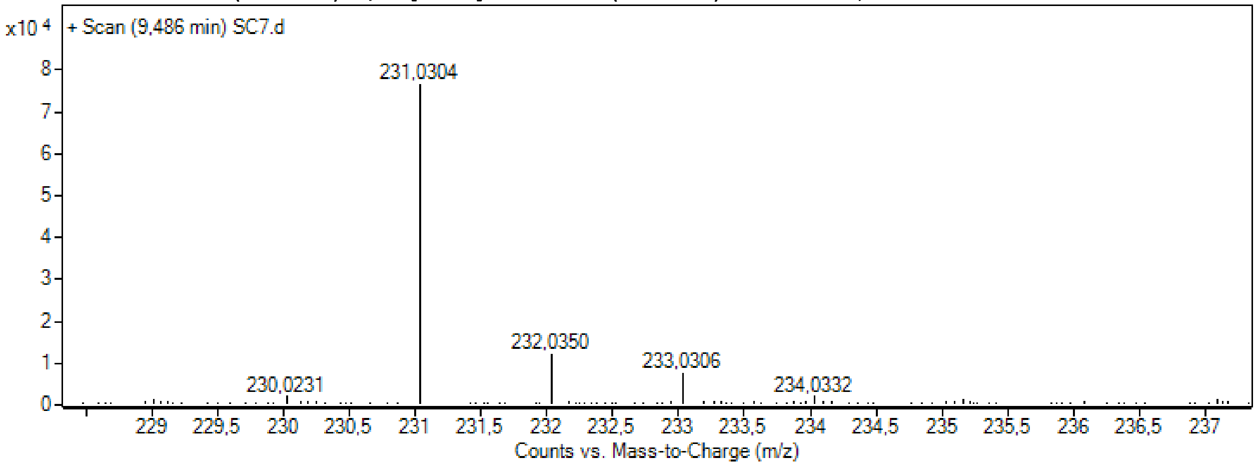

(2-(Cyclohex-1-en-1-ylethynyl)phenyl)(methyl)sulfane (**1e**)
HRMS (ESI-TOF) *m/z*: $[M+H]^+$ calcd for $(C_{15}H_{17}S)^+$: 229.1045; found: 229.1038

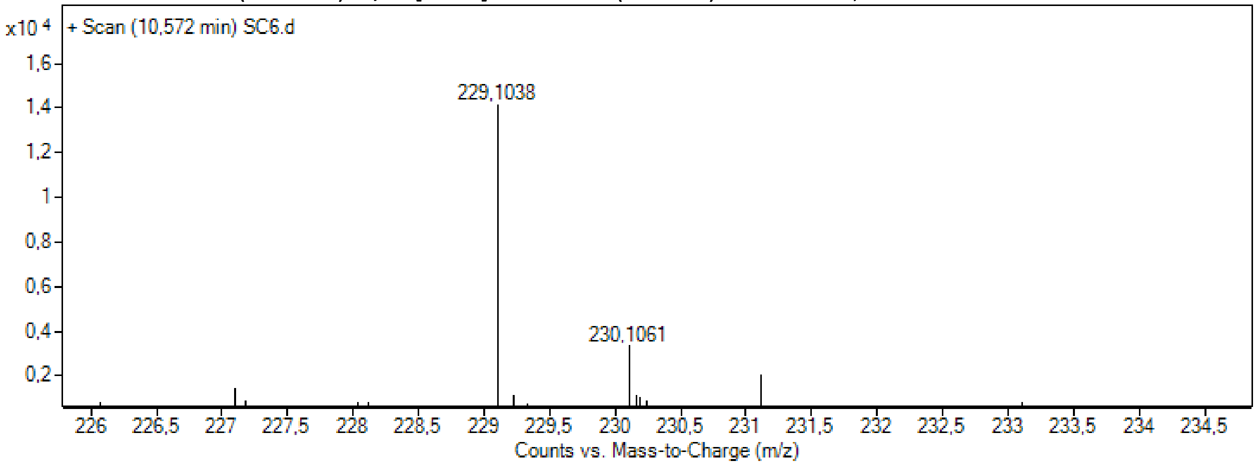

(2-(Hex-1-yn-1-yl)phenyl)(methyl)sulfane (**1f**)
HRMS (ESI-TOF) *m/z*: $[M+H]^+$ calcd for $(C_{13}H_{17}S)^+$: 205.1045; found: 210.1055

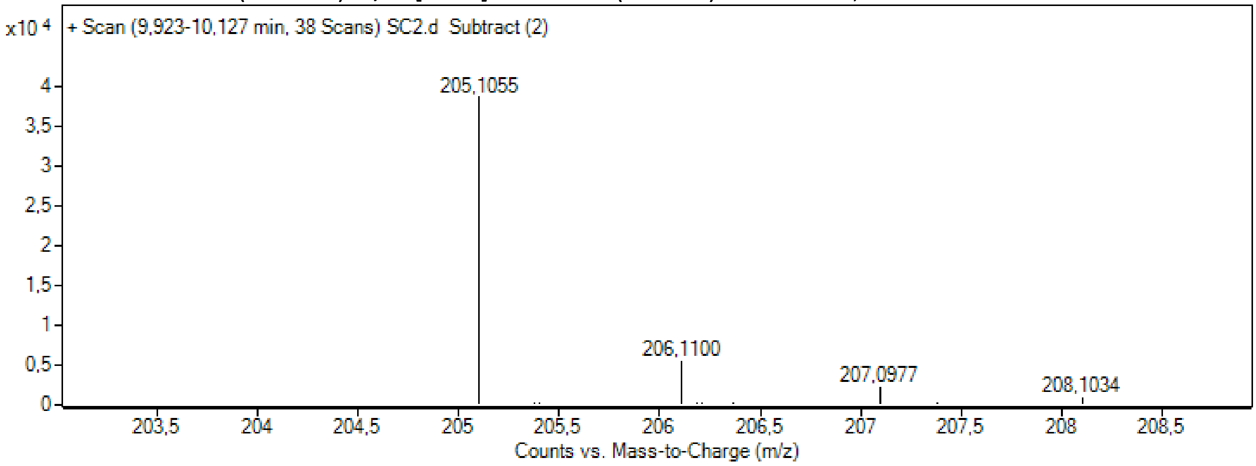

Methyl(2-(4-phenylbut-1-yn-1-yl)phenyl)sulfane (**1g**)

HRMS (ESI-TOF) *m/z*: $[M+H]^+$ calcd for $(C_{17}H_{17}S)^+$: 253.1045; found: 253.1066

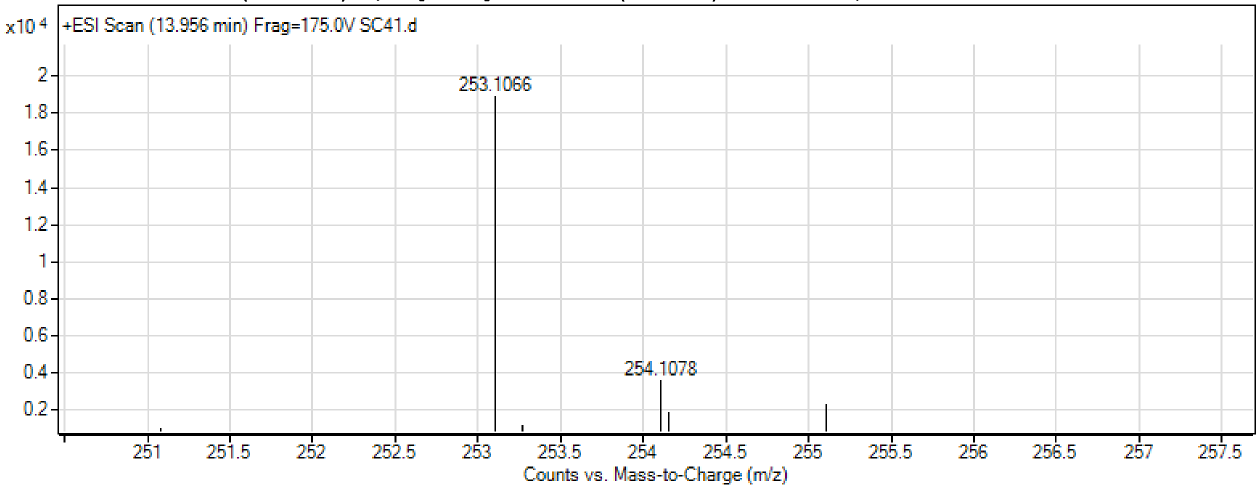

(2-(3,3-Dimethylbut-1-yn-1-yl)phenyl)(methyl)sulfane (**1h**)

HRMS (ESI-TOF) *m/z*: $[M+H]^+$ calcd for $(C_{13}H_{17}S)^+$: 205.1045; found: 205.1058

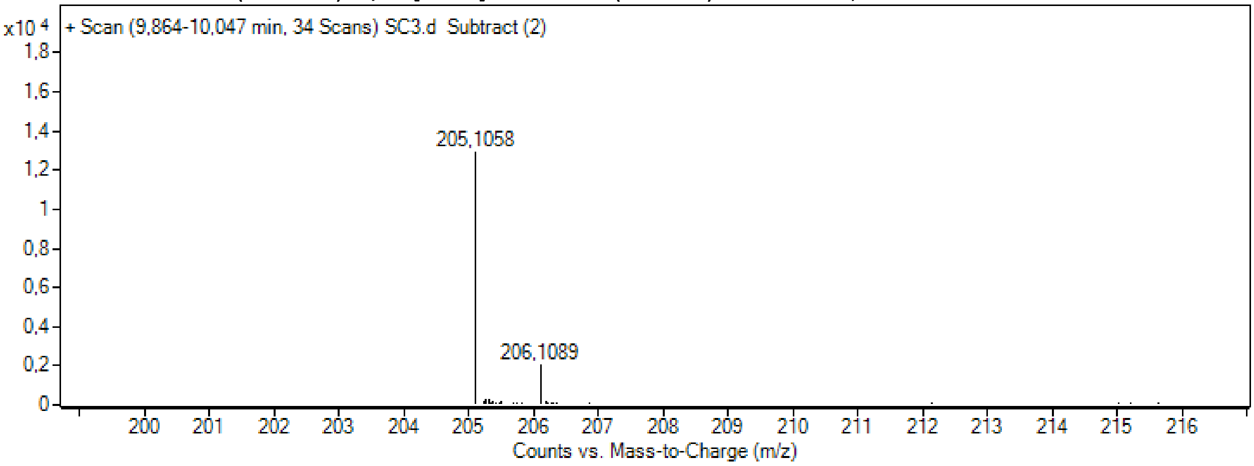

Methyl(4-methyl-2-(phenylethynyl)phenyl)sulfane (**1i**)

HRMS (ESI-TOF) *m/z*: $[M+H]^+$ calcd for $(C_{16}H_{15}S)^+$: 239.0889; found: 239.0894

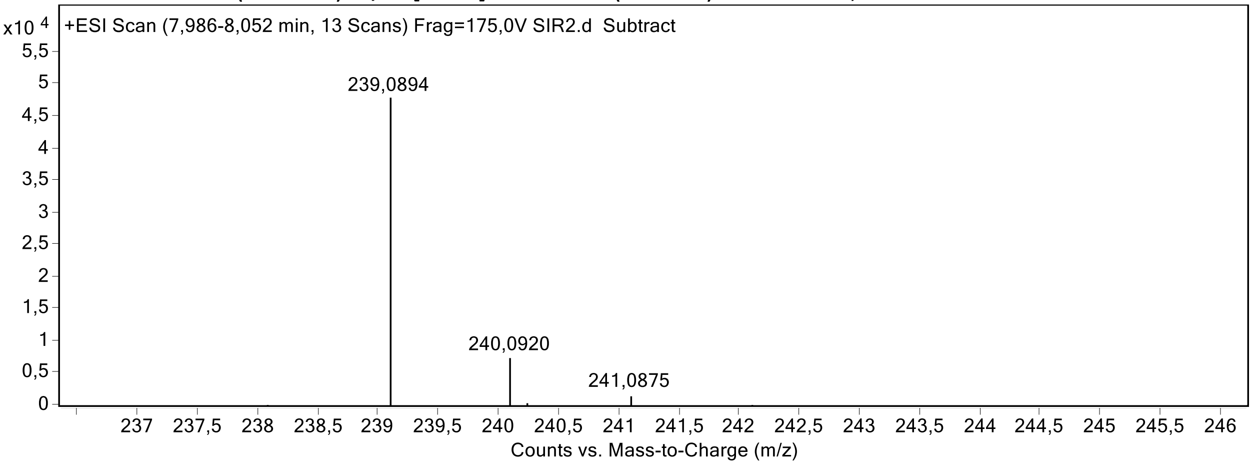

4-Fluoro-2-(phenylethynyl)phenyl(methyl)sulfane (**1j**)

HRMS (ESI-TOF) *m/z*: $[M+H]^+$ calcd for $(C_{15}H_{12}FS)^+$: 243.0638; found: 243.0644

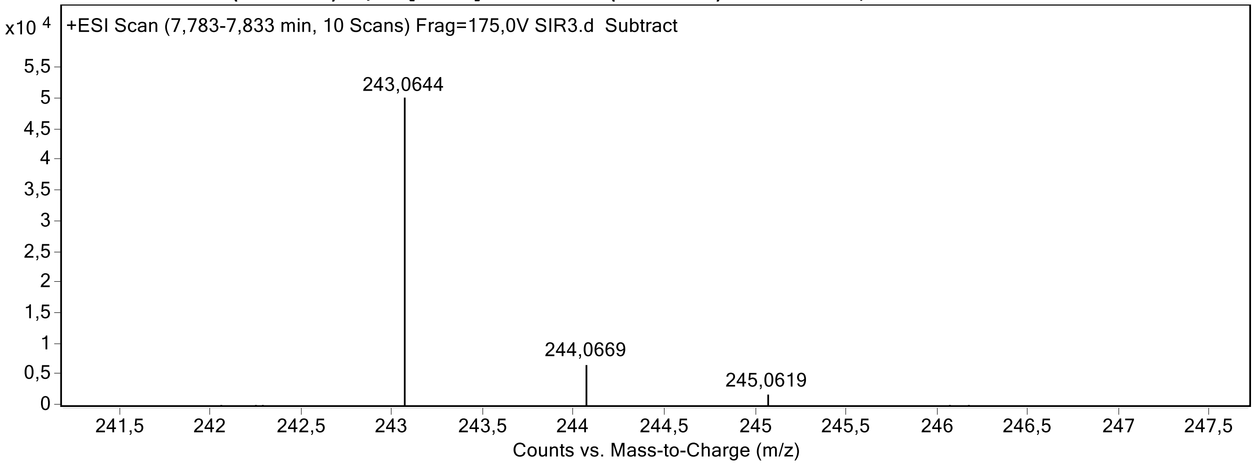

5-Fluoro-2-(phenylethynyl)phenyl(methyl)sulfane (**1k**)

HRMS (ESI-TOF) *m/z*: $[M+H]^+$ calcd for $(C_{15}H_{12}FS)^+$: 243.0638; found: 243.0641

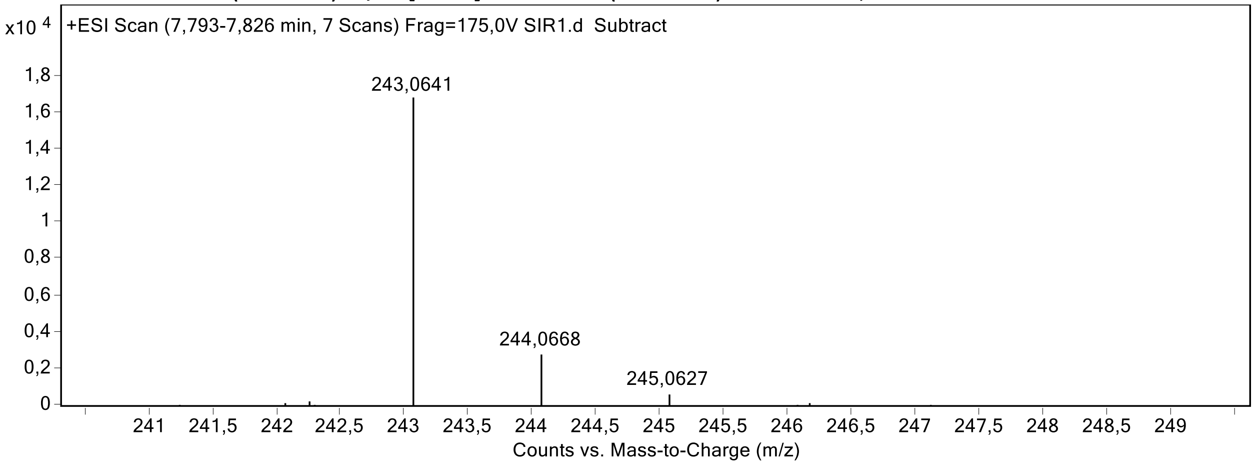

Methyl 2-phenylbenzo[*b*]thiophene-3-carboxylate (**2a**)

HRMS (ESI-TOF) *m/z*: $[M+H]^+$ calcd for $(C_{16}H_{13}O_2S)^+$: 269.0631; found: 269.0624

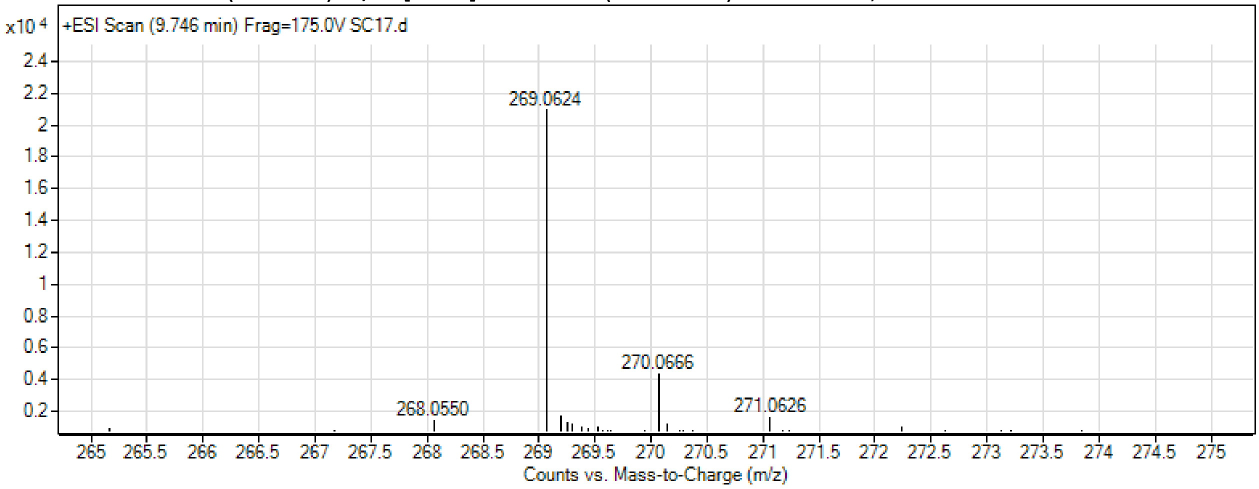

Ethyl 2-phenylbenzo[*b*]thiophene-3-carboxylate (**2a'**)
HRMS (ESI-TOF) *m/z*: $[M+H]^+$ calcd for $(C_{17}H_{15}O_2S)^+$: 283.0787; found: 283.0788

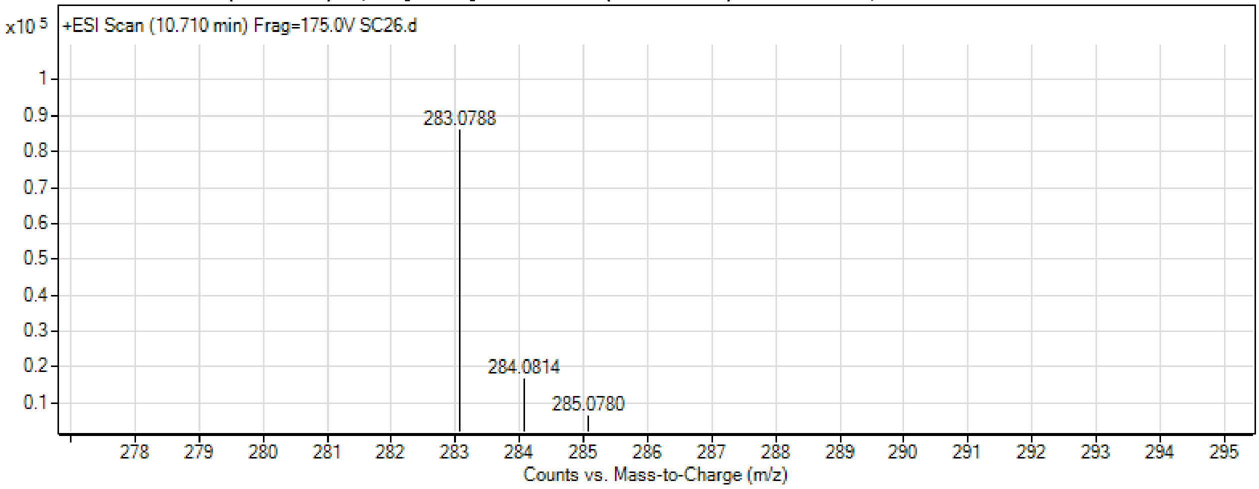

Isopropyl 2-phenylbenzo[*b*]thiophene-3-carboxylate (**2a''**)
HRMS (ESI-TOF) *m/z*: $[M+H]^+$ calcd for $(C_{18}H_{17}O_2S)^+$: 297.0944; found: 297.0943

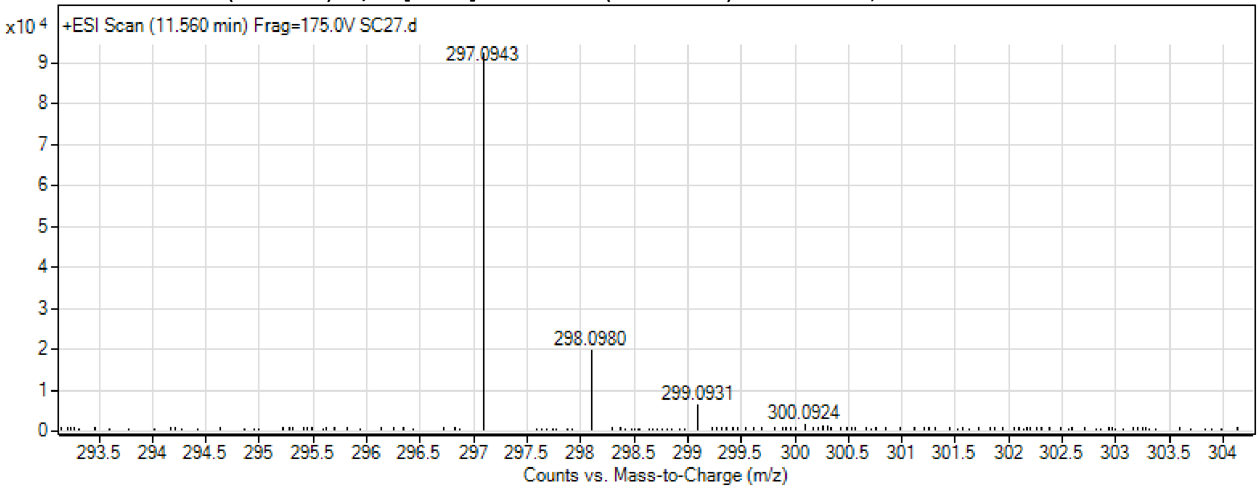

Methyl 2-(*p*-tolyl)benzo[*b*]thiophene-3-carboxylate (**2b**)
HRMS (ESI-TOF) *m/z*: $[M+H]^+$ calcd for $(C_{17}H_{15}O_2S)^+$: 283.0787; found: 283.0785

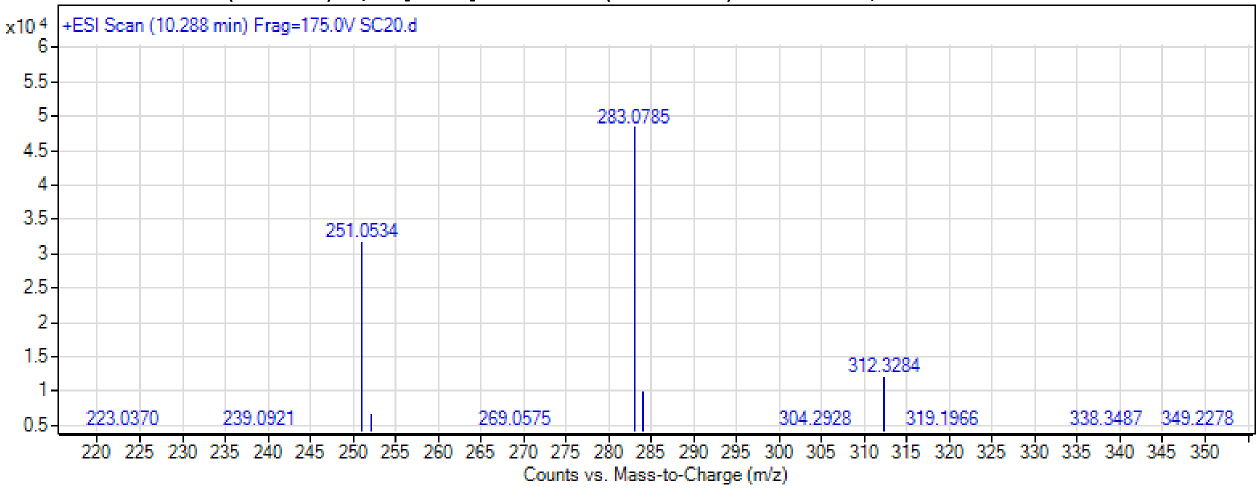

Methyl 2-(thiophen-3-yl)benzo[*b*]thiophene-3-carboxylate (**2d**)

HRMS (ESI-TOF) *m/z*: $[M+H]^+$ calcd for $(C_{14}H_{11}O_2S_2)^+$: 275.0195; found: 275.0194

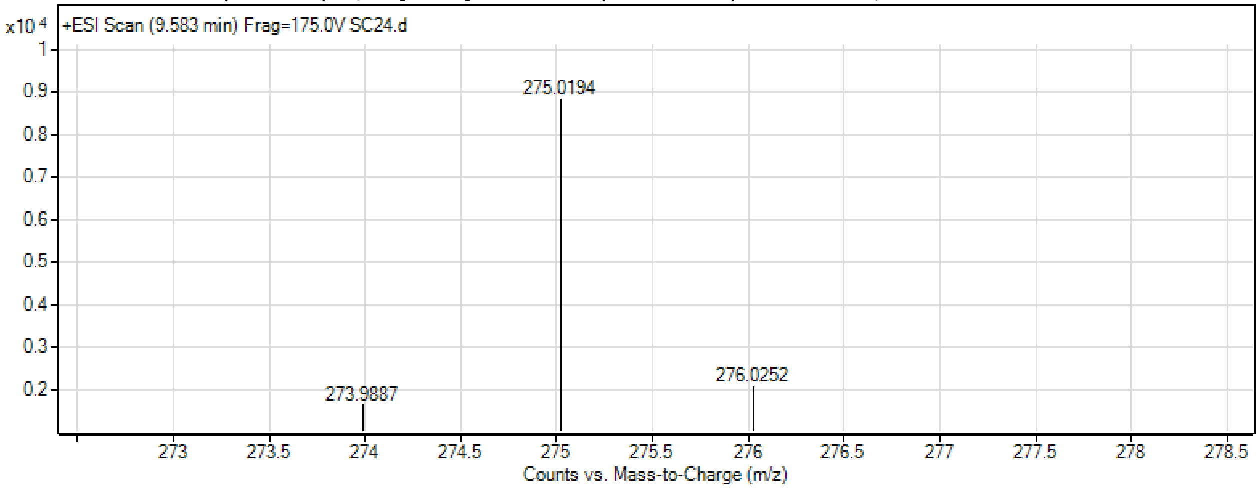

Methyl 2-(cyclohex-1-en-1-yl)benzo[*b*]thiophene-3-carboxylate (**2e**)

HRMS (ESI-TOF) *m/z*: $[M+H]^+$ calcd for $(C_{16}H_{17}O_2S)^+$: 273.0944; found: 273.0952

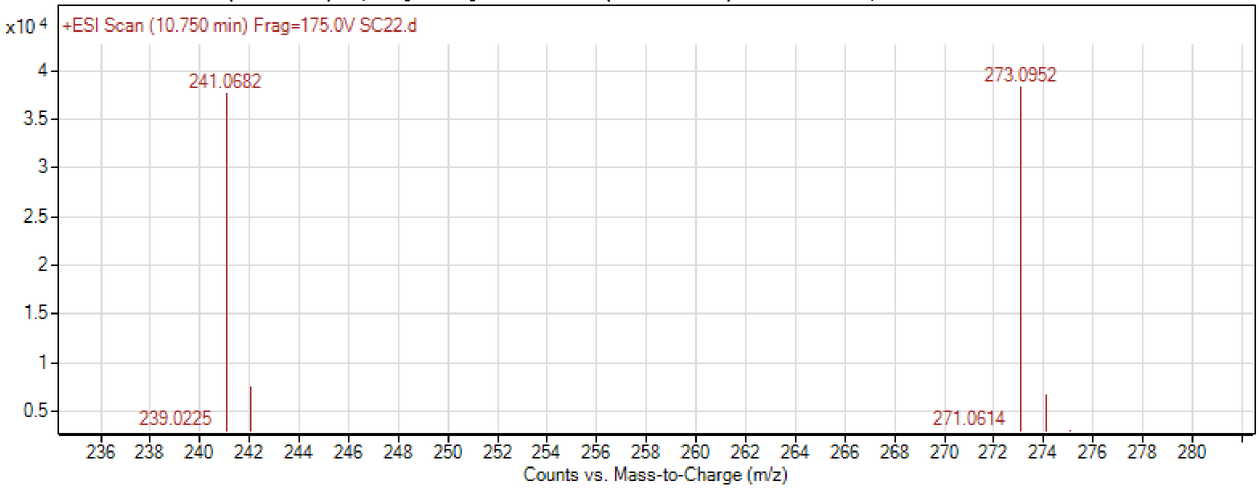

Methyl 2-butylbenzo[*b*]thiophene-3-carboxylate (**2f**)

HRMS (ESI-TOF) *m/z*: $[M+H]^+$ calcd for $(C_{14}H_{17}O_2S)^+$: 249.0944; found: 249.0963

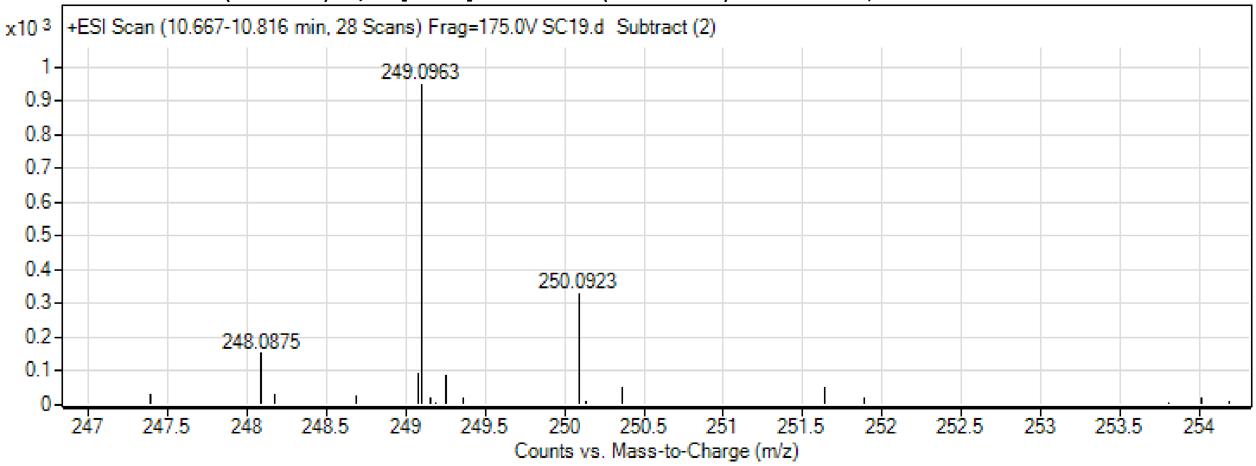

Methyl 2-(*tert*-butyl)benzo[*b*]thiophene-3-carboxylate (**2h**)

HRMS (ESI-TOF) *m/z*: [M+H]$^+$ calcd for ($C_{14}H_{17}O_2S$)$^+$: 249.0944; found: 249.0957

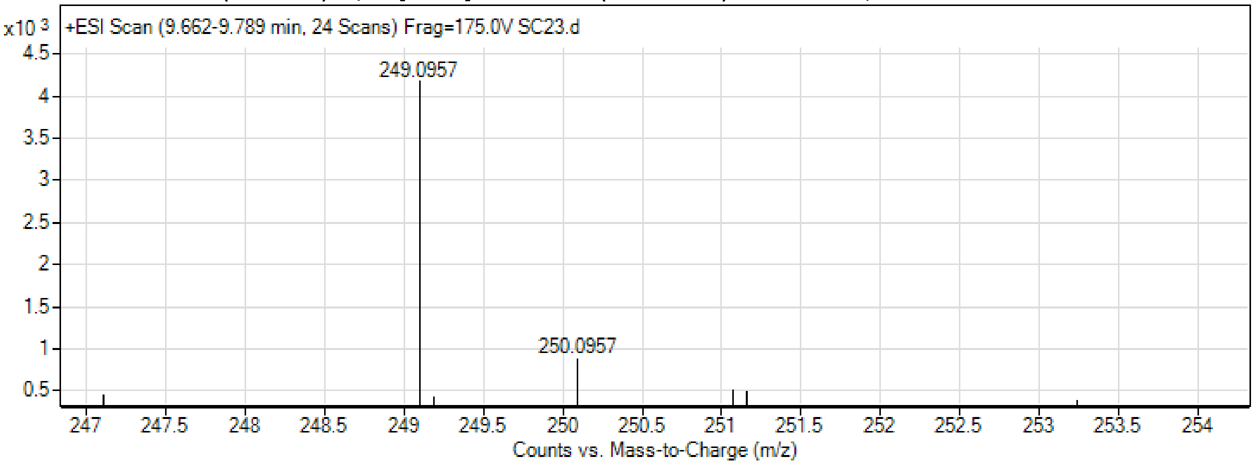

Methyl 5-methyl-2-phenylbenzo[*b*]thiophene-3-carboxylate (**2i**)

HRMS (ESI-TOF) *m/z*: [M+H]$^+$ calcd for ($C_{17}H_{15}O_2S$)$^+$: 283.0787; found: 283.0792

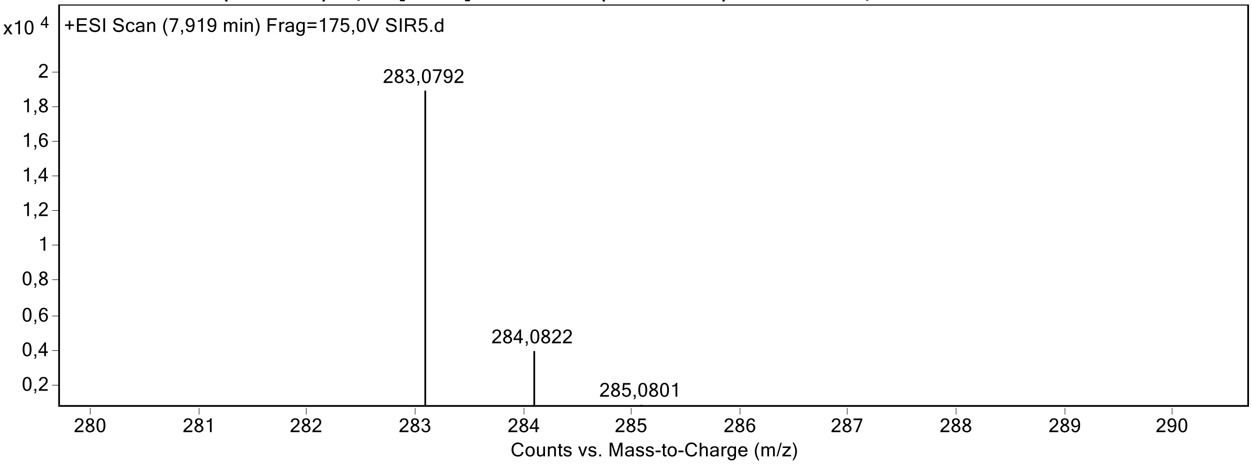

Methyl 5-fluoro-2-phenylbenzo[*b*]thiophene-3-carboxylate ate (**2j**)

HRMS (ESI-TOF) *m/z*: [M+H]$^+$ calcd for ($C_{16}H_{12}FO_2S$)$^+$: 287.0537; found: 287.0540

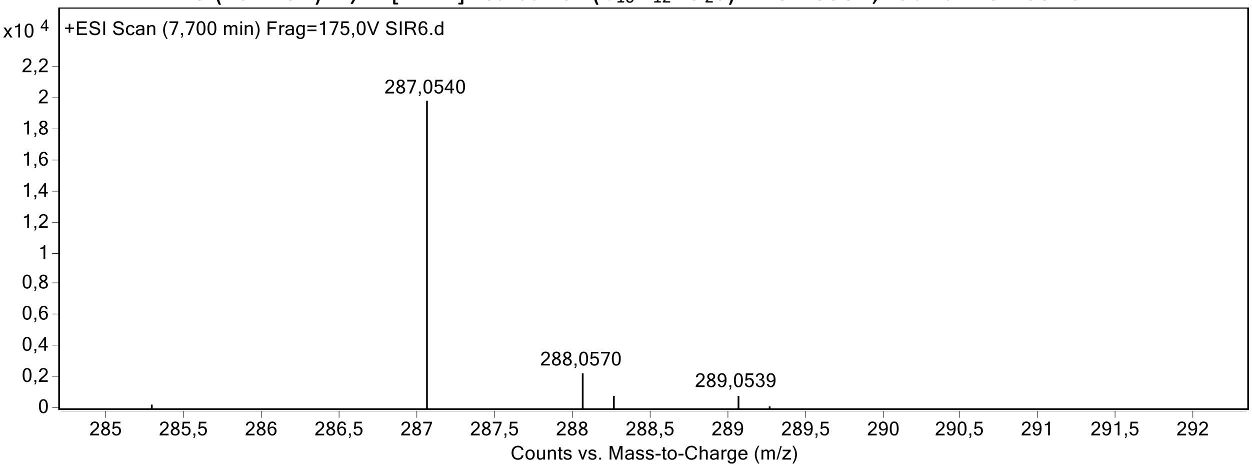

Methyl 6-fluoro-2-phenylbenzo[*b*]thiophene-3-carboxylate ate (**2k**)
HRMS (ESI-TOF) *m/z*: $[M+H]^+$ calcd for $(C_{16}H_{12}FO_2S)^+$: 287.0537; found: 287.0537

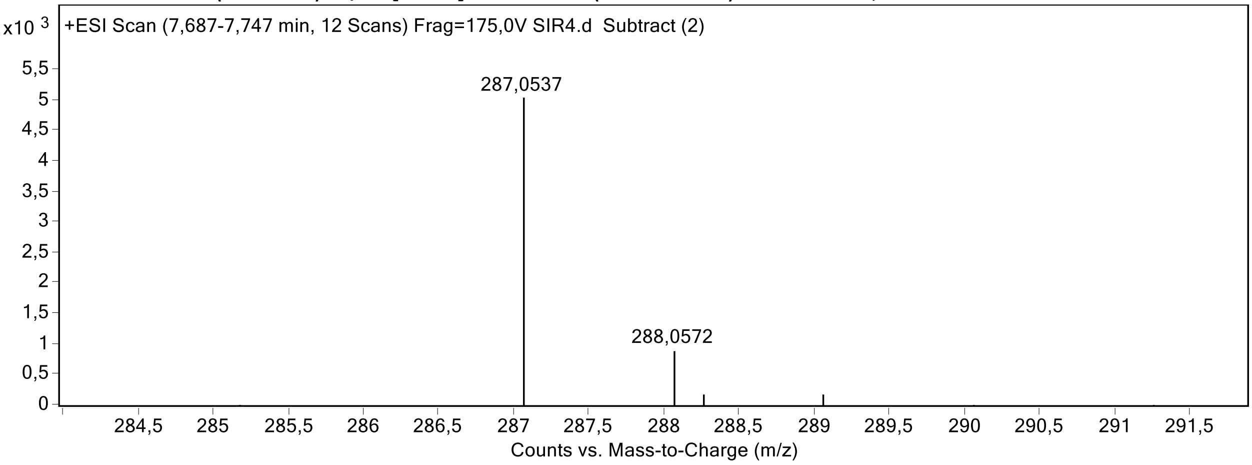

Copies of ^{1}H and ^{13}C{^{1}H}NMR Spectra

Methyl(2-(phenylethynyl)phenyl)sulfane (**1a**)

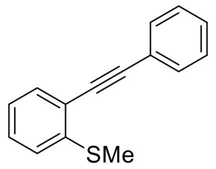

^{1}H NMR ($CDCl_3$, 300 MHz)

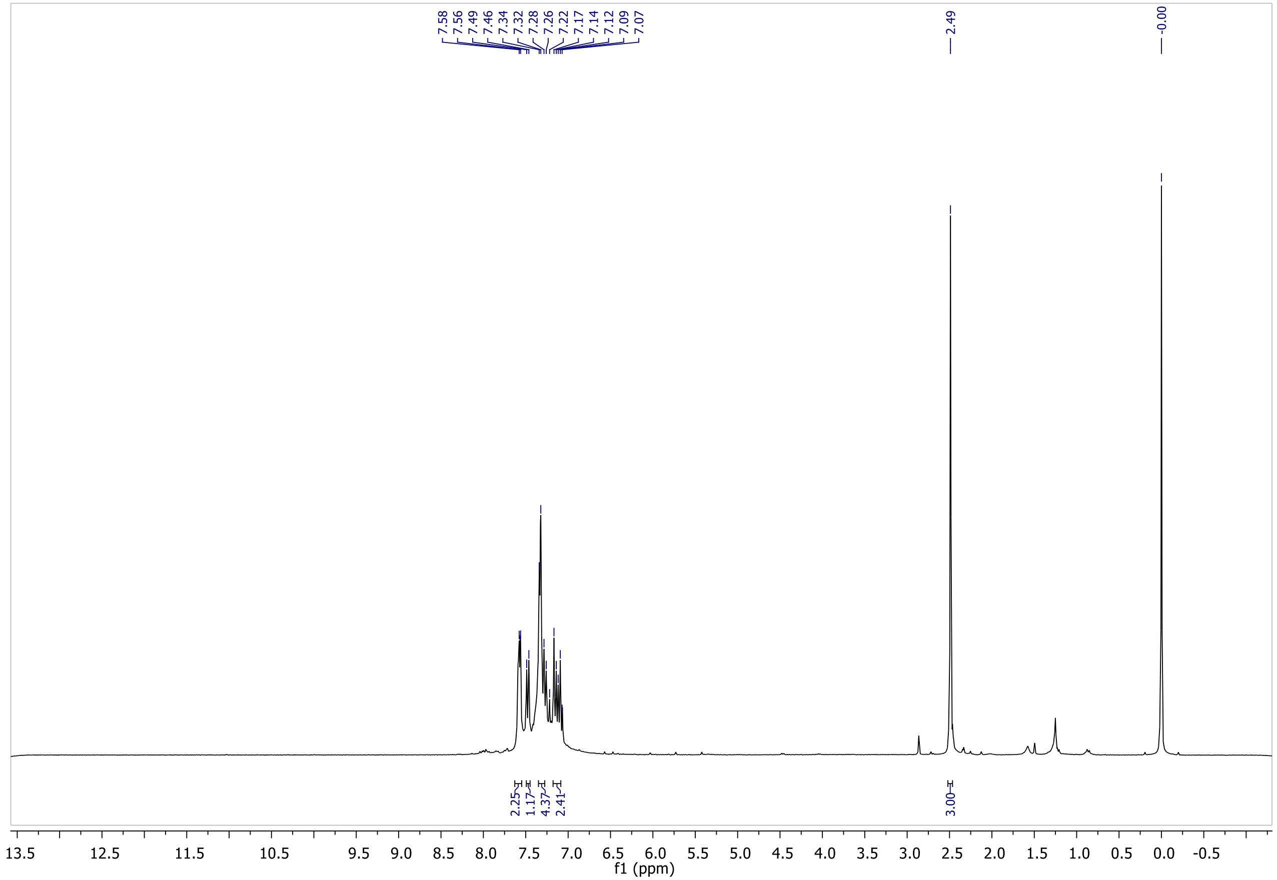

Methyl(2-(phenylethynyl)phenyl)sulfane (**1a**)

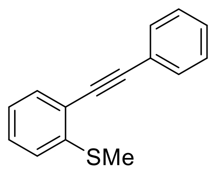

$^{13}C\{^1H\}$NMR ($CDCl_3$, 75 MHz)

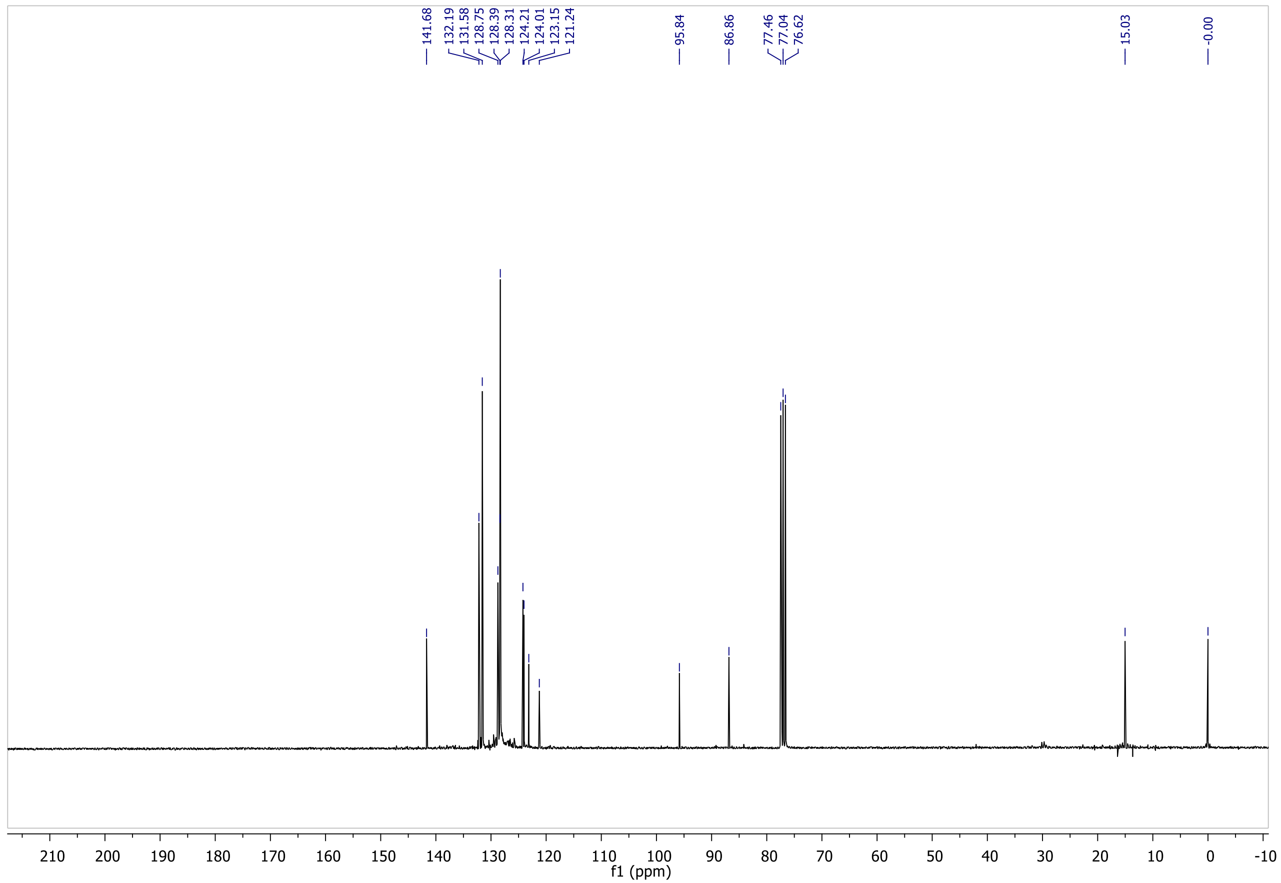

Methyl(2-(*p*-tolylethynyl)phenyl)sulfane (**1b**)

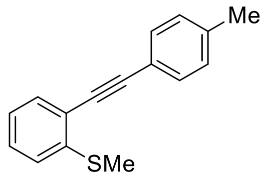

^{1}H NMR ($CDCl_3$, 300 MHz)

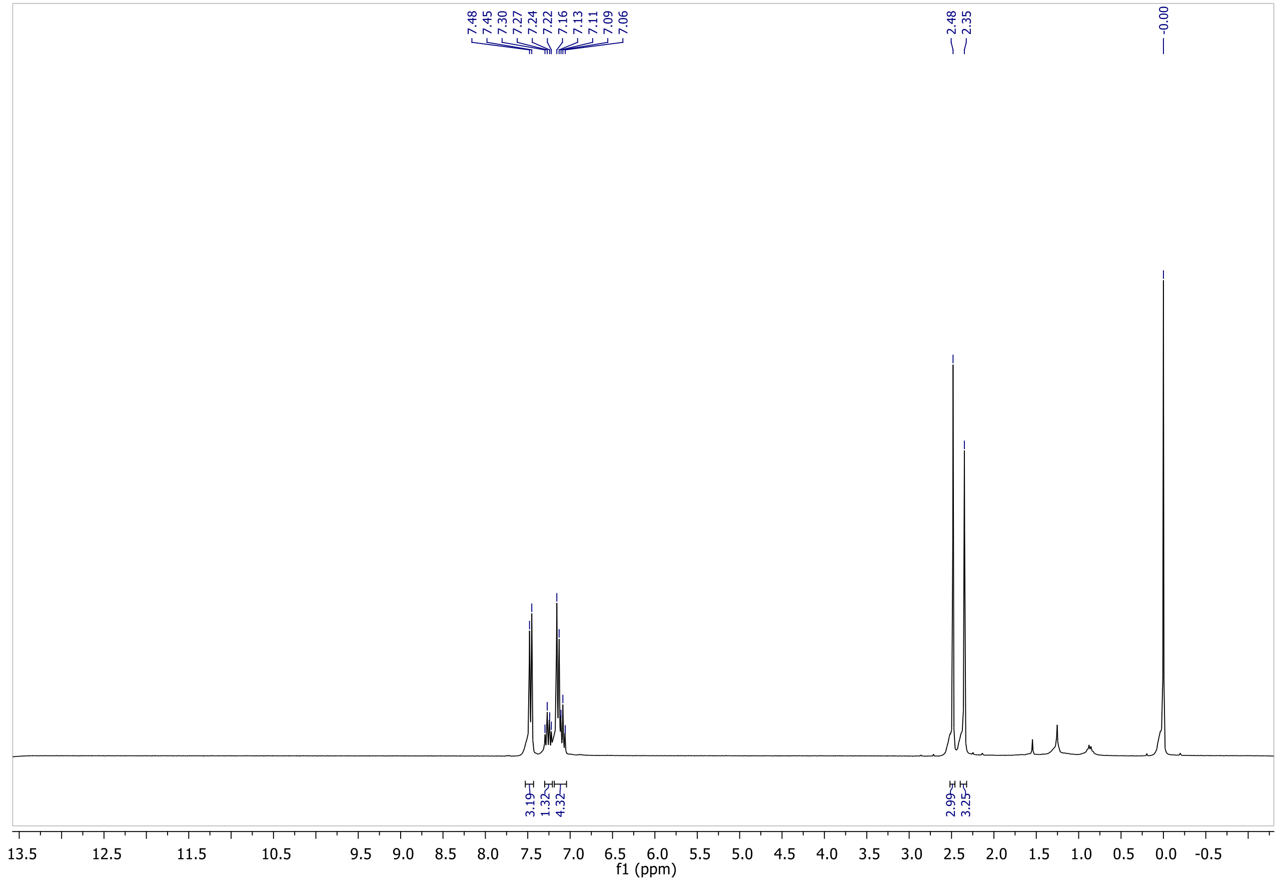

Methyl(2-(*p*-tolylethynyl)phenyl)sulfane (**1b**)

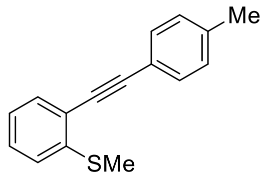

$^{13}C\{^{1}H\}$NMR ($CDCl_3$, 75 MHz)

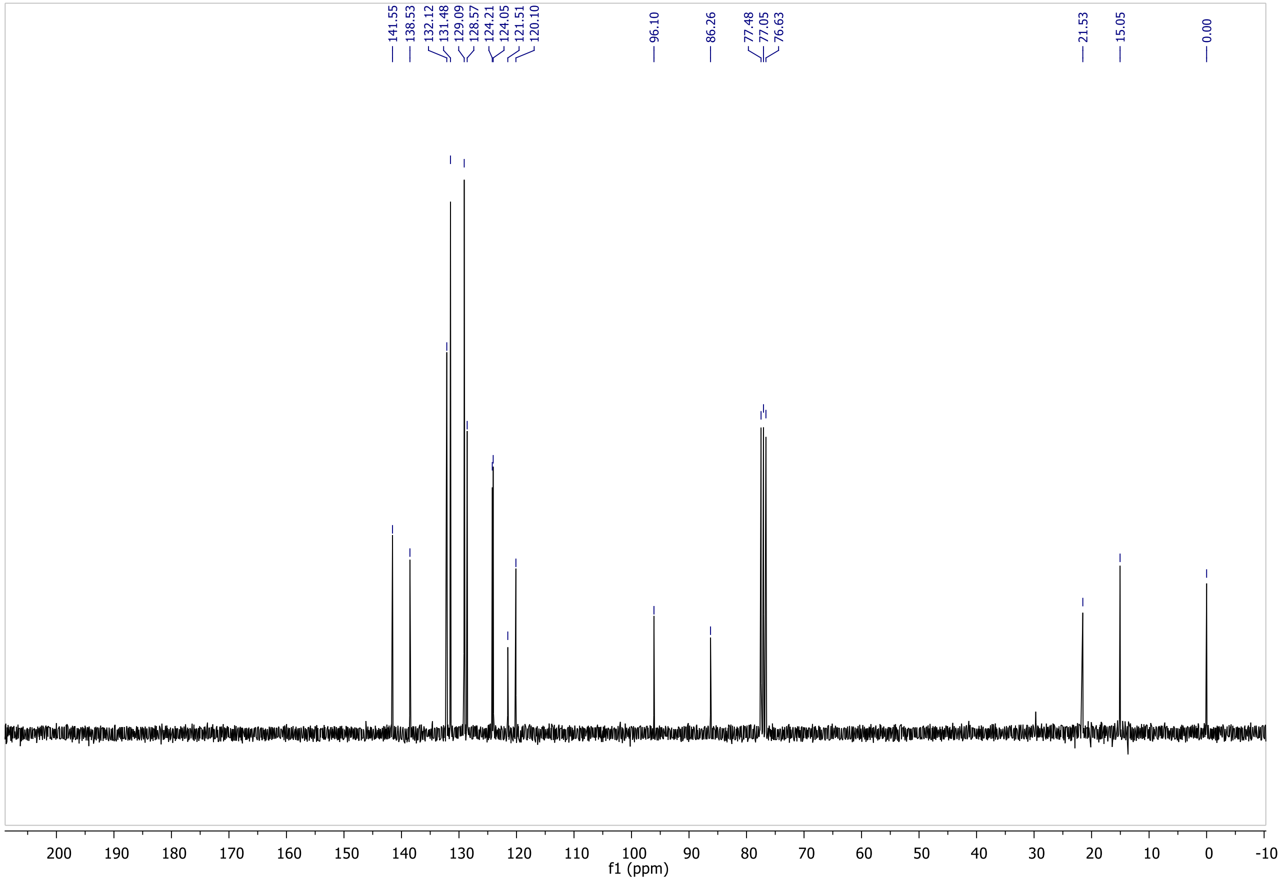

(2-((4-Bromophenyl)ethynyl)phenyl)(methyl)sulfane (**1c**)

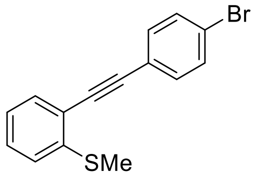

^{1}H NMR ($CDCl_3$, 300 MHz)

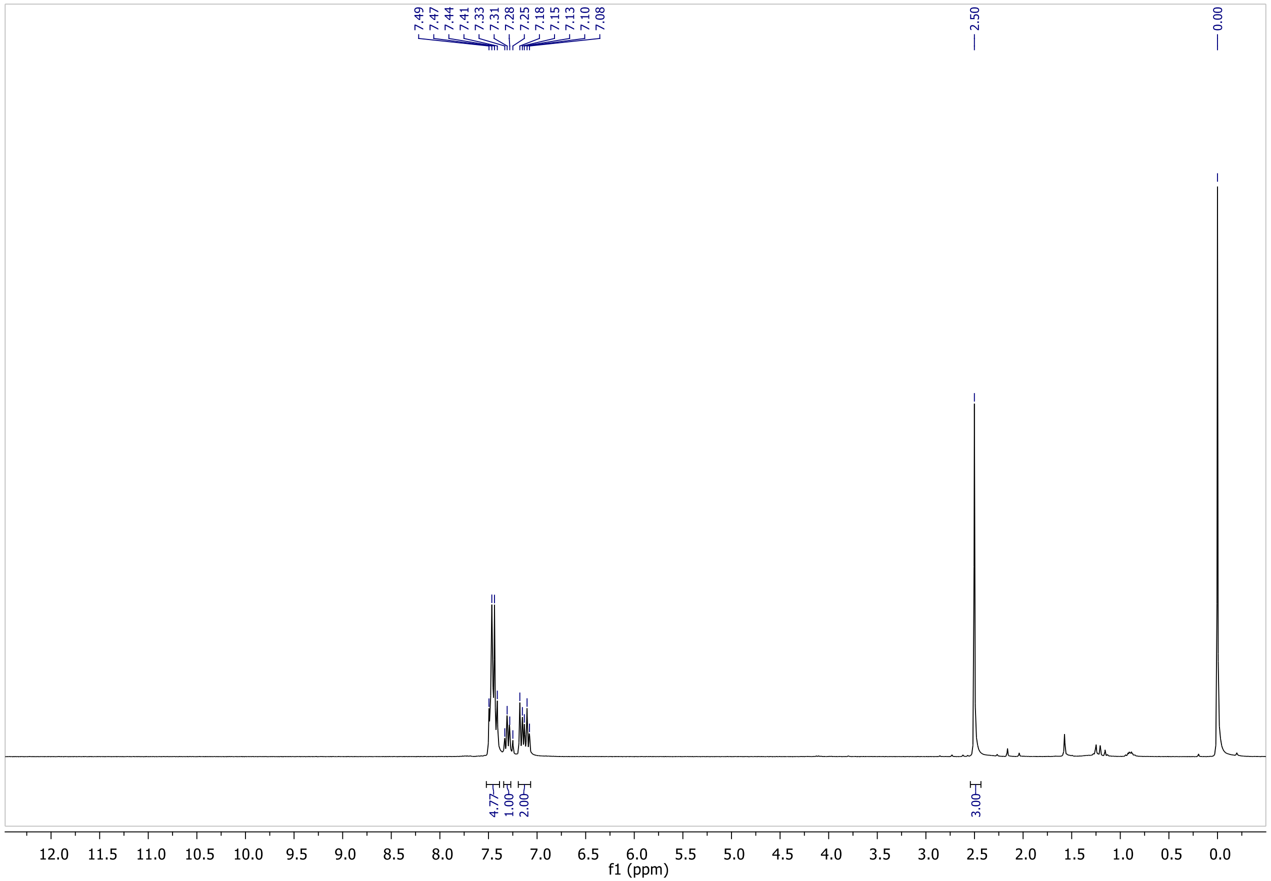

(2-((4-Bromophenyl)ethynyl)phenyl)(methyl)sulfane (**1c**)

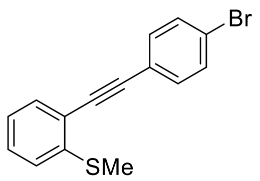

$^{13}C\{^{1}H\}$NMR ($CDCl_3$, 75 MHz)

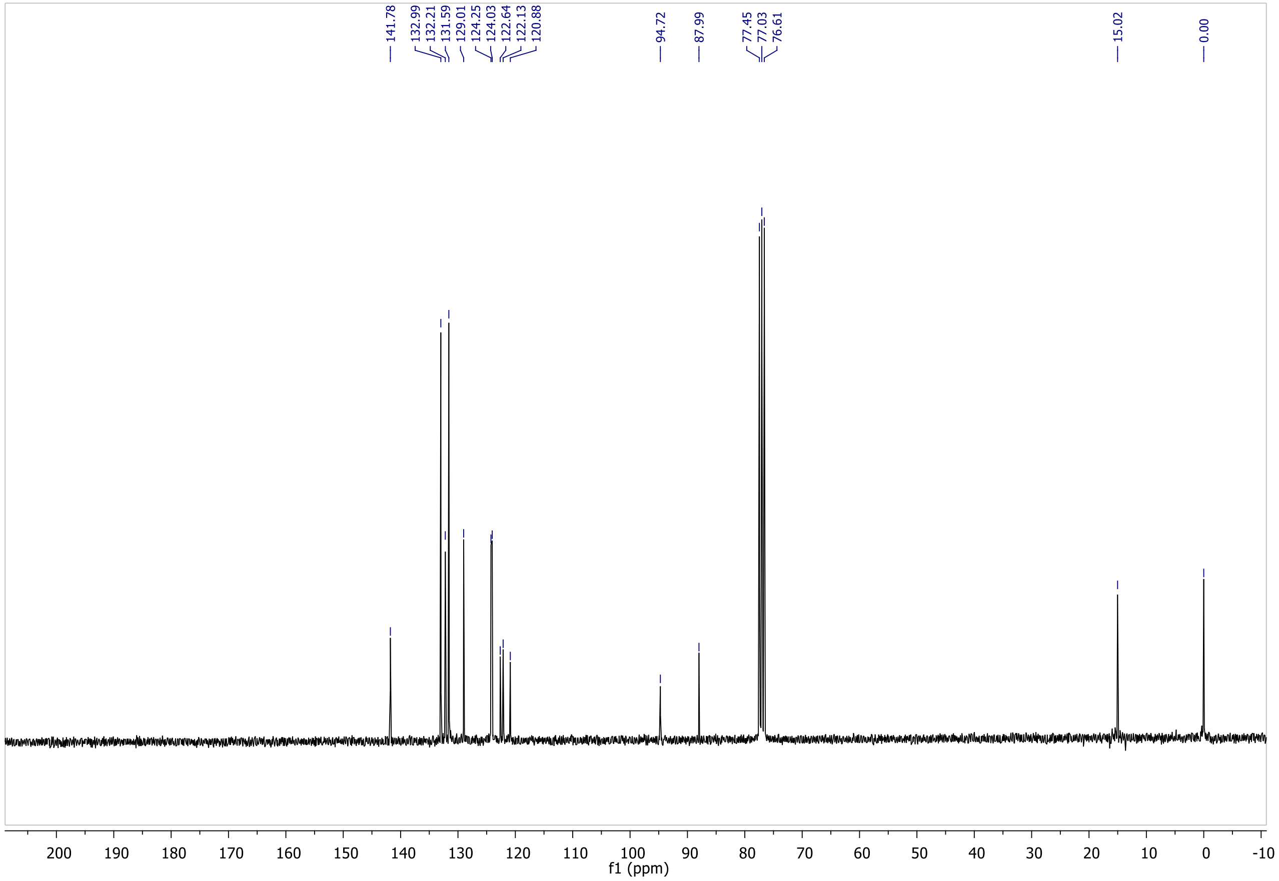

3-((2-(Methylthio)phenyl)ethynyl)thiophen*e* (**1d**)

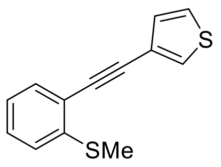

^{1}H NMR ($CDCl_3$, 300 MHz)

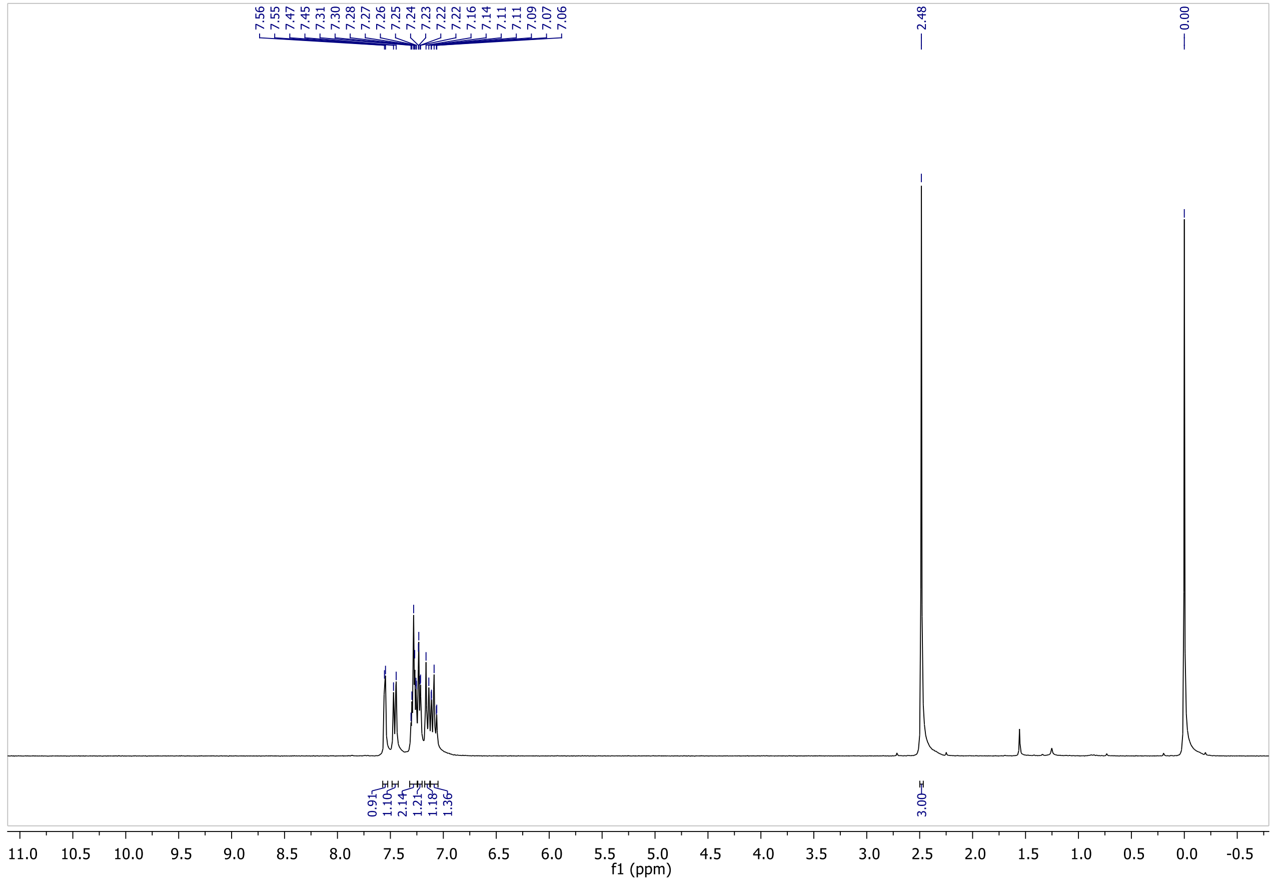

3-((2-(Methylthio)phenyl)ethynyl)thiophen*e* (**1d**)

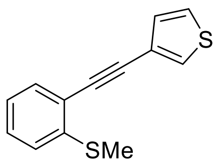

$^{13}C\{^1H\}$NMR ($CDCl_3$, 75 MHz)

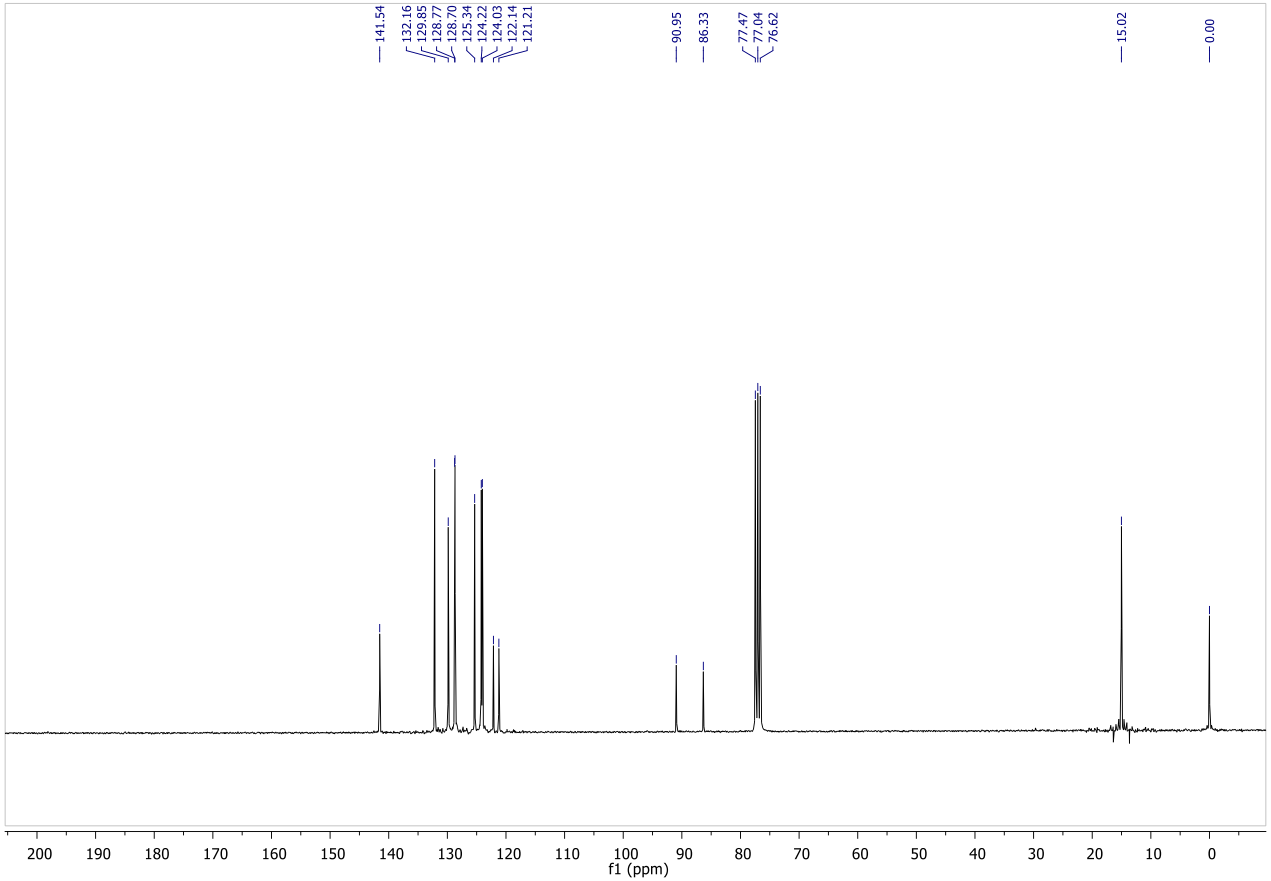

(2-(Cyclohex-1-en-1-ylethynyl)phenyl)(methyl)sulfane (**1e**)

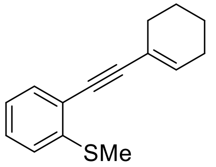

^{1}H NMR ($CDCl_3$, 300 MHz)

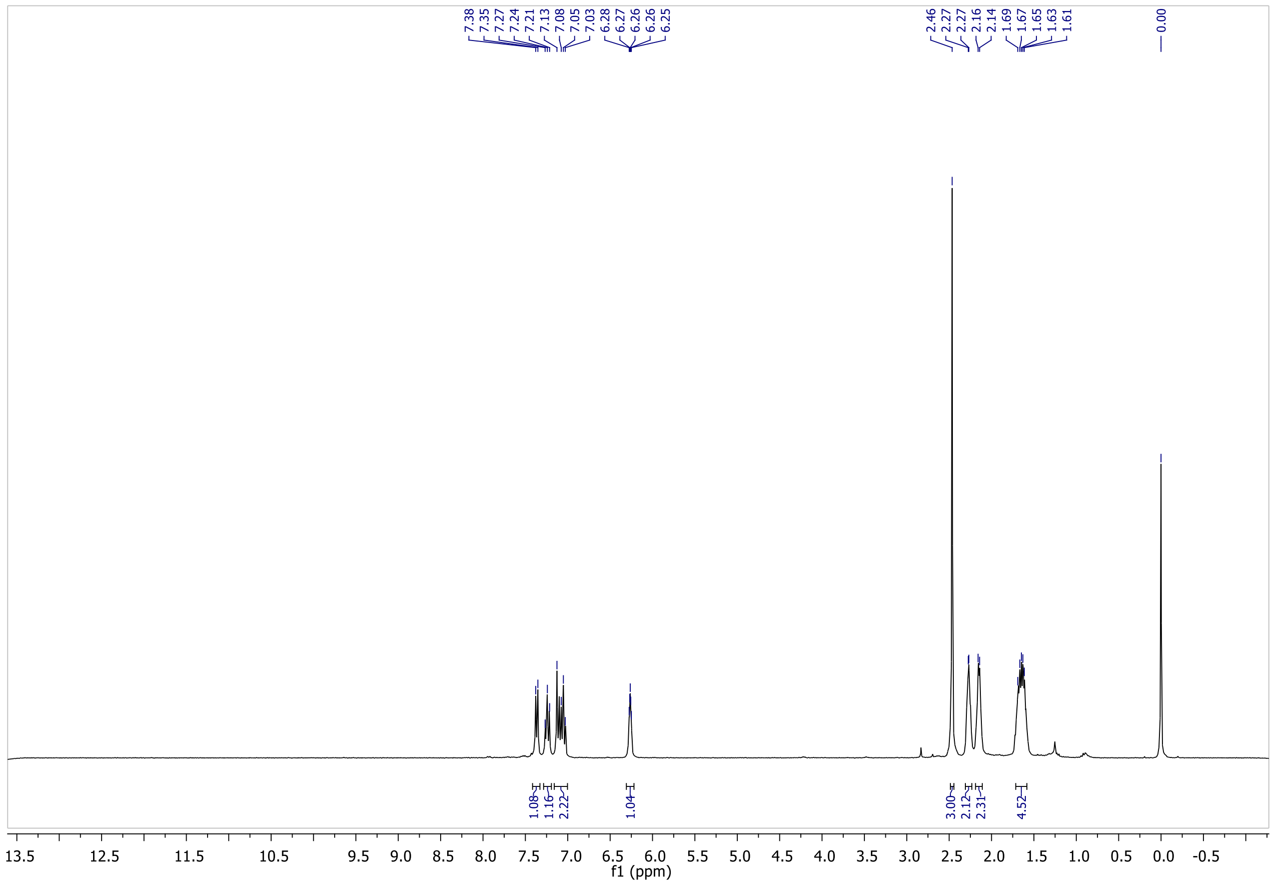

(2-(Cyclohex-1-en-1-ylethynyl)phenyl)(methyl)sulfane (**1e**)

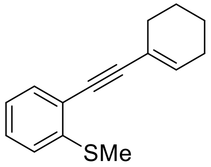

$^{13}C\{^1H\}$NMR ($CDCl_3$, 75 MHz)

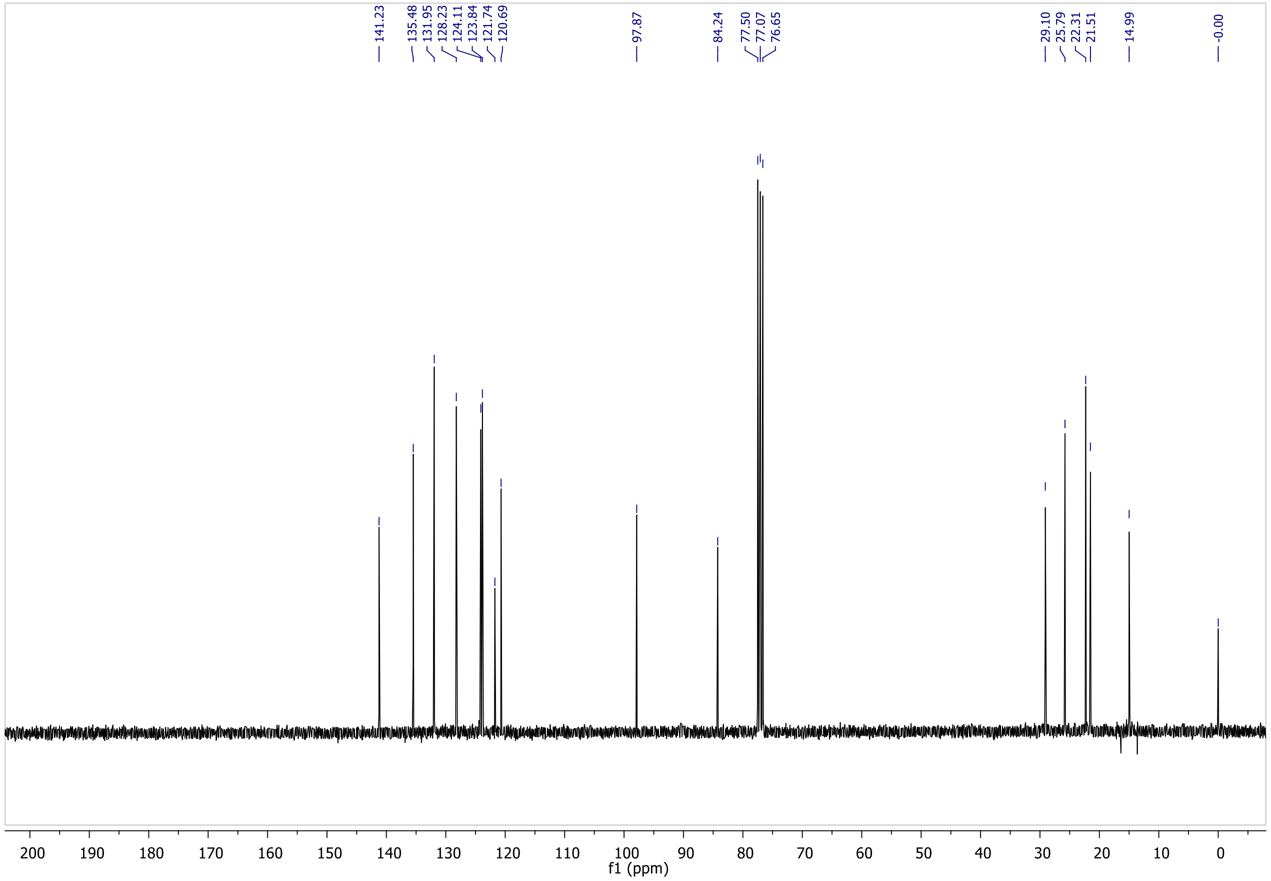

(2-(Hex-1-yn-1-yl)phenyl)(methyl)sulfane (**1f**)

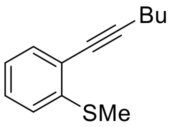

^{1}H NMR ($CDCl_3$, 300 MHz)

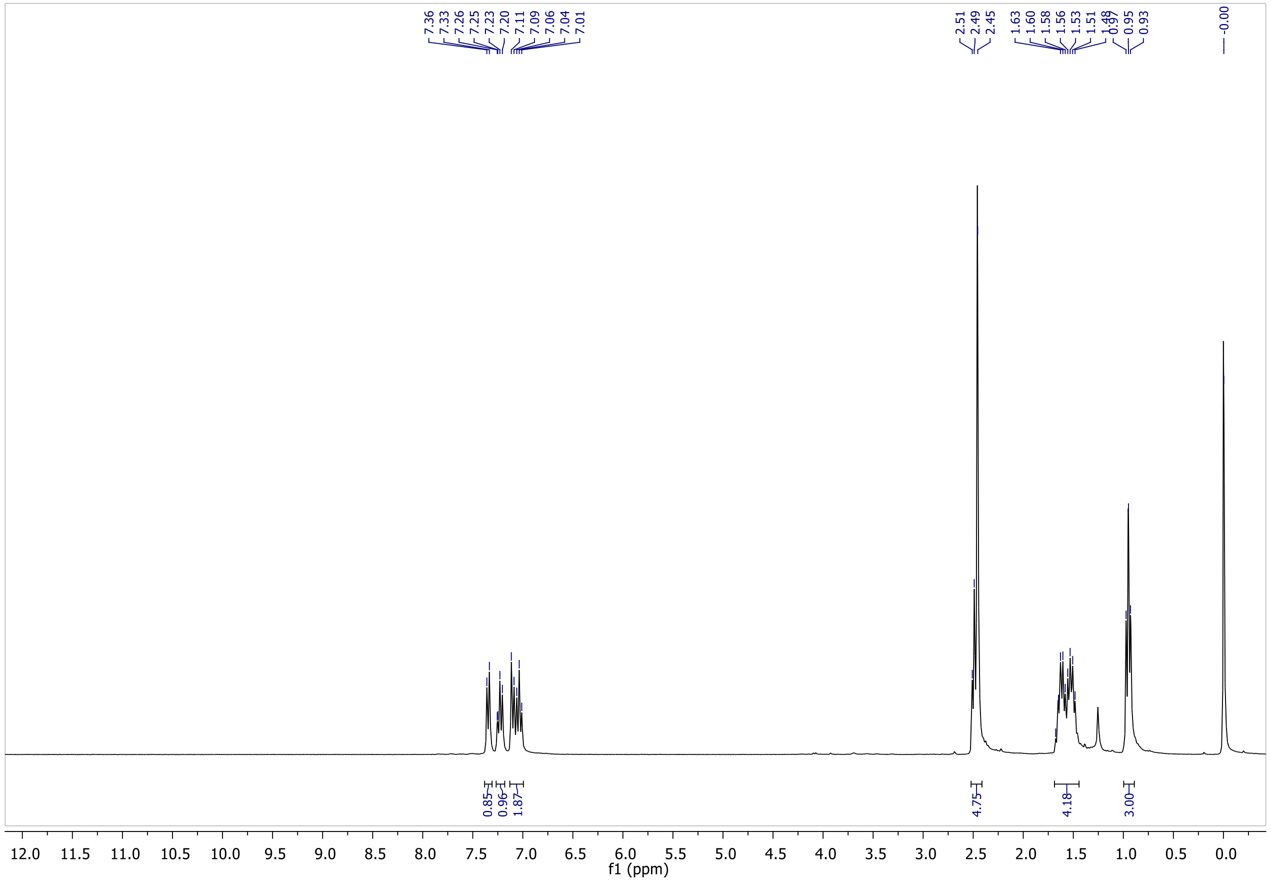

(2-(Hex-1-yn-1-yl)phenyl)(methyl)sulfane (**1f**)

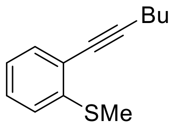

$^{13}C\{^{1}H\}$NMR ($CDCl_3$, 75 MHz)

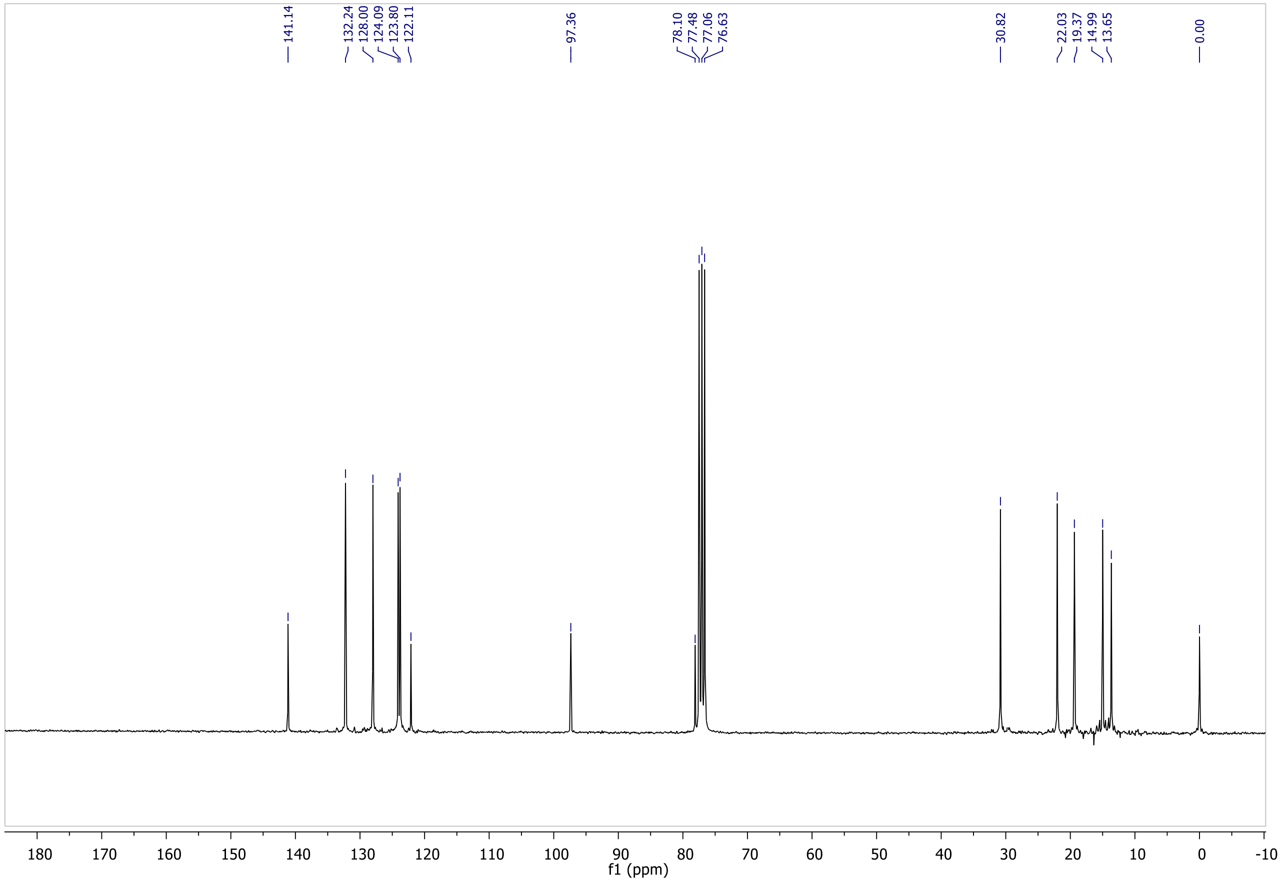

Methyl(2-(4-phenylbut-1-yn-1-yl)phenyl)sulfane (**1g**)

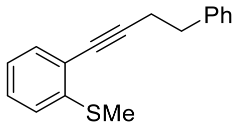

^{1}H NMR ($CDCl_3$, 500 MHz)

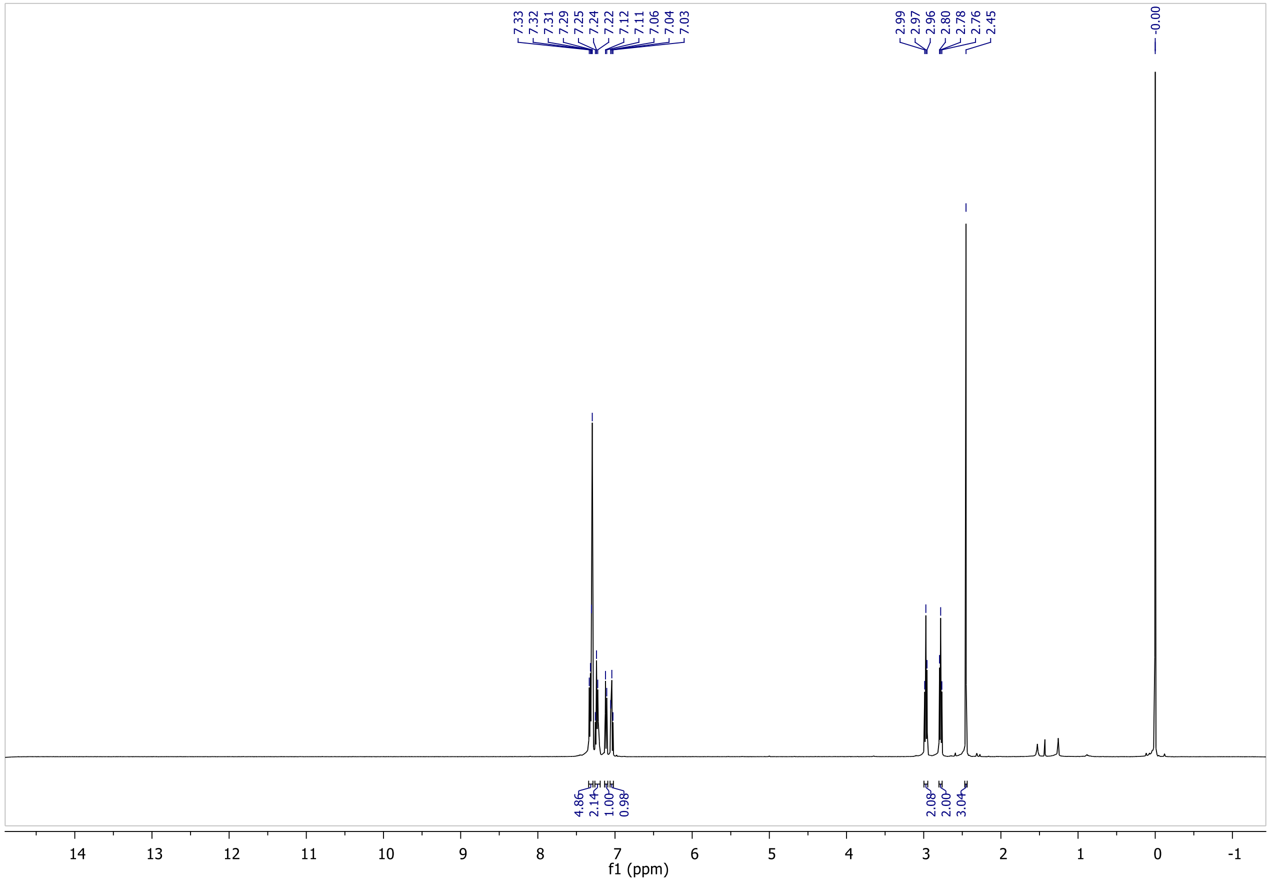

Methyl(2-(4-phenylbut-1-yn-1-yl)phenyl)sulfane (**1g**)

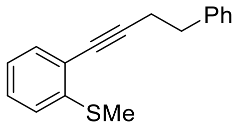

$^{13}C\{^{1}H\}$NMR ($CDCl_3$, 125 MHz)

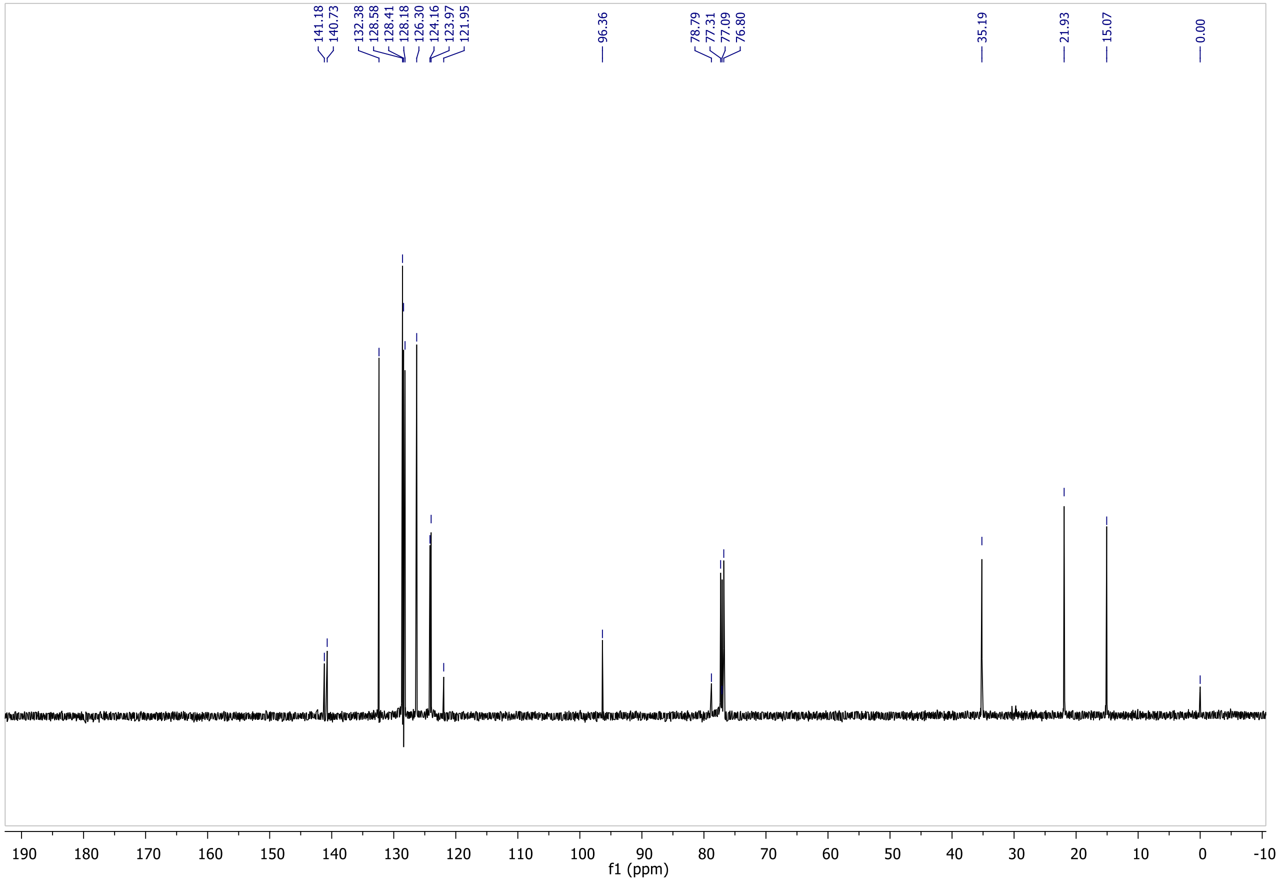

(2-(3,3-Dimethylbut-1-yn-1-yl)phenyl)(methyl)sulfane (**1h**)

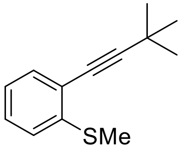

^{1}H NMR ($CDCl_3$, 500 MHz)

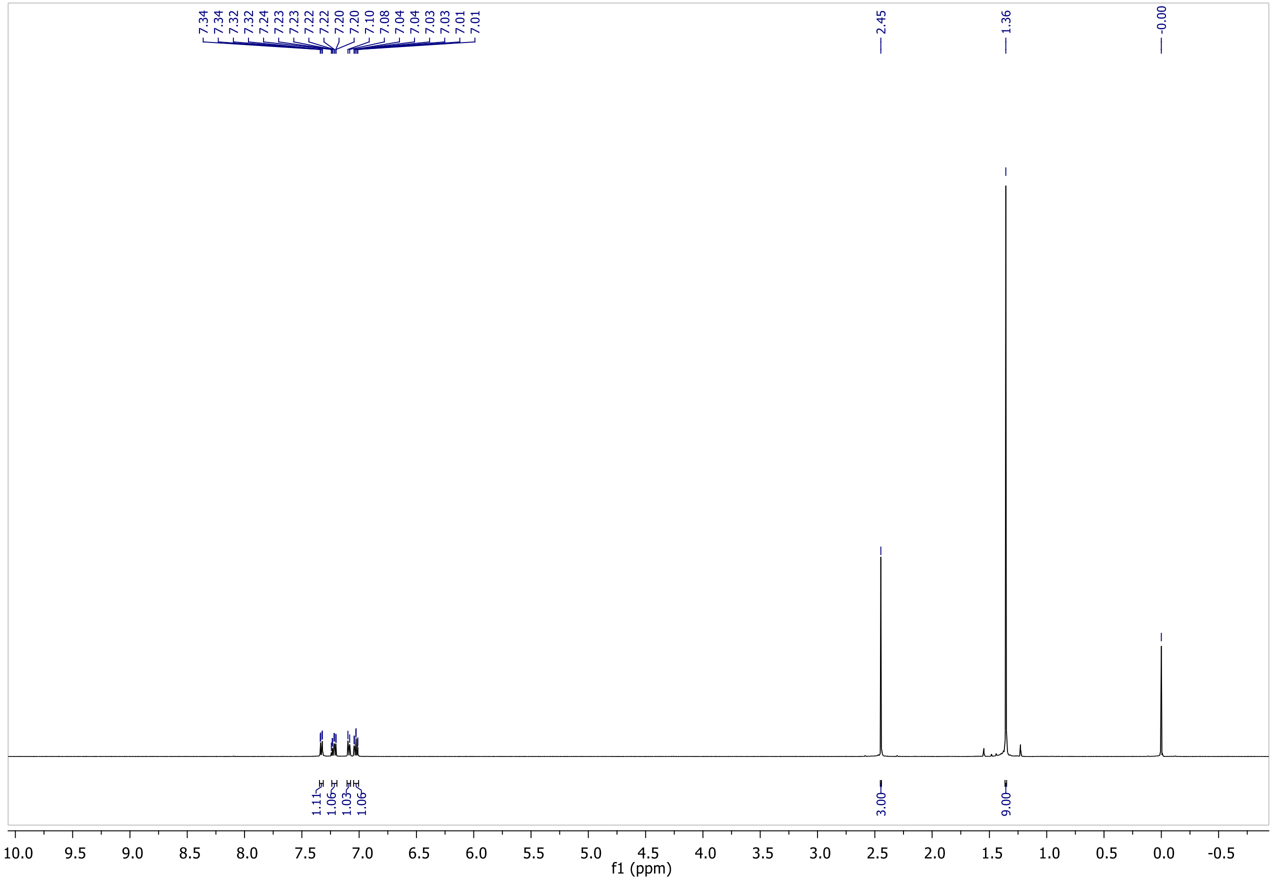

(2-(3,3-Dimethylbut-1-yn-1-yl)phenyl)(methyl)sulfane (**1h**)

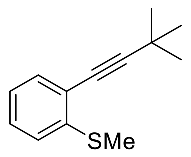

$^{13}C\{^{1}H\}$NMR ($CDCl_3$, 125 MHz)

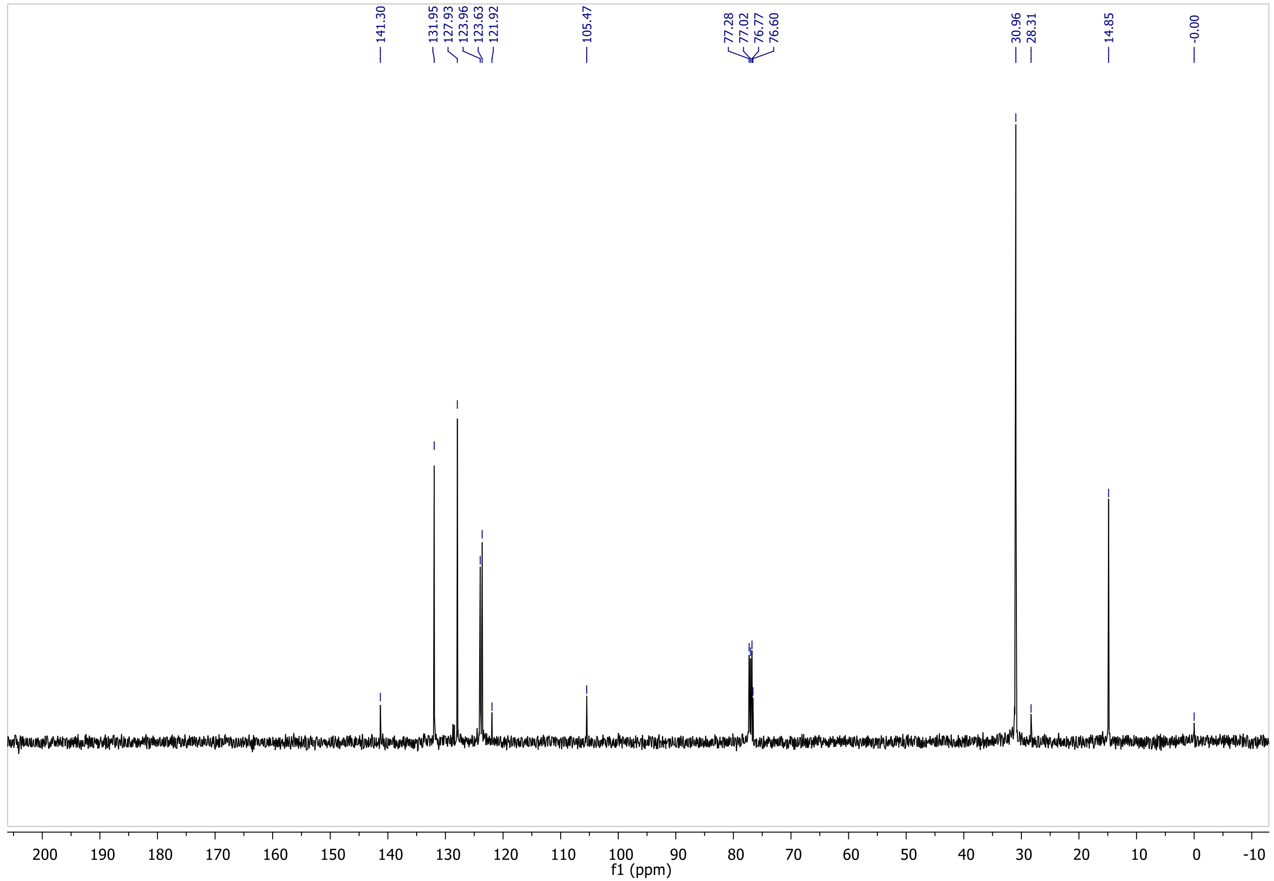

Methyl(4-methyl-2-(phenylethynyl)phenyl)sulfane (**1i**)

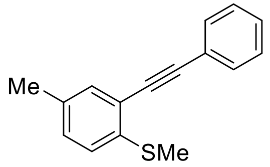

^{1}H NMR ($CDCl_3$, 500 MHz)

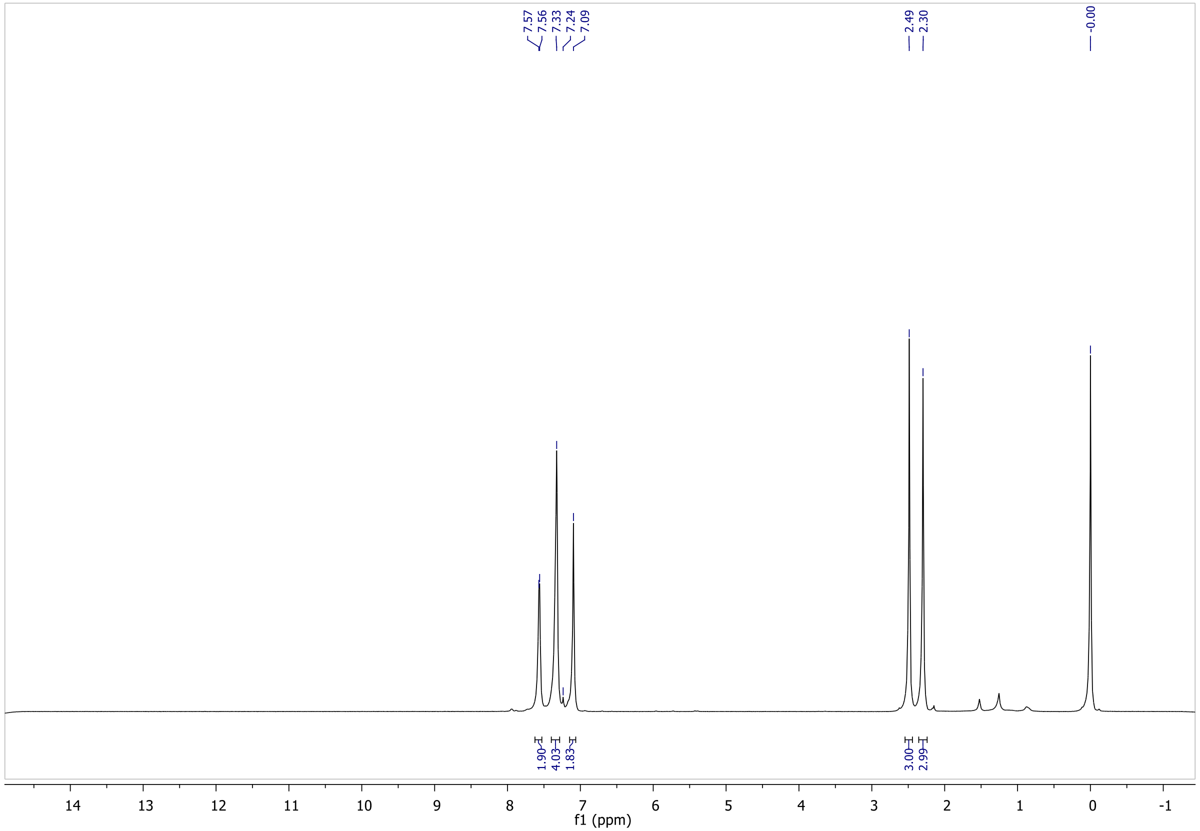

Methyl(4-methyl-2-(phenylethynyl)phenyl)sulfane (**1i**)

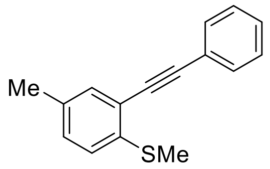

$^{13}C\{^1H\}$NMR ($CDCl_3$, 125 MHz)

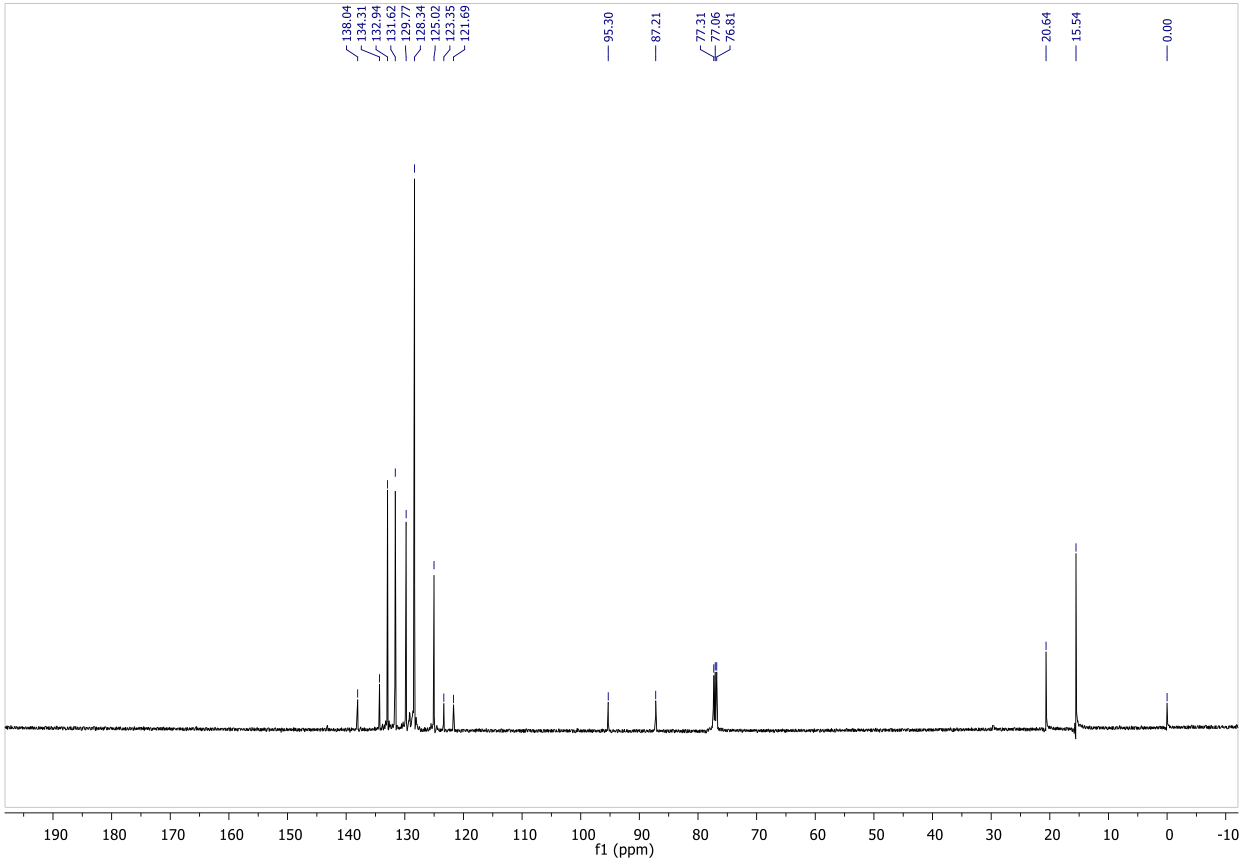

4-Fluoro-2-(phenylethynyl)phenyl(methyl)sulfane (**1j**)

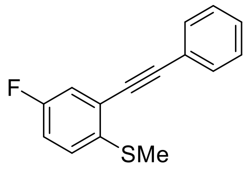

^{1}H NMR ($CDCl_3$, 500 MHz)

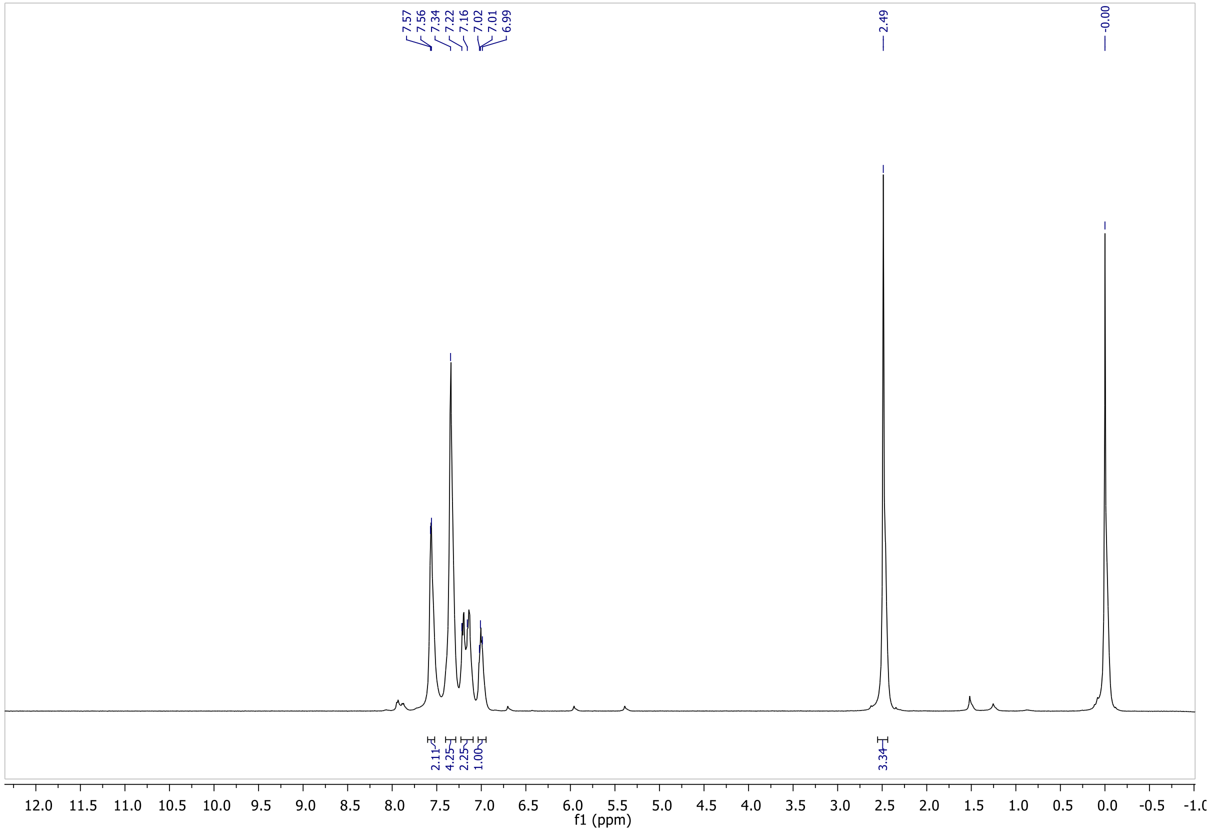

4-Fluoro-2-(phenylethynyl)phenyl(methyl)sulfane (**1j**)

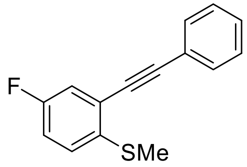

$^{13}C\{^{1}H\}$NMR ($CDCl_3$, 125 MHz)

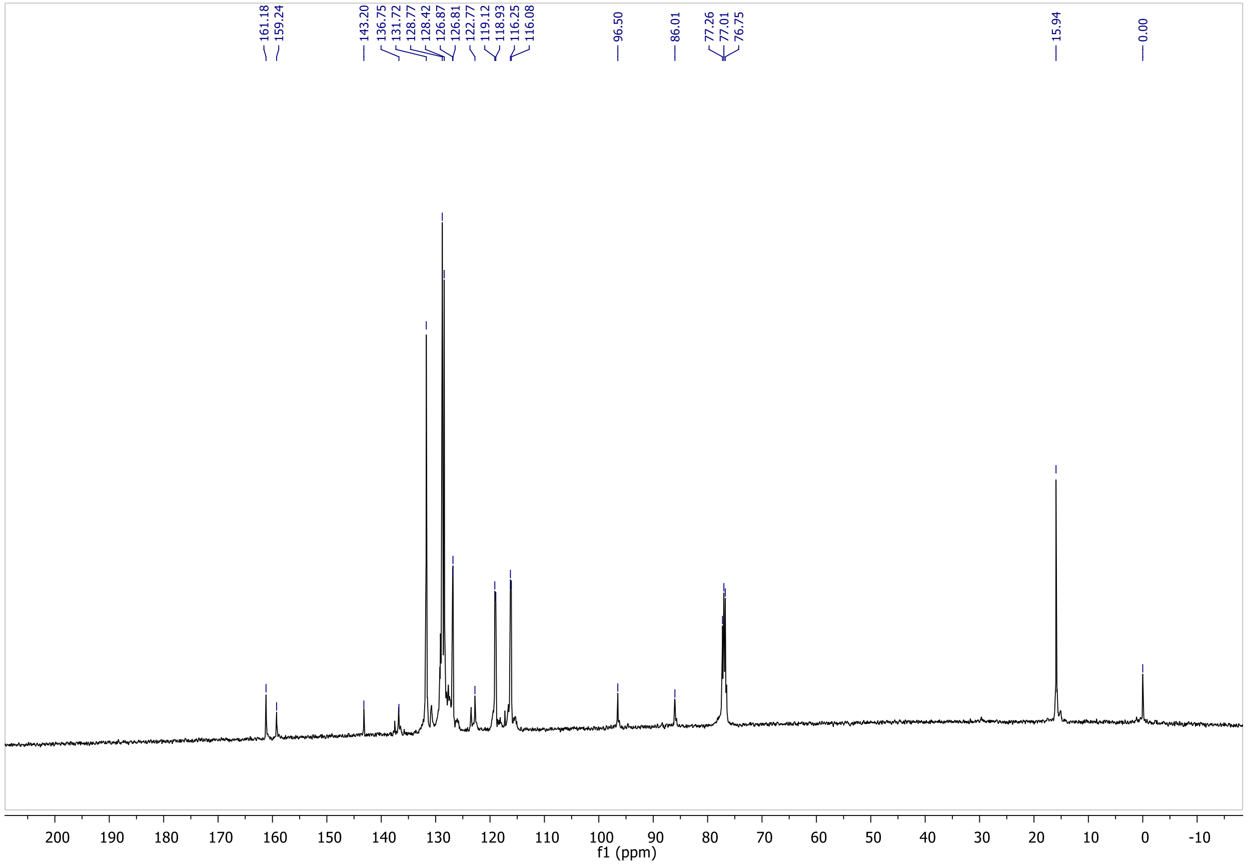

5-Fluoro-2-(phenylethynyl)phenyl(methyl)sulfane (**1k**)

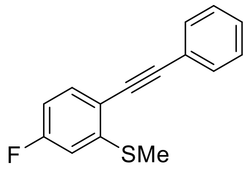

^{1}H NMR ($CDCl_3$, 500 MHz)

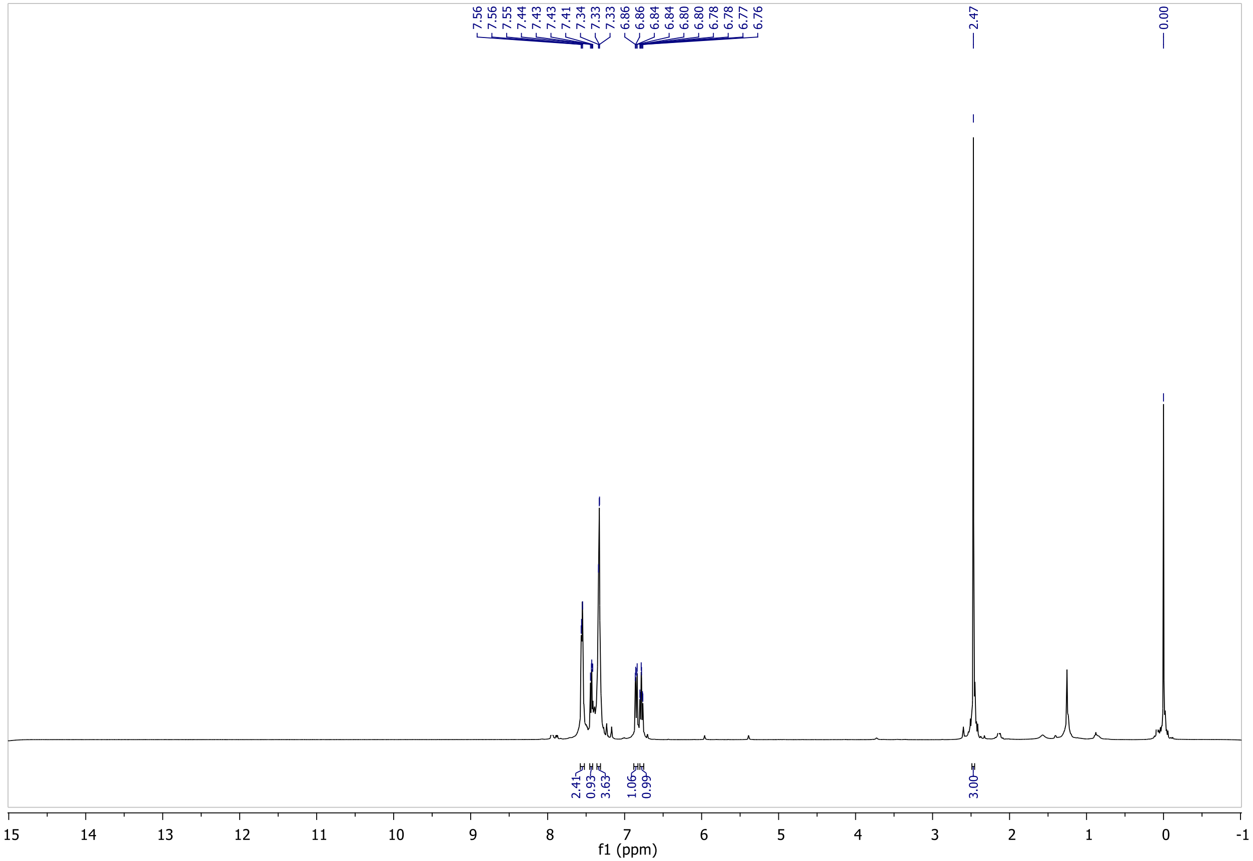

5-Fluoro-2-(phenylethynyl)phenyl(methyl)sulfane (**1k**)

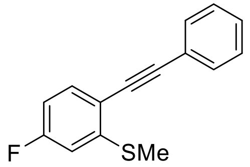

$^{13}C\{^{1}H\}$NMR ($CDCl_3$, 125 MHz)

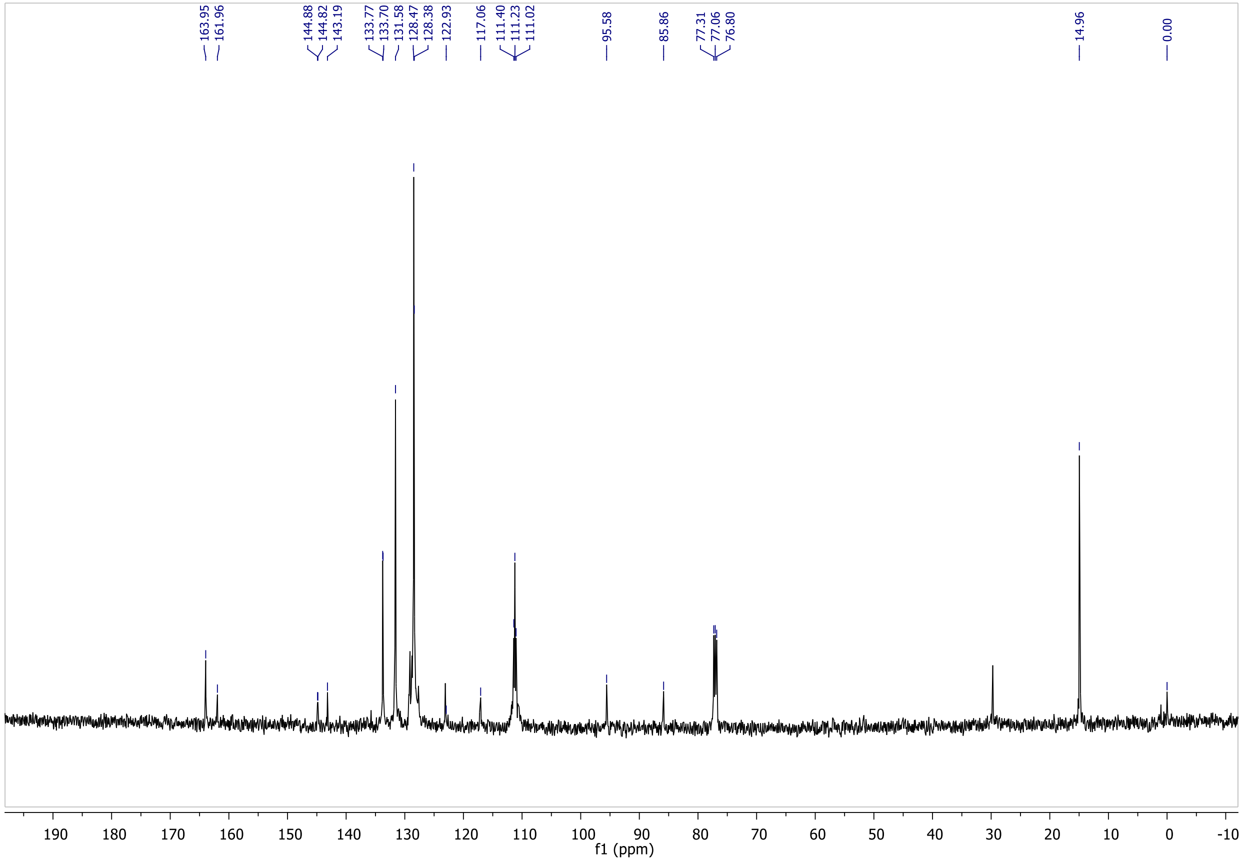

Methyl 2-phenylbenzo[*b*]thiophene-3-carboxylate (**2a**)

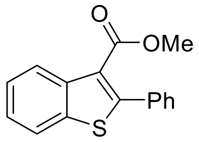

^{1}H NMR ($CDCl_3$, 300 MHz)

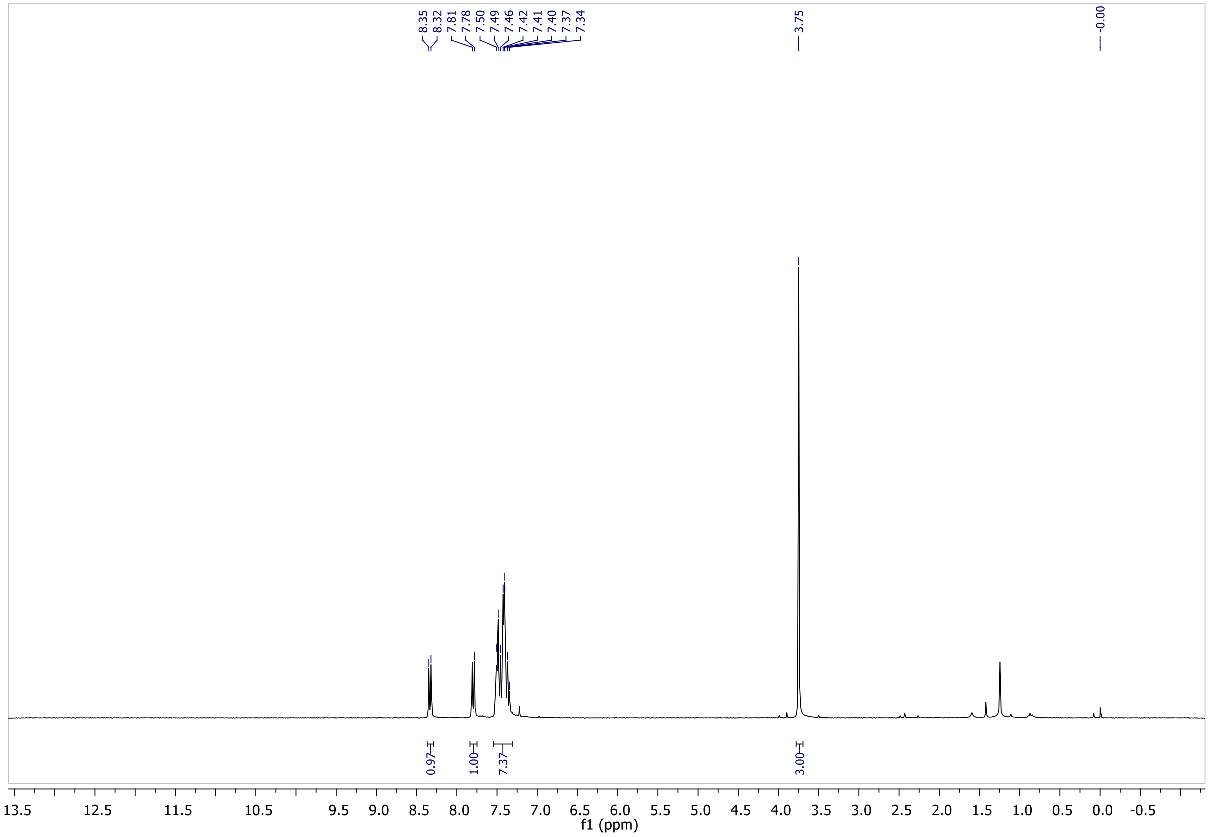

Methyl 2-phenylbenzo[*b*]thiophene-3-carboxylate (**2a**)

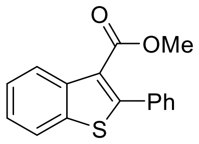

$^{13}C\{^{1}H\}$NMR ($CDCl_3$, 75 MHz)

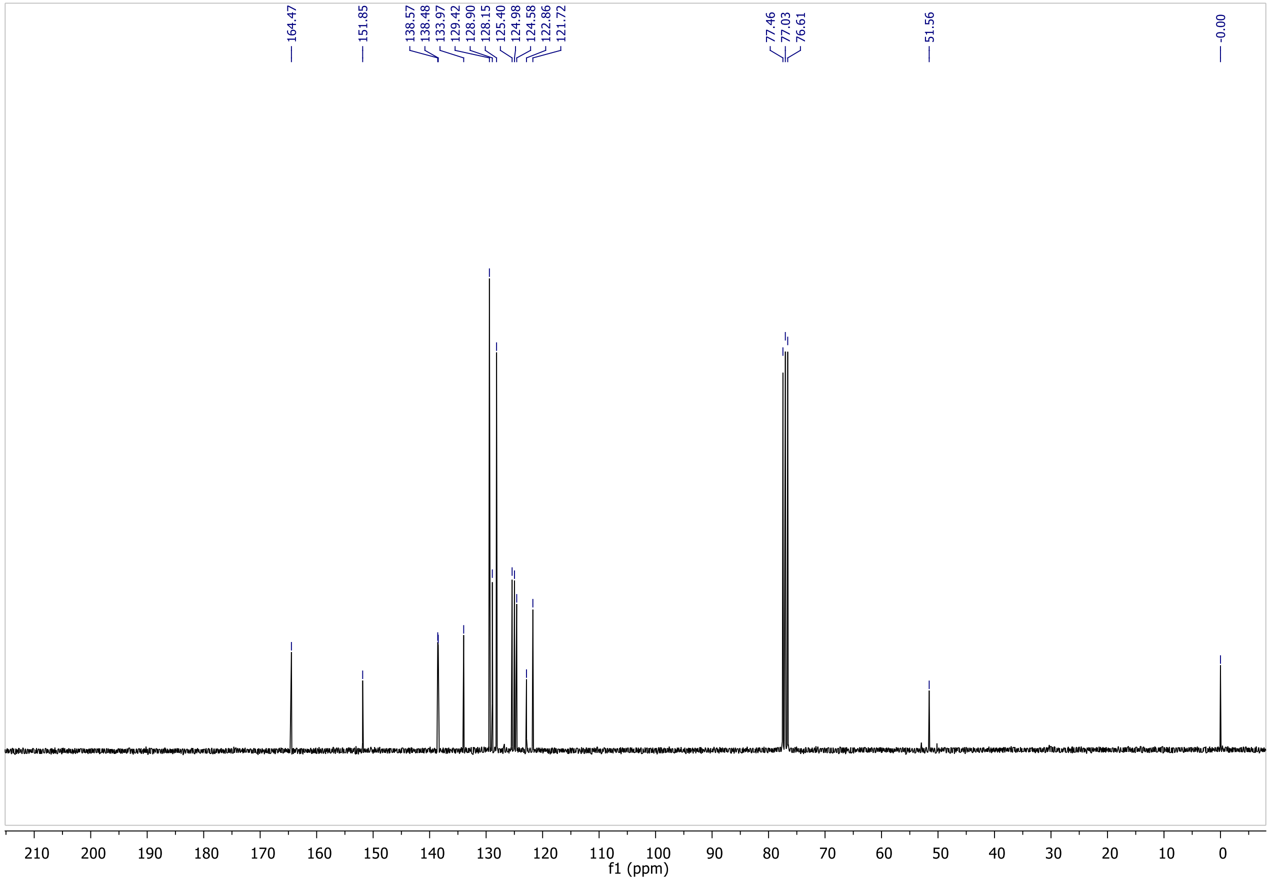

Ethyl 2-phenylbenzo[*b*]thiophene-3-carboxylate (**2a'**)

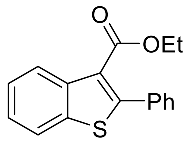

^{1}H NMR ($CDCl_3$, 500 MHz)

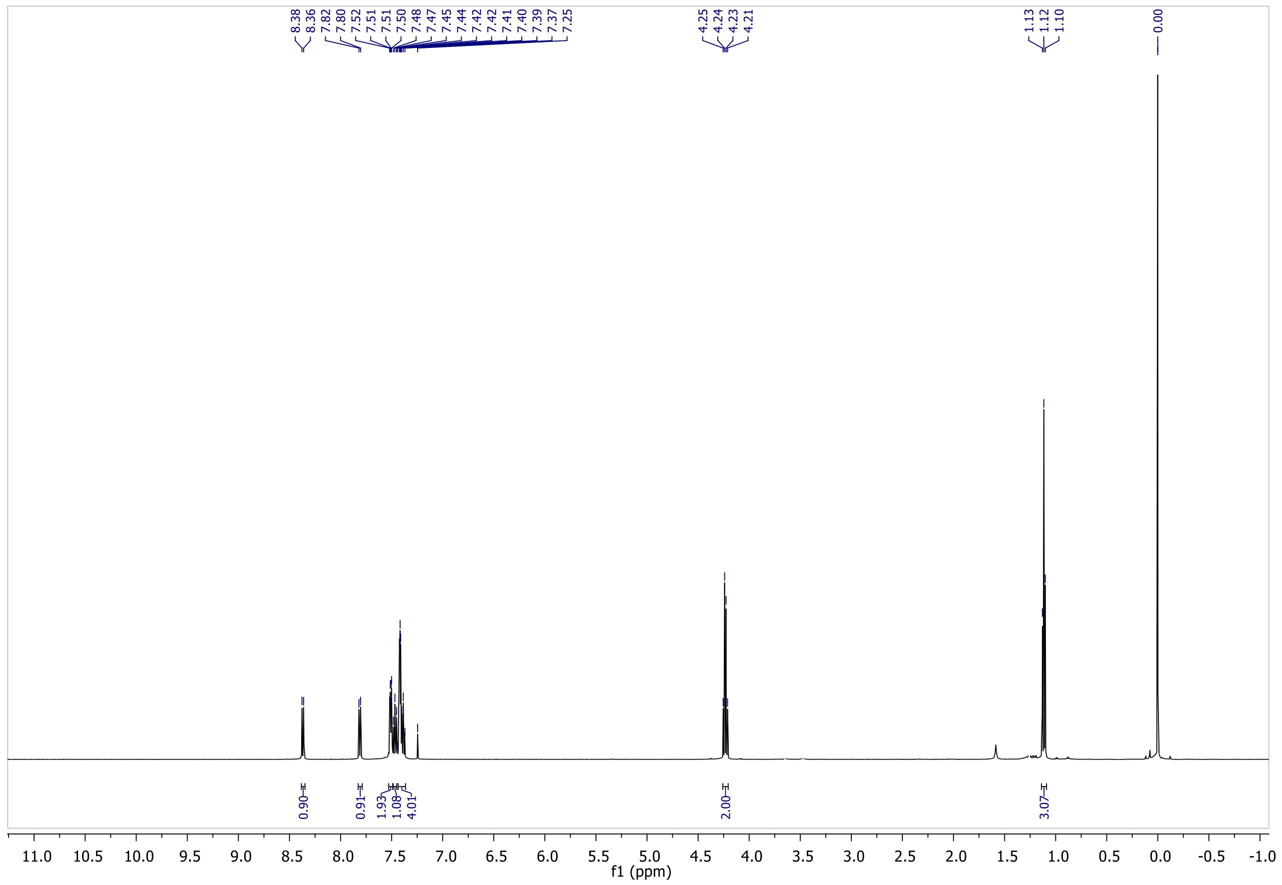

Ethyl 2-phenylbenzo[*b*]thiophene-3-carboxylate (**2a'**)

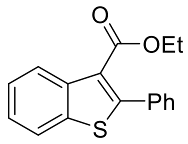

$^{13}C\{^{1}H\}$NMR ($CDCl_3$, 125 MHz)

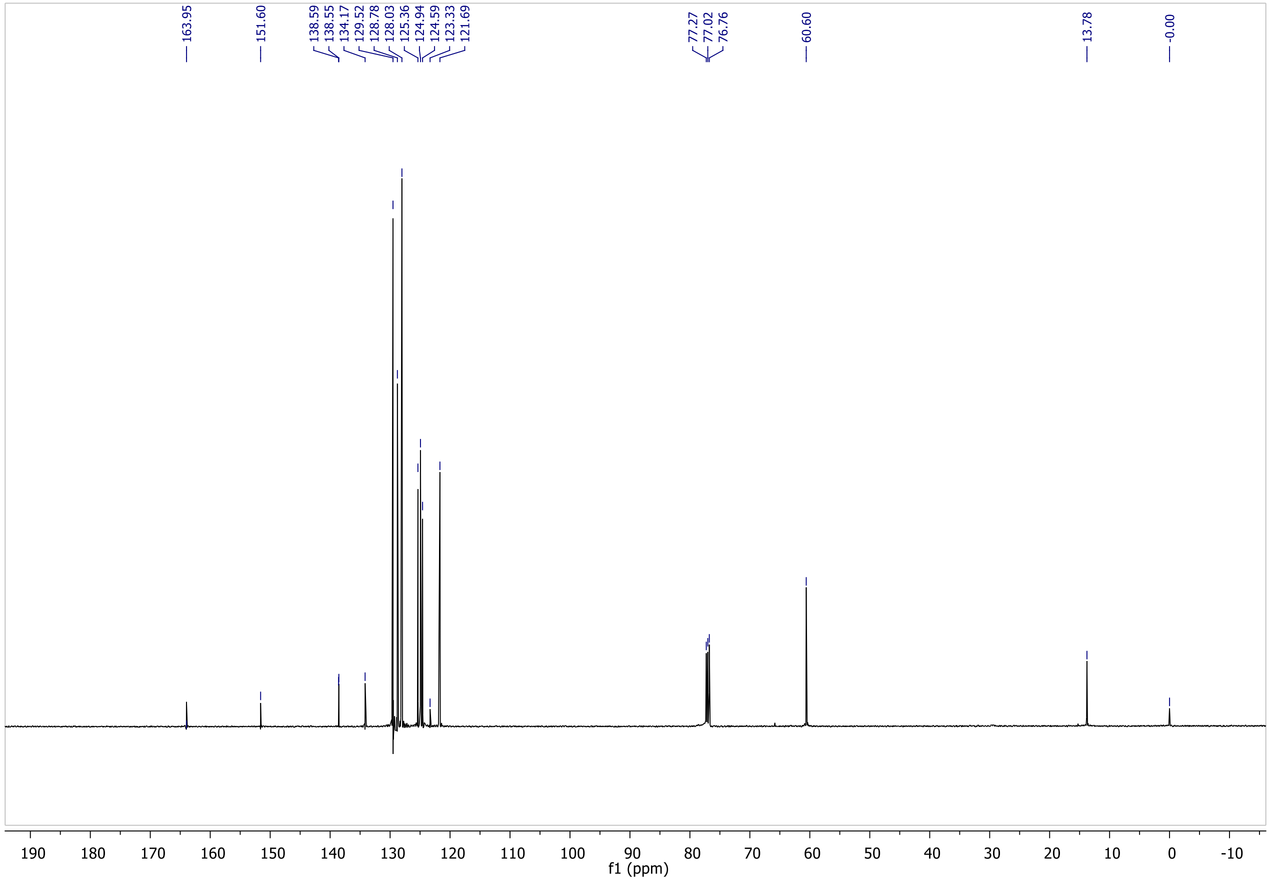

Isopropyl 2-phenylbenzo[*b*]thiophene-3-carboxylate (**2a''**)

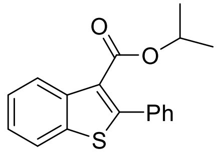

^{1}H NMR ($CDCl_3$, 500 MHz)

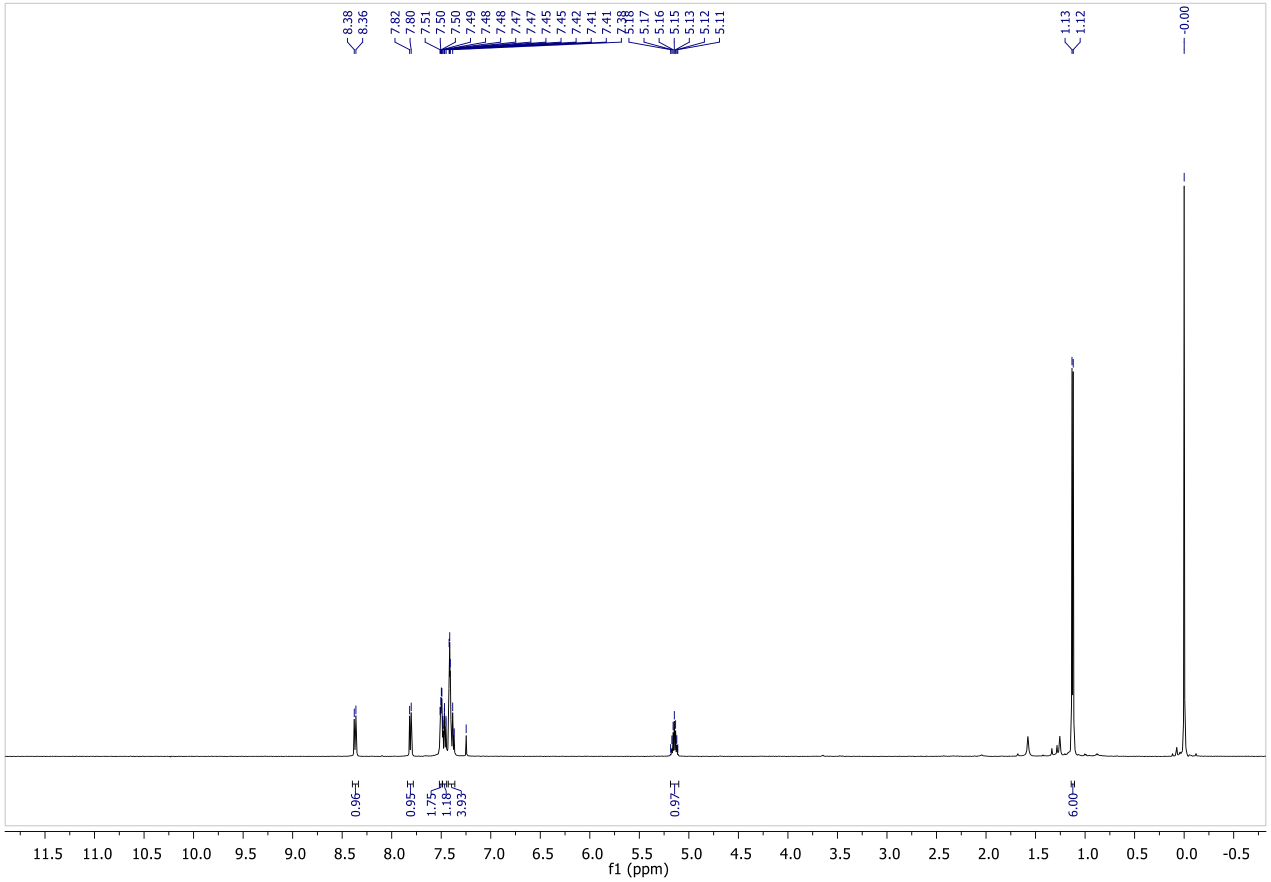

Isopropyl 2-phenylbenzo[*b*]thiophene-3-carboxylate (**2a''**)

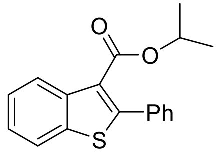

$^{13}C\{^{1}H\}$NMR ($CDCl_3$, 125 MHz)

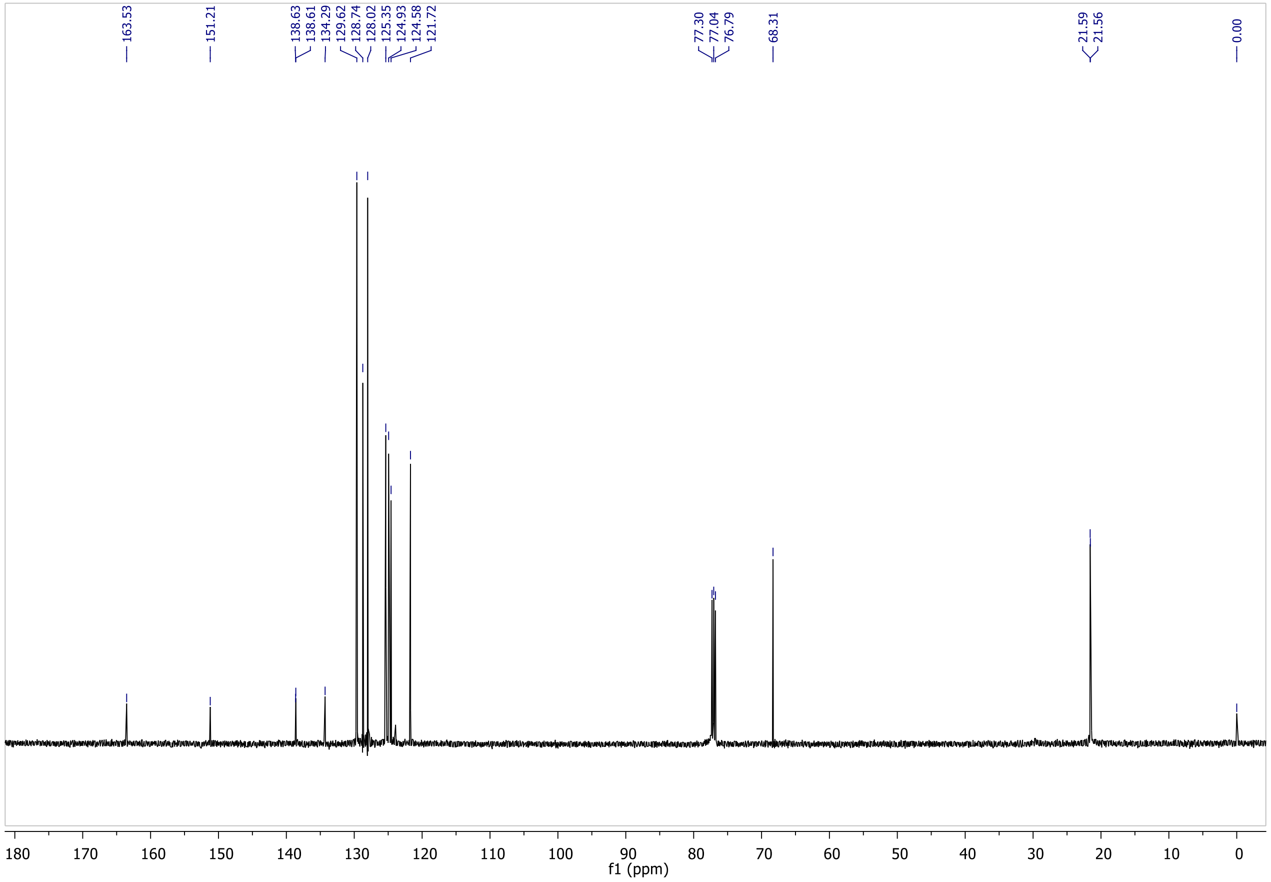

Methyl 2-(*p*-tolyl)benzo[*b*]thiophene-3-carboxylate (**2b**)

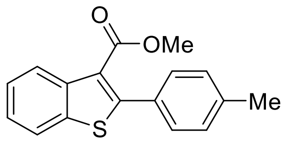

^{1}H NMR ($CDCl_3$, 300 MHz)

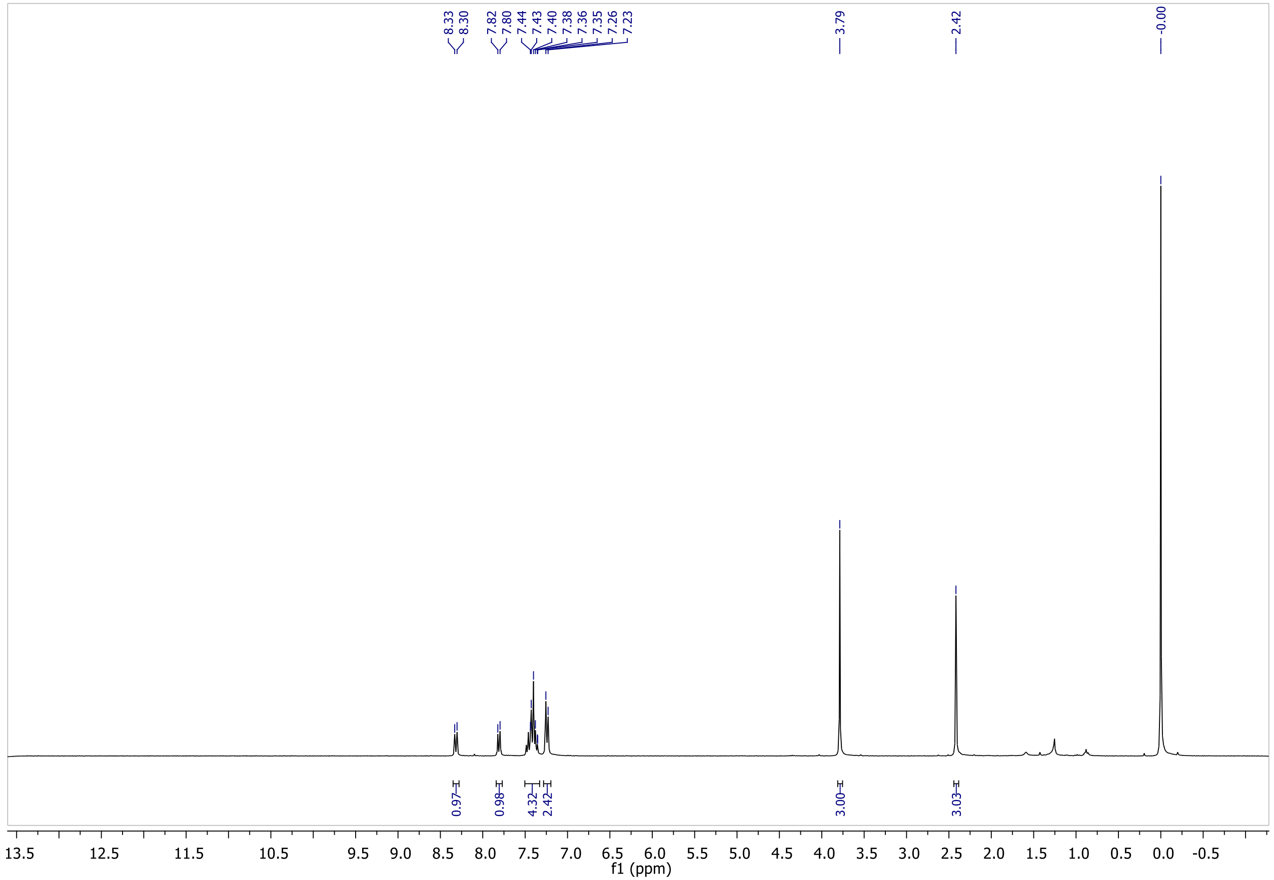

Methyl 2-(*p*-tolyl)benzo[*b*]thiophene-3-carboxylate (**2b**)

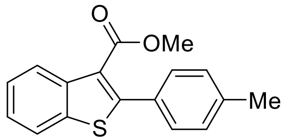

$^{13}C\{^1H\}$NMR ($CDCl_3$, 75 MHz)

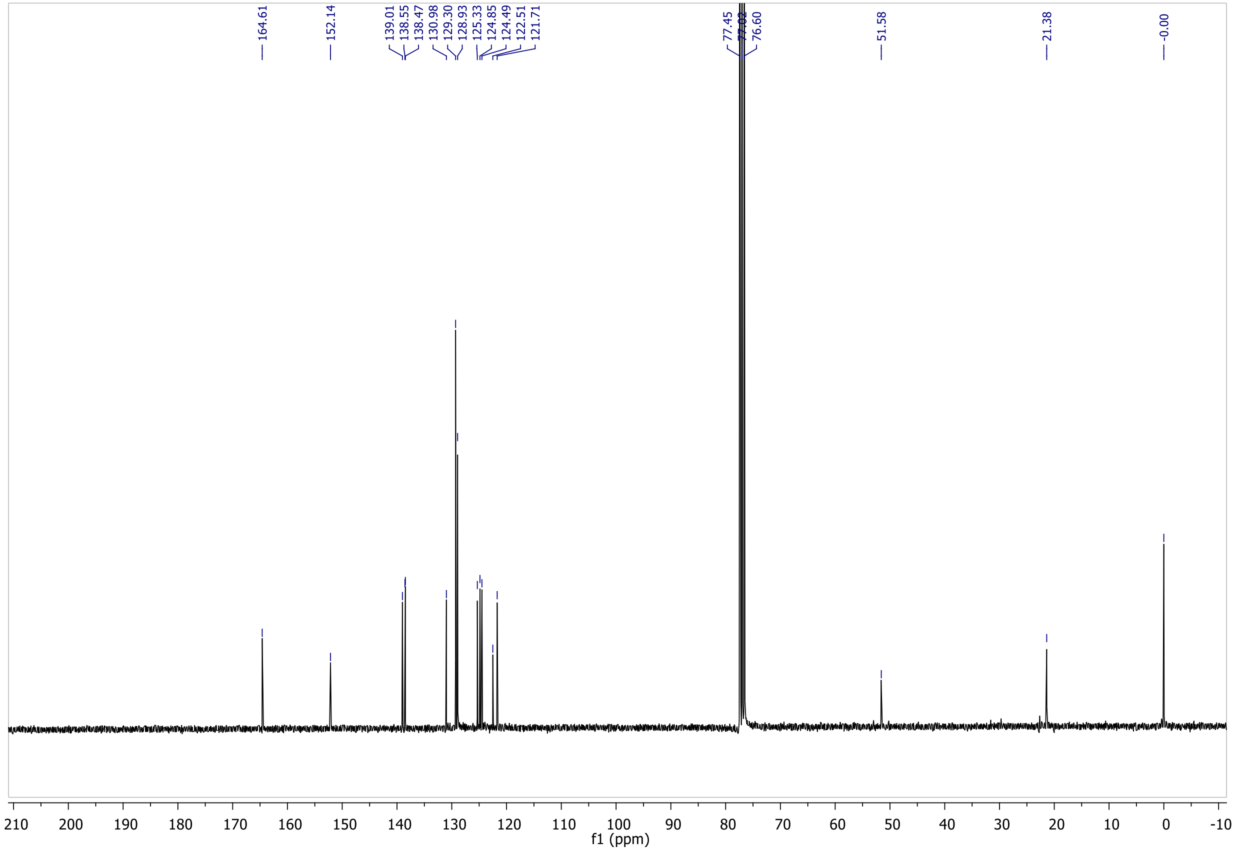

Methyl 2-(4-bromophenyl)benzo[*b*]thiophene-3-carboxylate (**2c**)

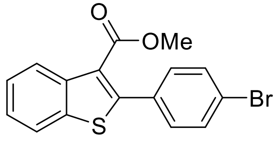

^{1}H NMR ($CDCl_3$, 500 MHz)

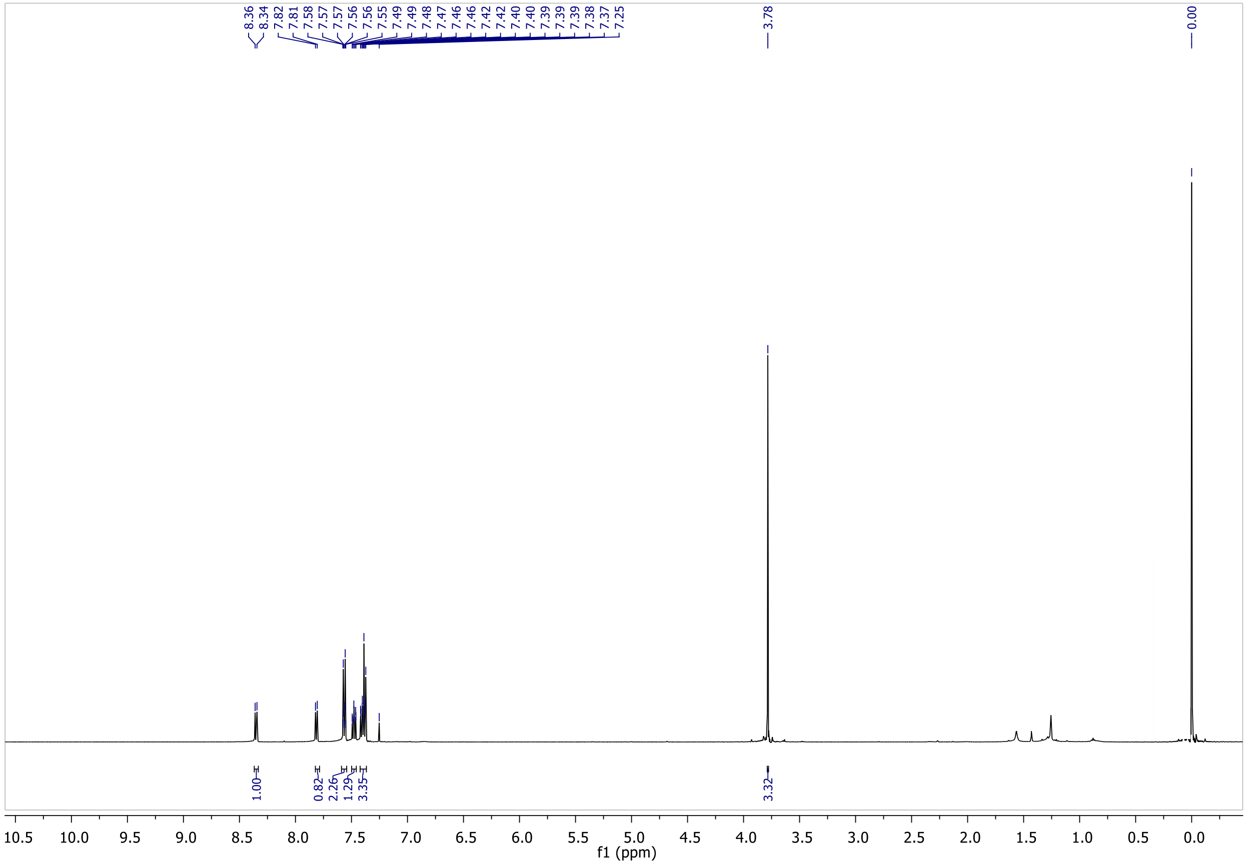

Methyl 2-(4-bromophenyl)benzo[*b*]thiophene-3-carboxylate (**2c**)

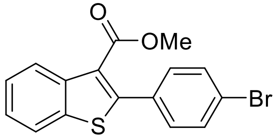

$^{13}C\{^{1}H\}$NMR ($CDCl_3$, 125 MHz)

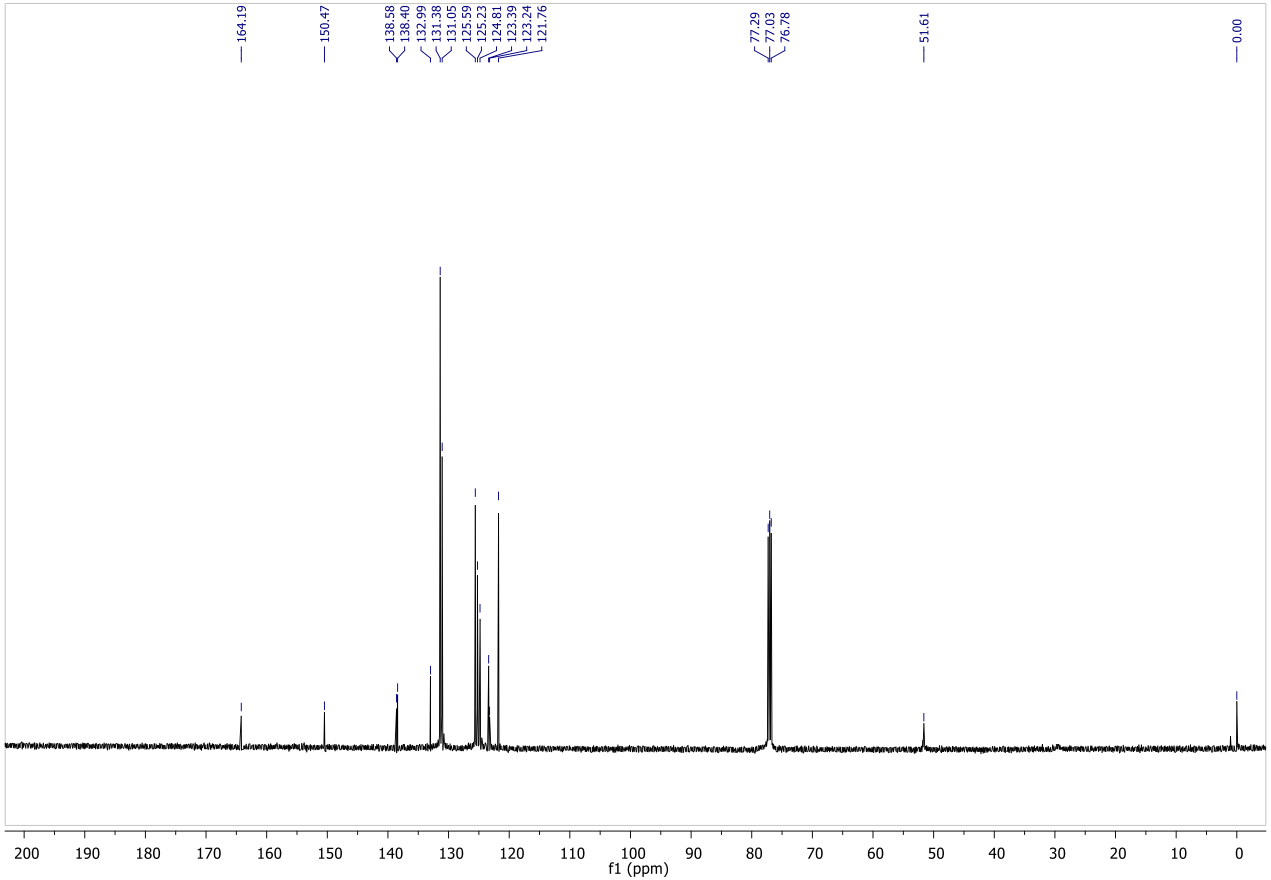

Methyl 2-(thiophen-3-yl)benzo[*b*]thiophene-3-carboxylate (**2d**)

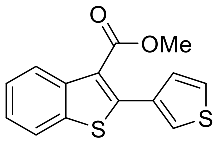

^{1}H NMR ($CDCl_3$, 300 MHz)

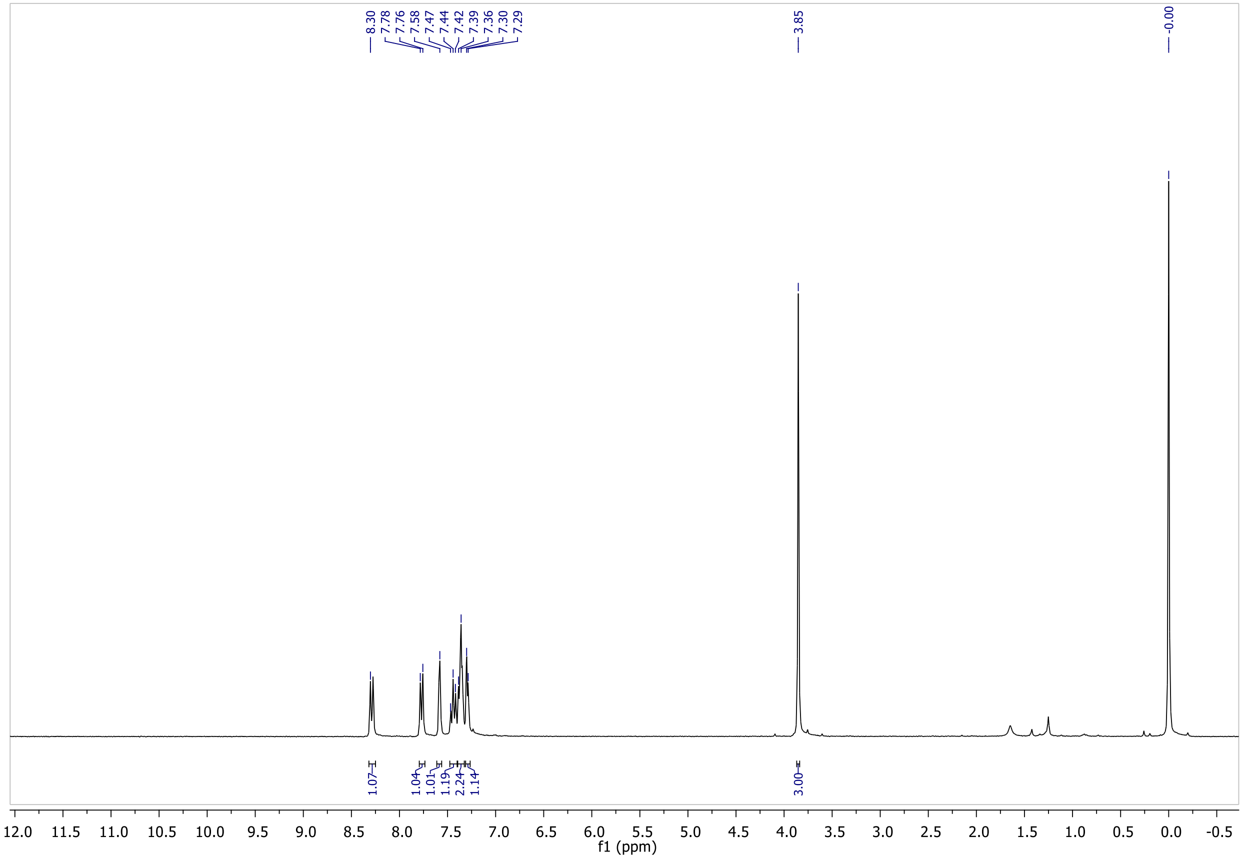

Methyl 2-(thiophen-3-yl)benzo[*b*]thiophene-3-carboxylate (**2d**)

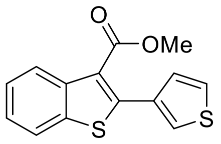

$^{13}C\{^{1}H\}$NMR ($CDCl_3$, 75 MHz)

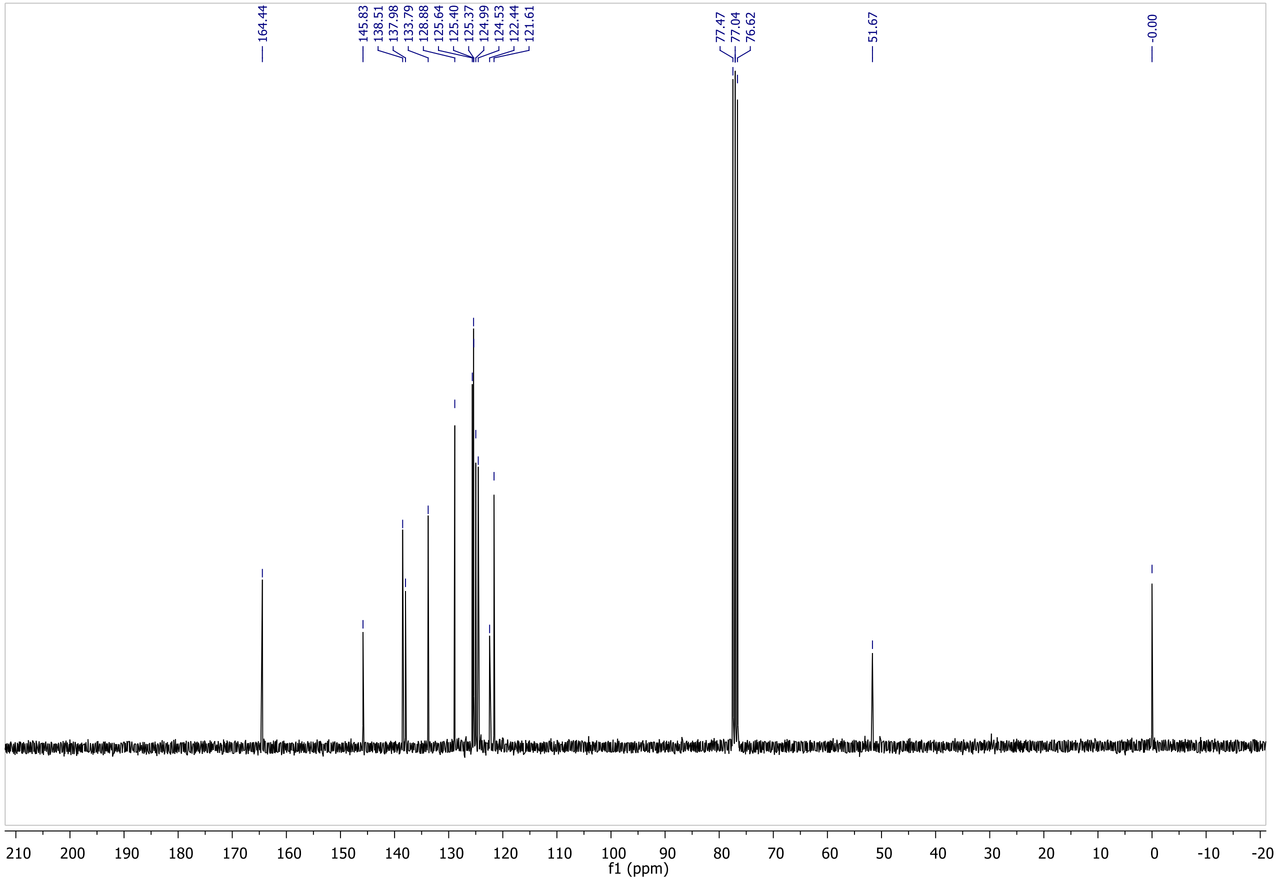

Methyl 2-(cyclohex-1-en-1-yl)benzo[*b*]thiophene-3-carboxylate (**2e**)

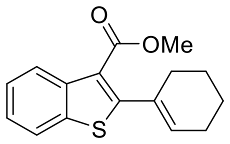

^{1}H NMR ($CDCl_3$, 300 MHz)

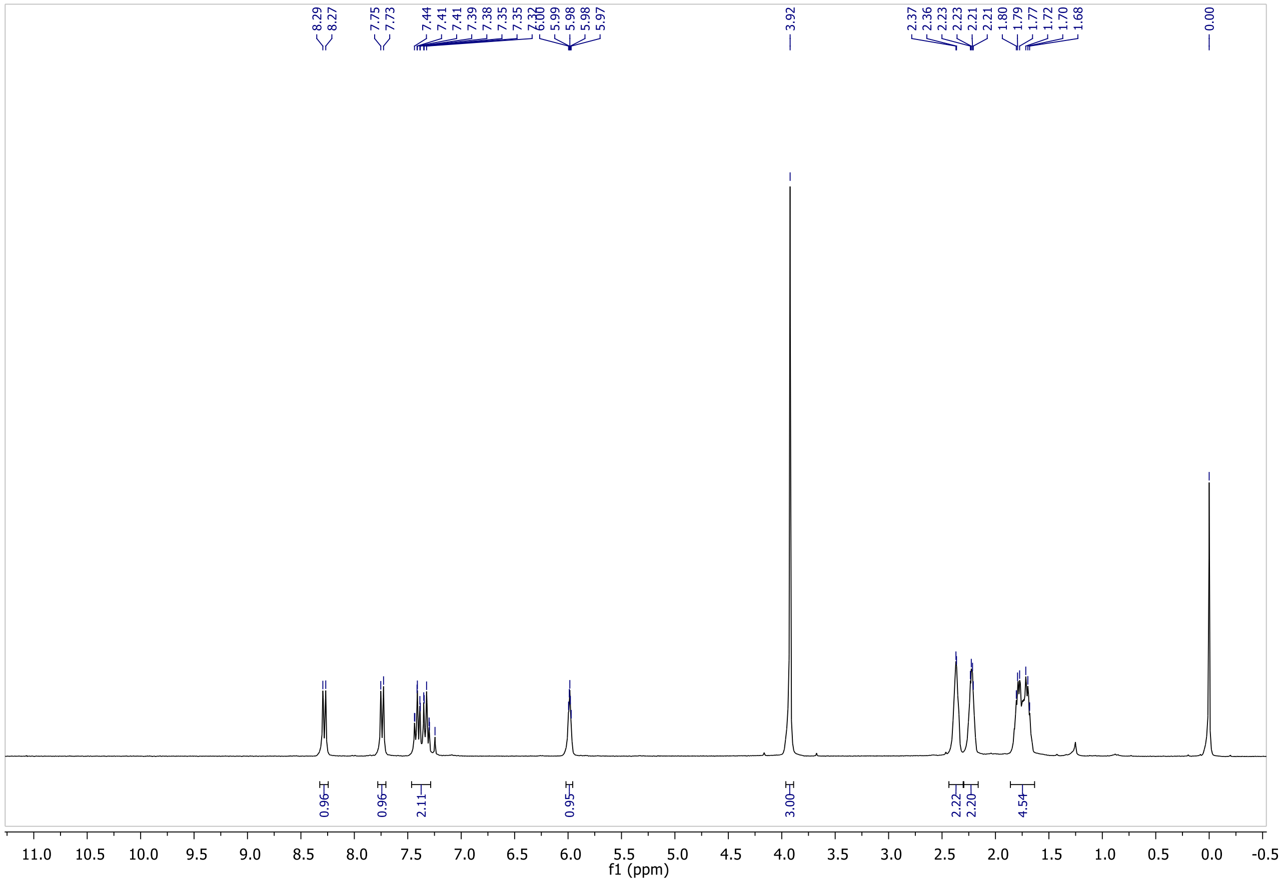

Methyl 2-(cyclohex-1-en-1-yl)benzo[*b*]thiophene-3-carboxylate (**2e**)

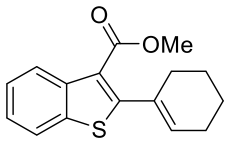

$^{13}C\{^{1}H\}$NMR ($CDCl_3$, 75 MHz)

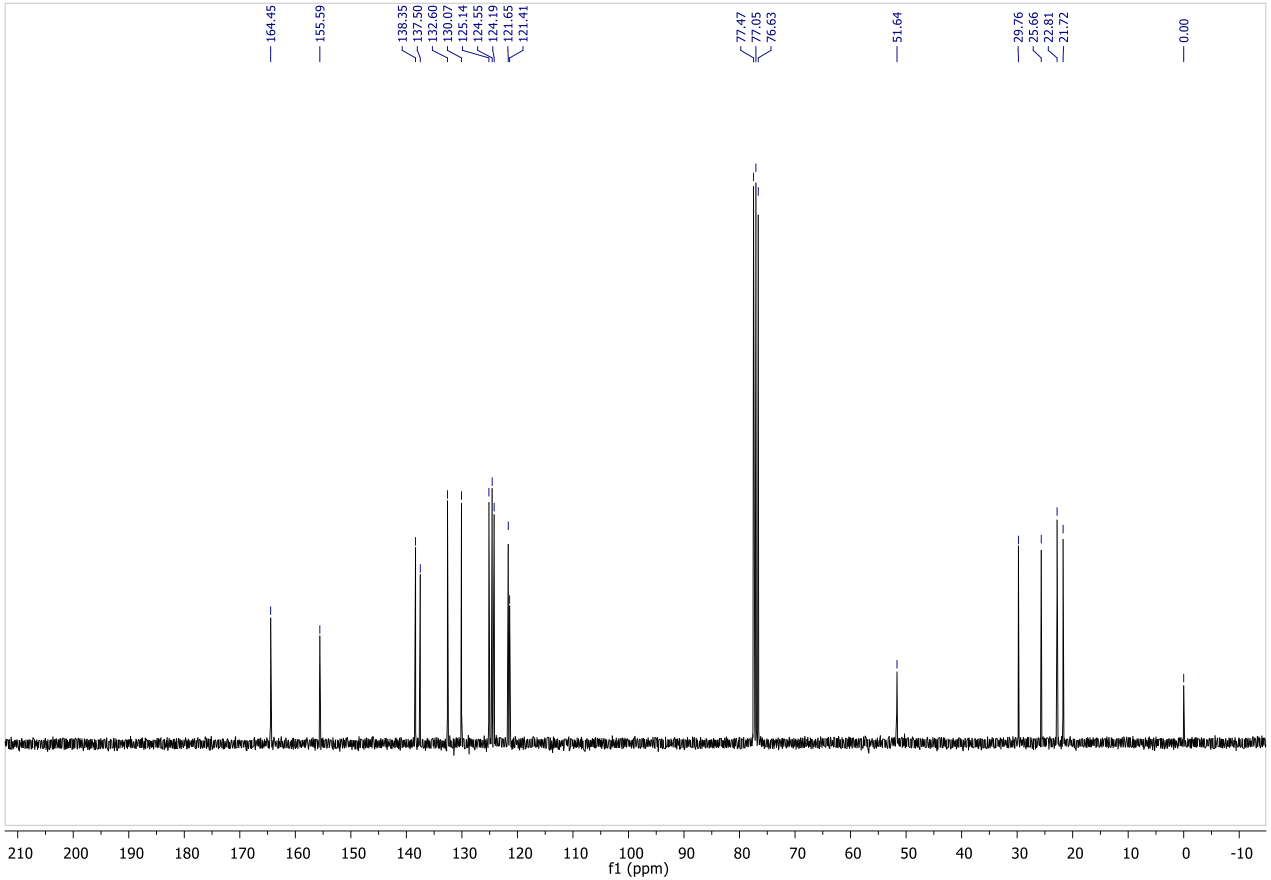

Methyl 2-butylbenzo[*b*]thiophene-3-carboxylate (**2f**)

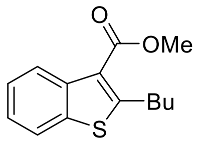

^{1}H NMR ($CDCl_3$, 500 MHz)

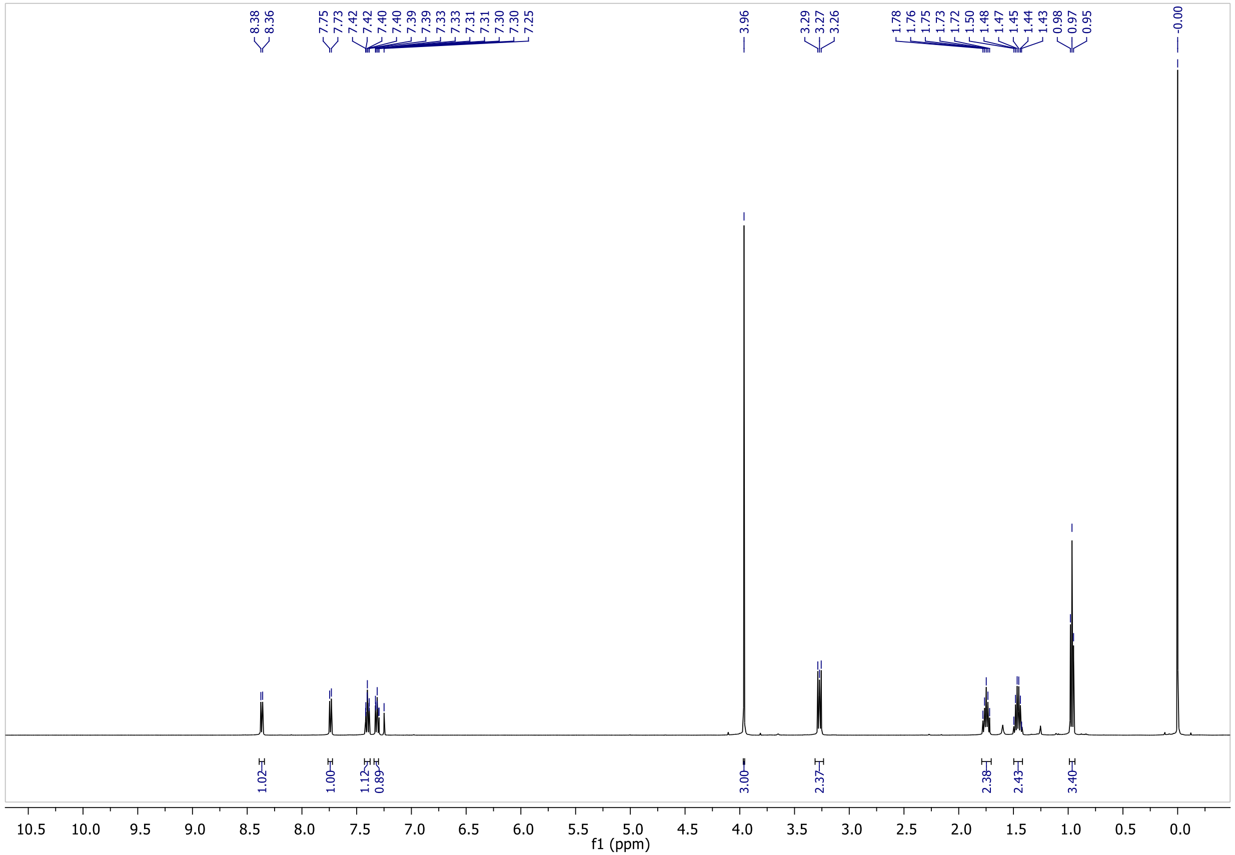

Methyl 2-butylbenzo[*b*]thiophene-3-carboxylate (**2f**)

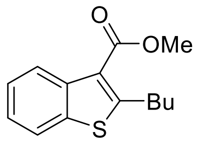

$^{13}C\{^{1}H\}$NMR ($CDCl_3$, 125 MHz)

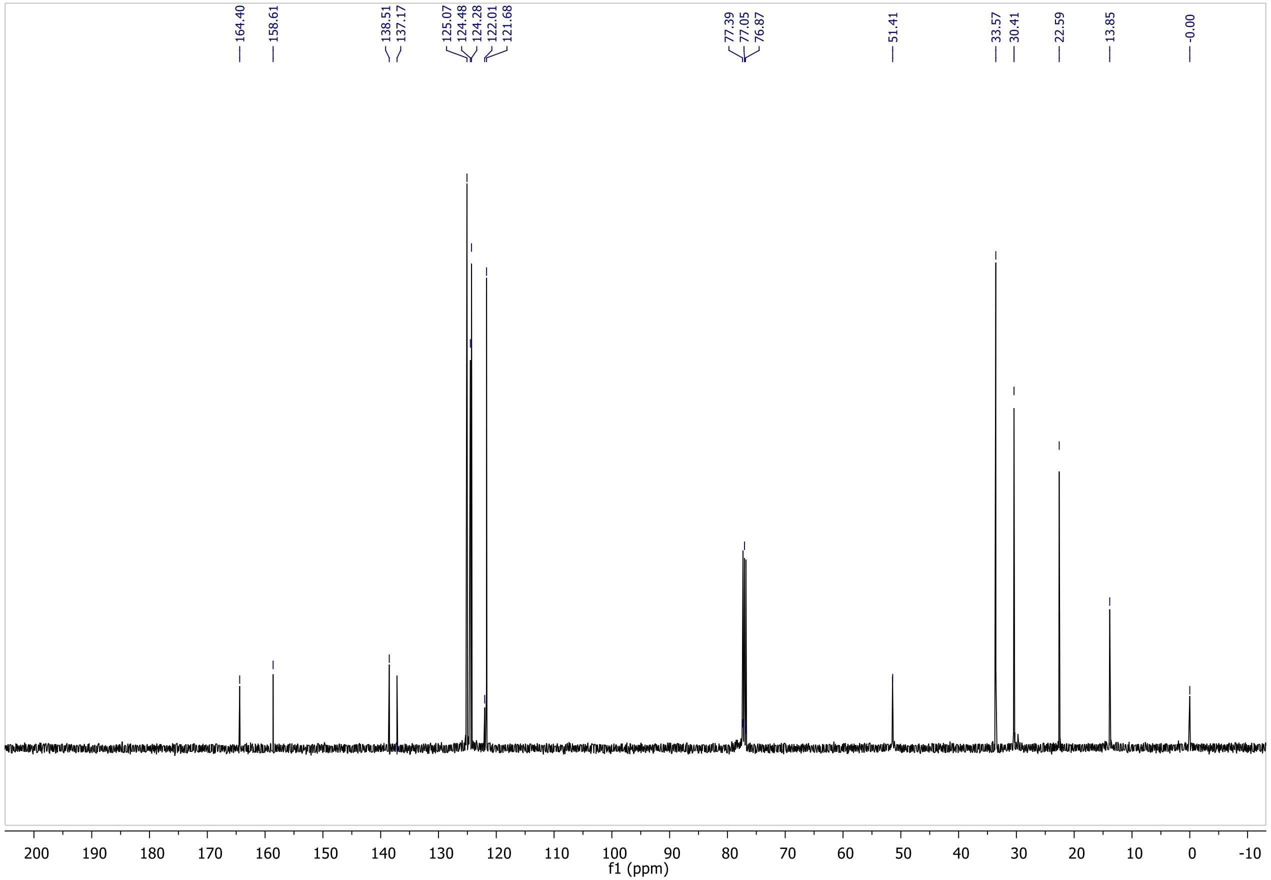

Methyl 2-phenethylbenzo[*b*]thiophene-3-carboxylate (**2g**)

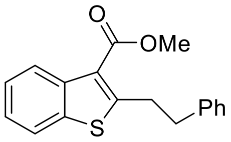

^{1}H NMR ($CDCl_3$, 500 MHz)

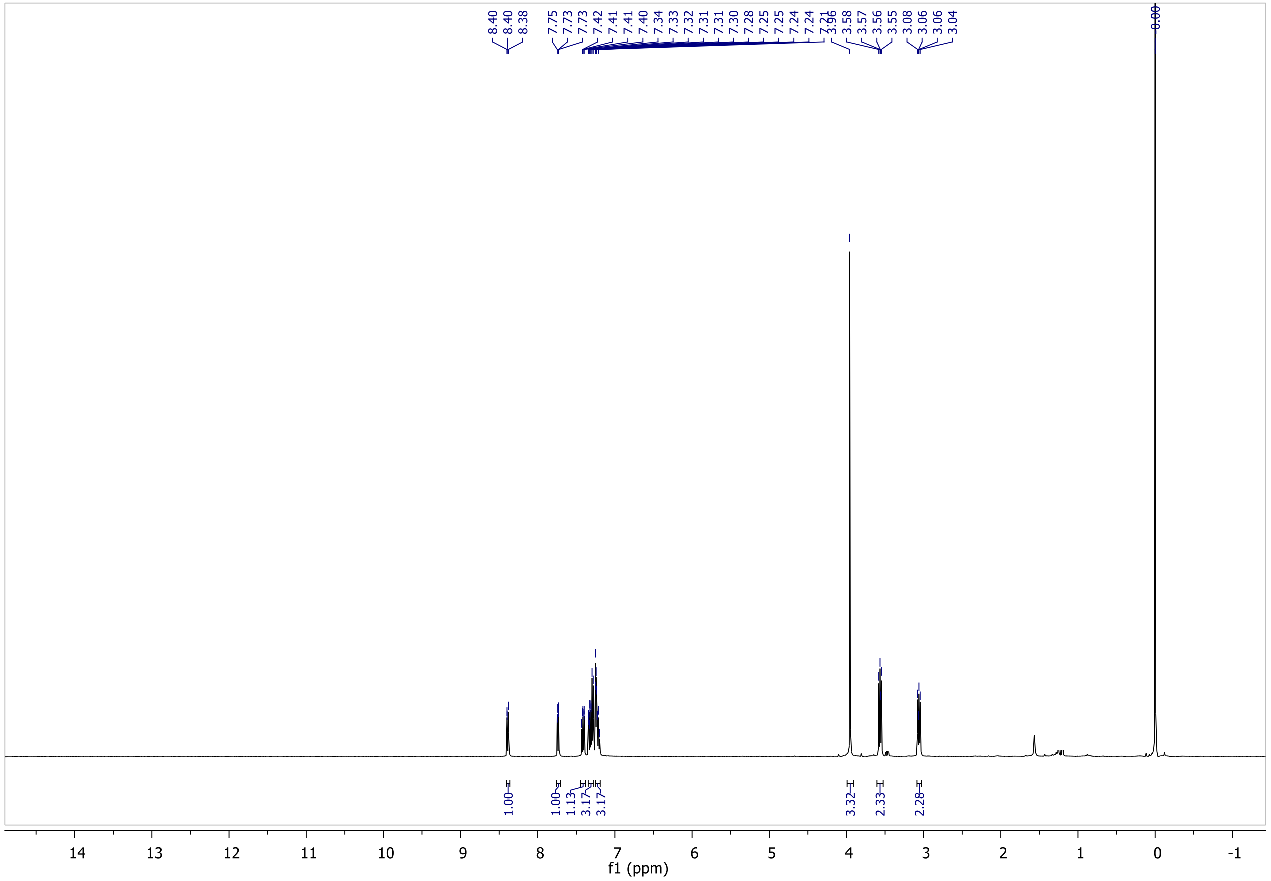

Methyl 2-phenethylbenzo[*b*]thiophene-3-carboxylate (**2g**)

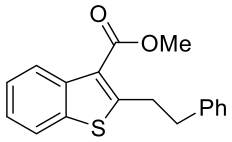

$^{13}C\{^1H\}$NMR ($CDCl_3$, 125 MHz)

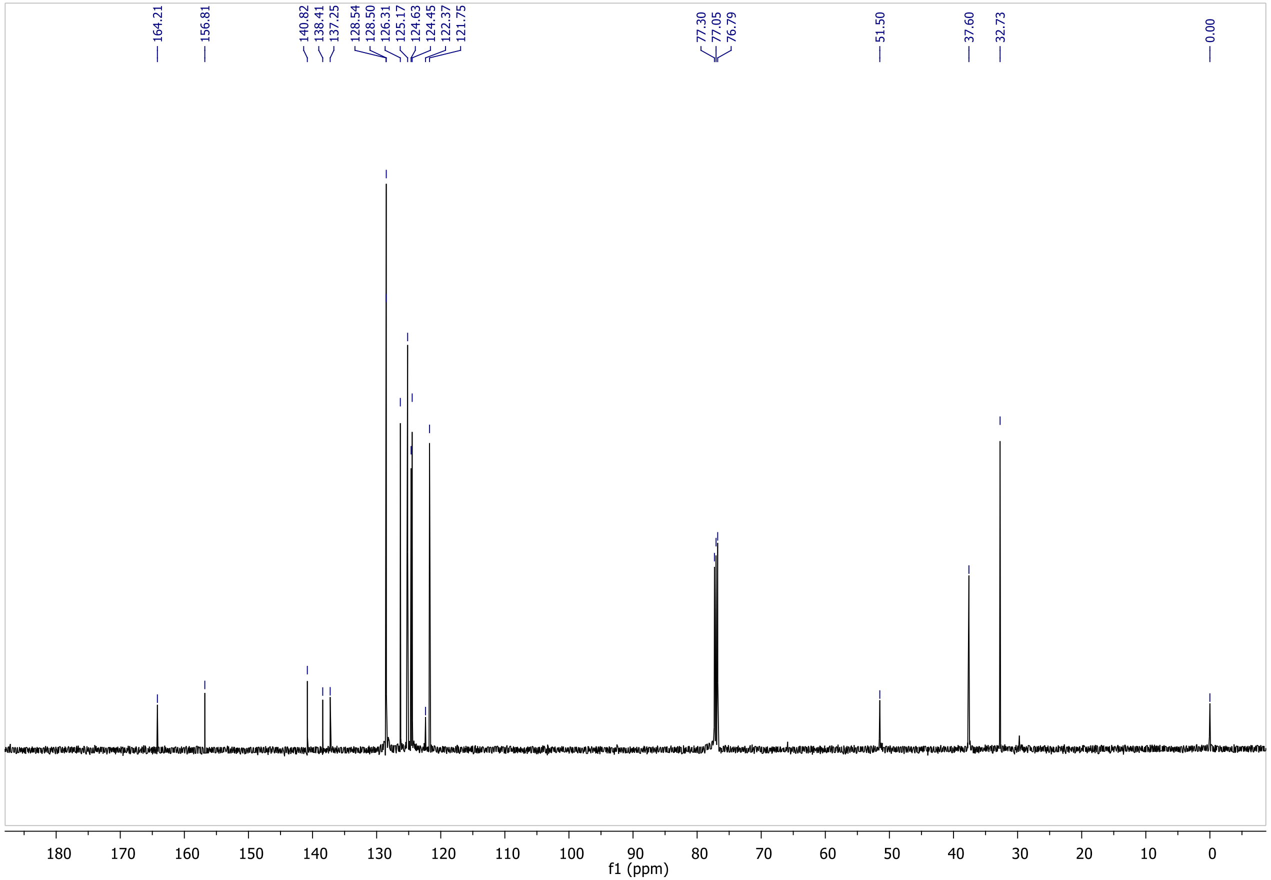

Methyl 2-(*tert*-butyl)benzo[*b*]thiophene-3-carboxylate (**2h**)

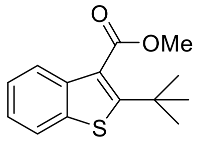

^{1}H NMR ($CDCl_3$, 300MHz)

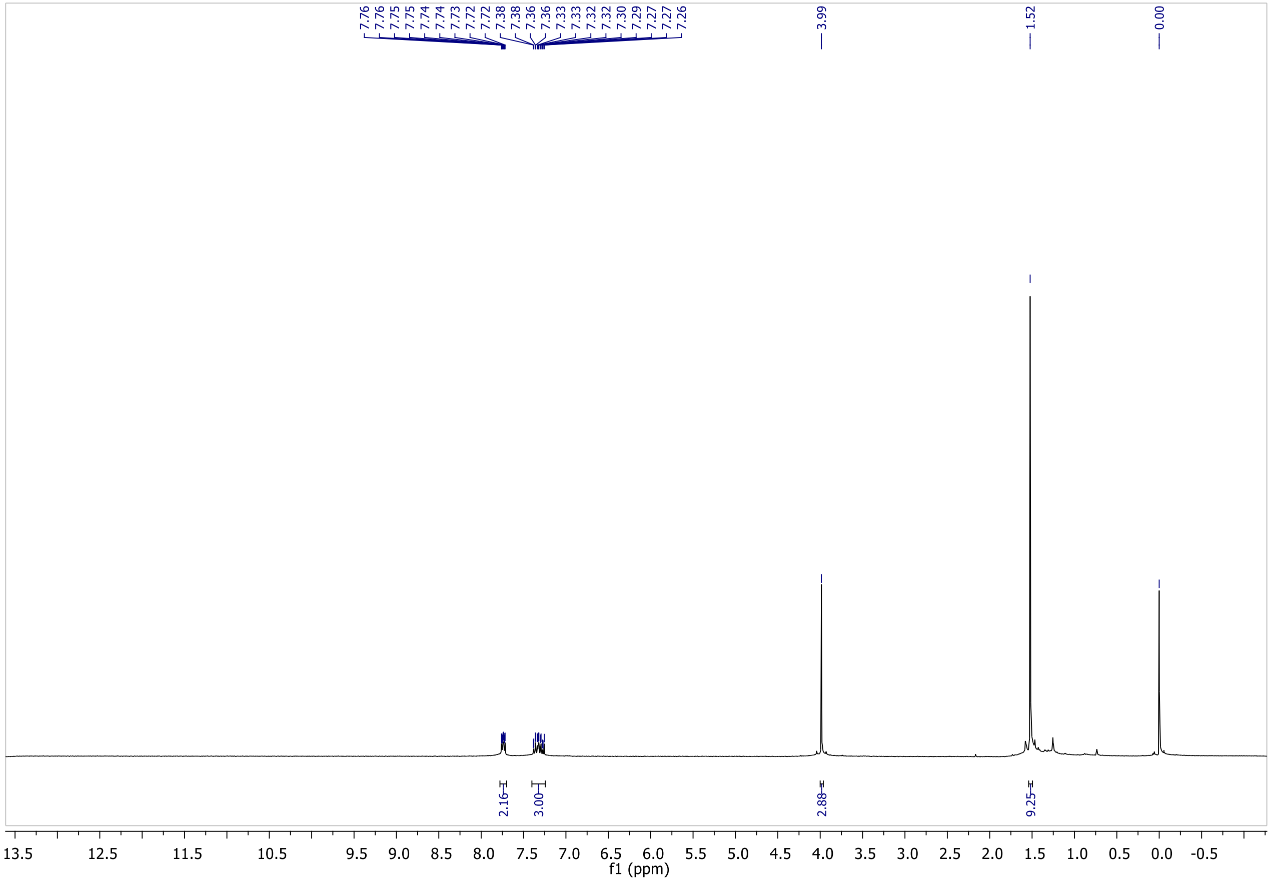

Methyl 2-(*tert*-butyl)benzo[*b*]thiophene-3-carboxylate (**2h**)

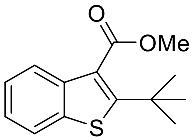

$^{13}C\{^{1}H\}$NMR ($CDCl_3$, 75 MHz)

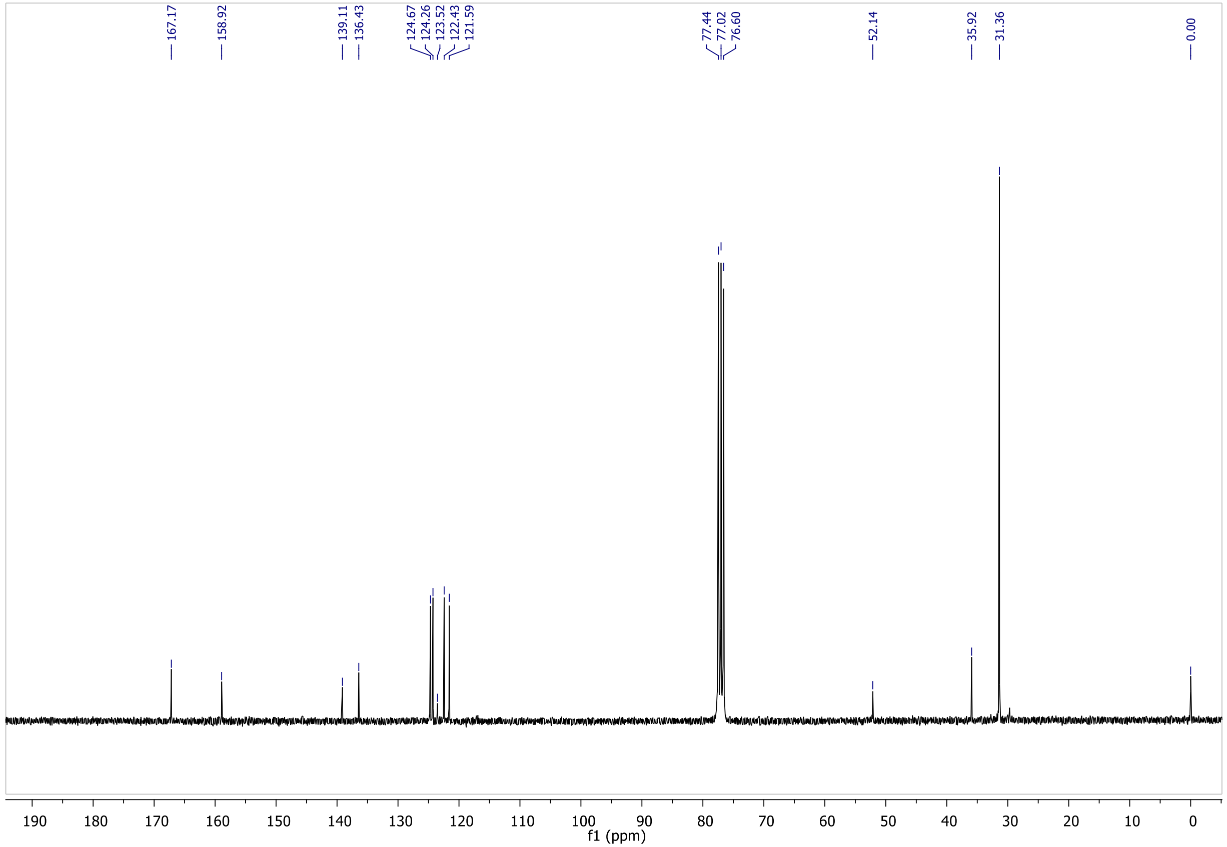

Methyl 5-methyl-2-phenylbenzo[*b*]thiophene-3-carboxylate (**2i**)

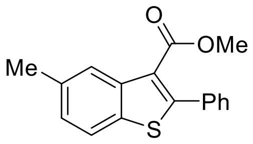

^{1}H NMR ($CDCl_3$, 300 MHz)

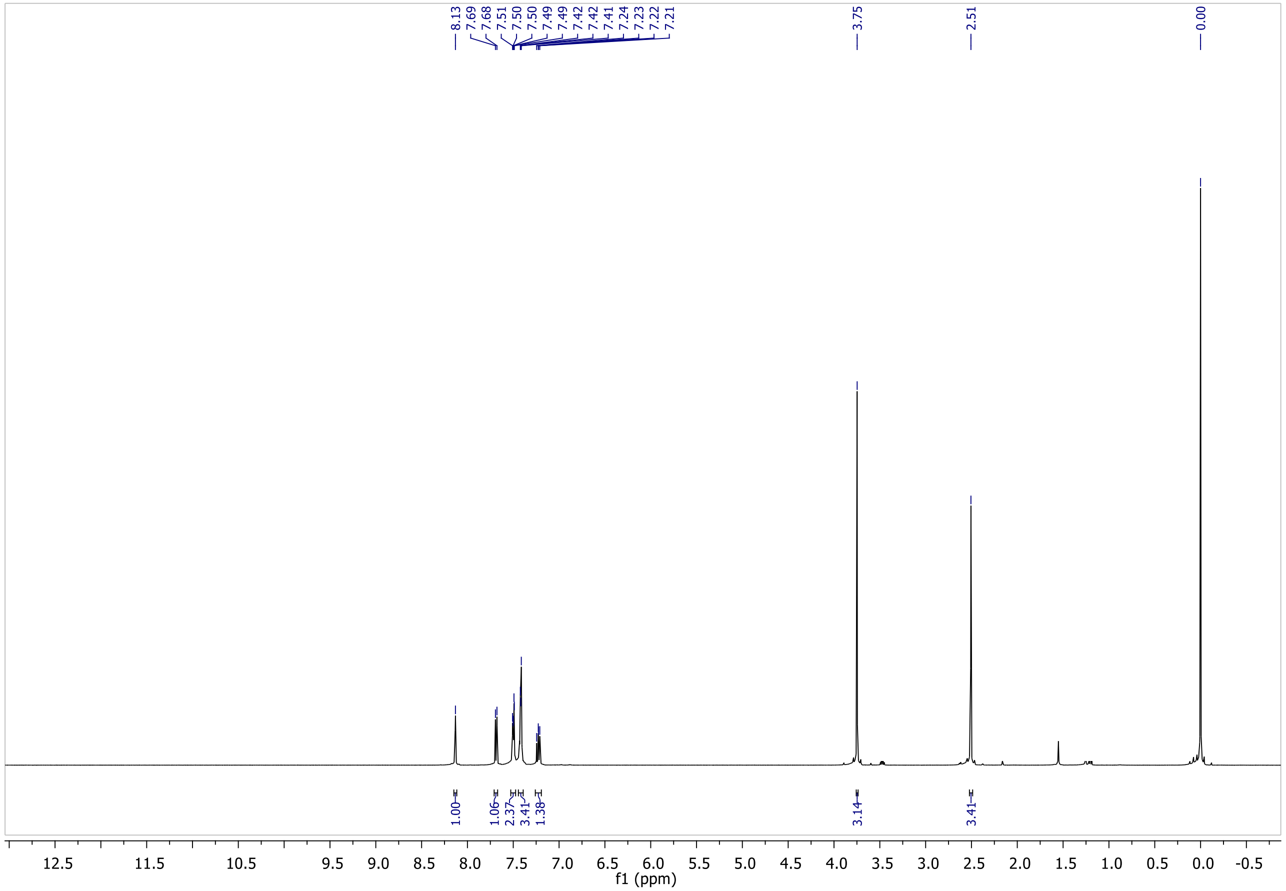

Methyl 5-methyl-2-phenylbenzo[*b*]thiophene-3-carboxylate (**2i**)

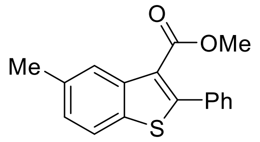

$^{13}C\{^{1}H\}$NMR ($CDCl_3$, 75 MHz)

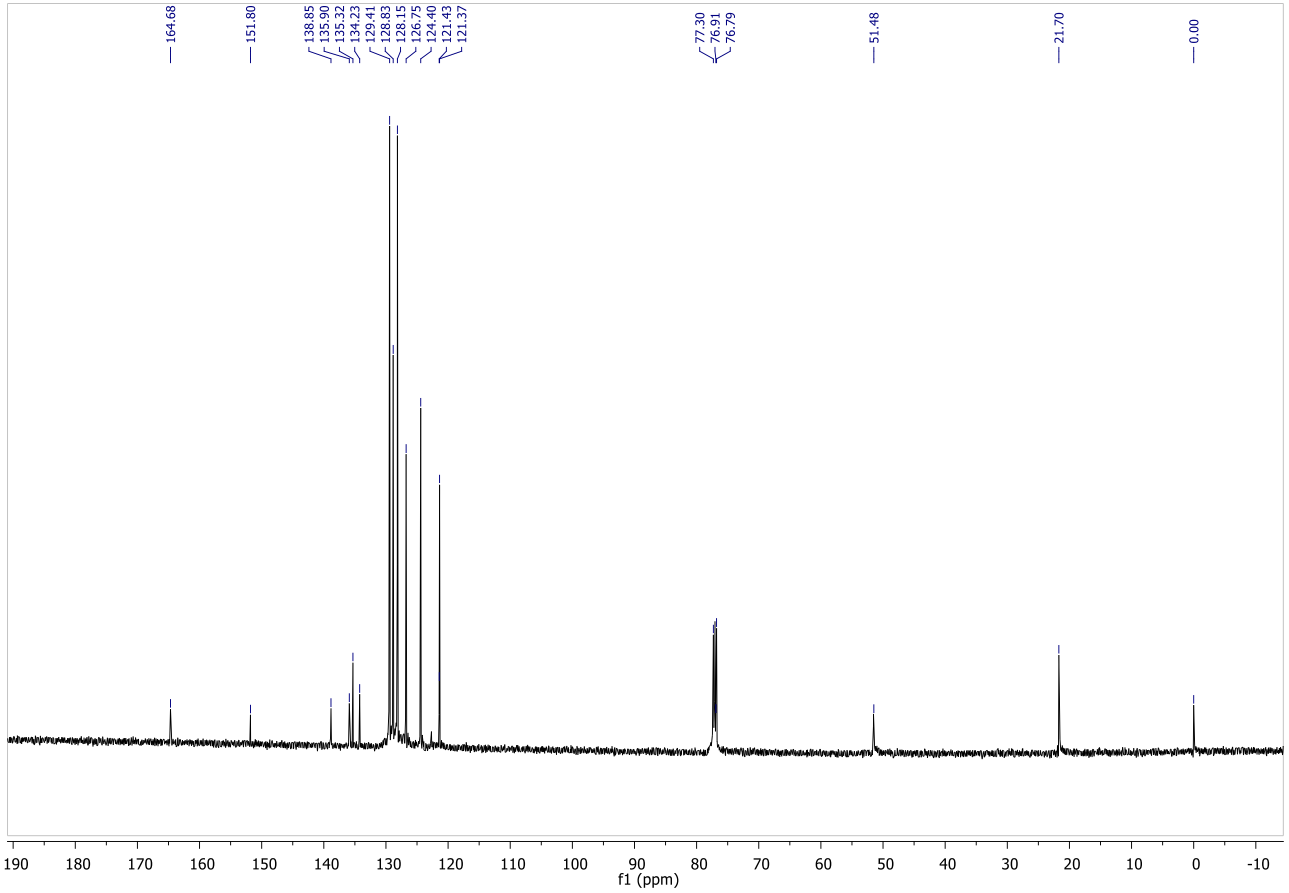

Methyl 5-fluoro-2-phenylbenzo[b]thiophene-3-carboxylate (**2j**)

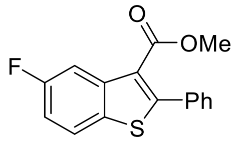

^{1}H NMR ($CDCl_3$, 300 MHz)

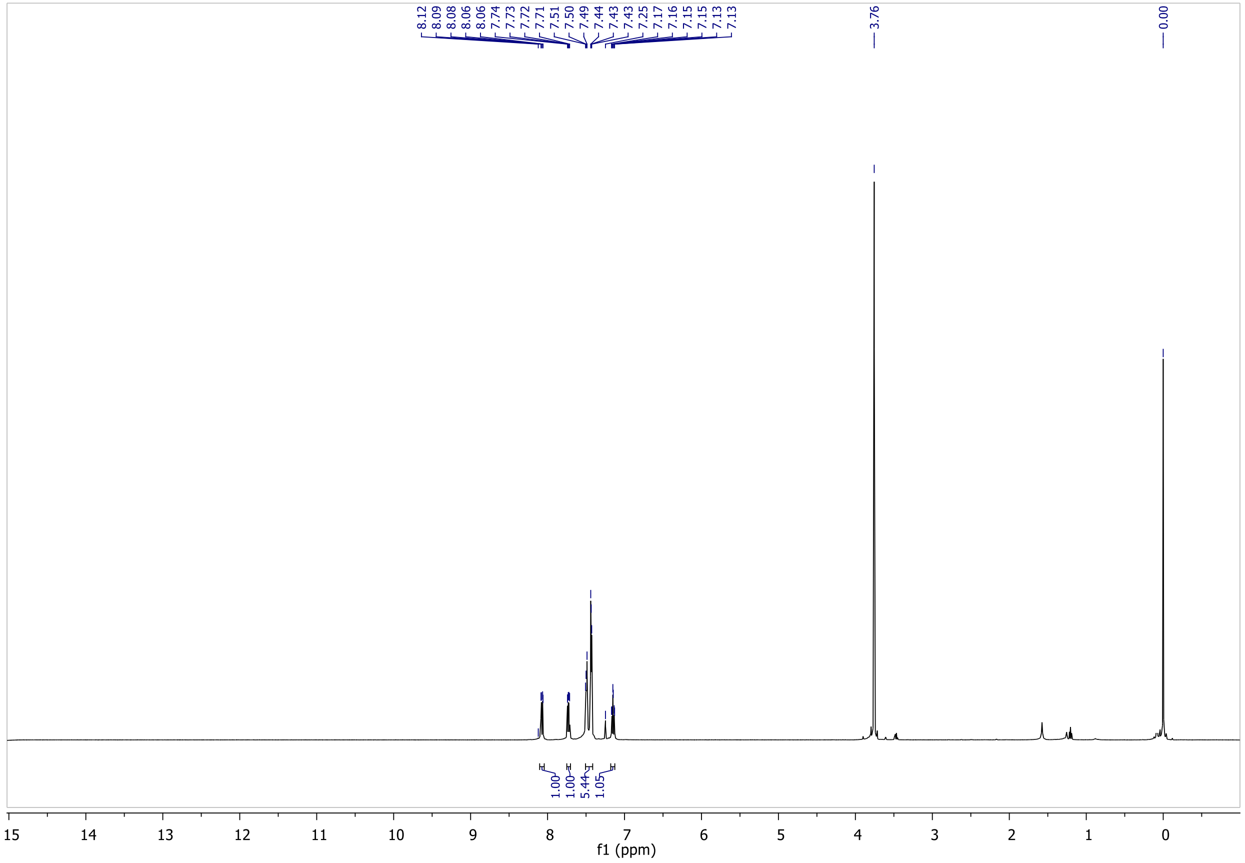

Methyl 5-fluoro-2-phenylbenzo[b]thiophene-3-carboxylate (**2j**)

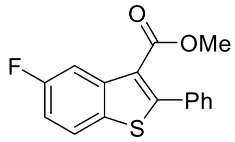

$^{13}C\{^{1}H\}$NMR ($CDCl_3$, 75 MHz)

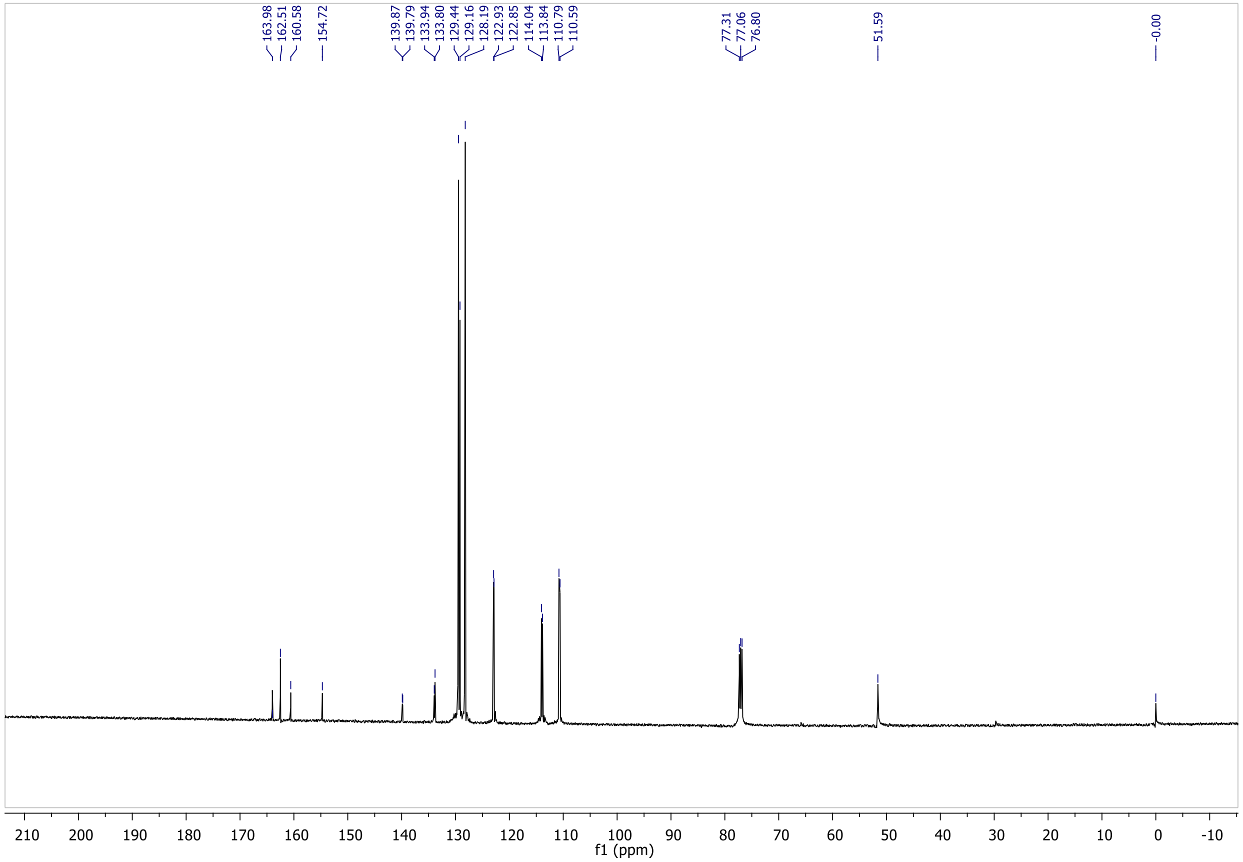

Methyl 6-fluoro-2-phenylbenzo[b]thiophene-3-carboxylate (**2k**)

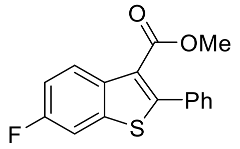

^{1}H NMR ($CDCl_3$, 300 MHz)

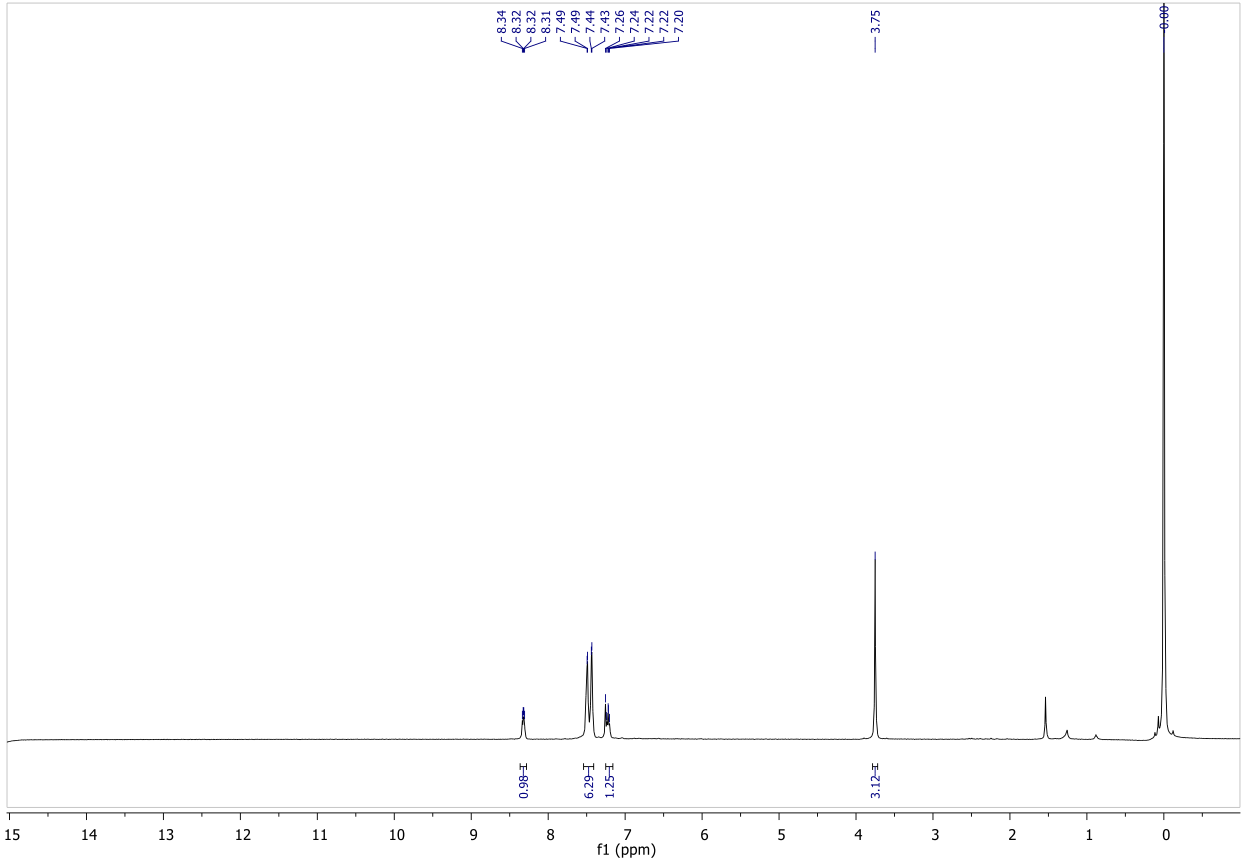

Methyl 6-fluoro-2-phenylbenzo[*b*]thiophene-3-carboxylate (**2k**)

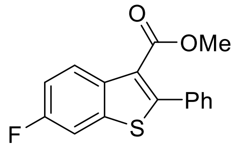

$^{13}C\{^{1}H\}$NMR ($CDCl_3$, 75 MHz)

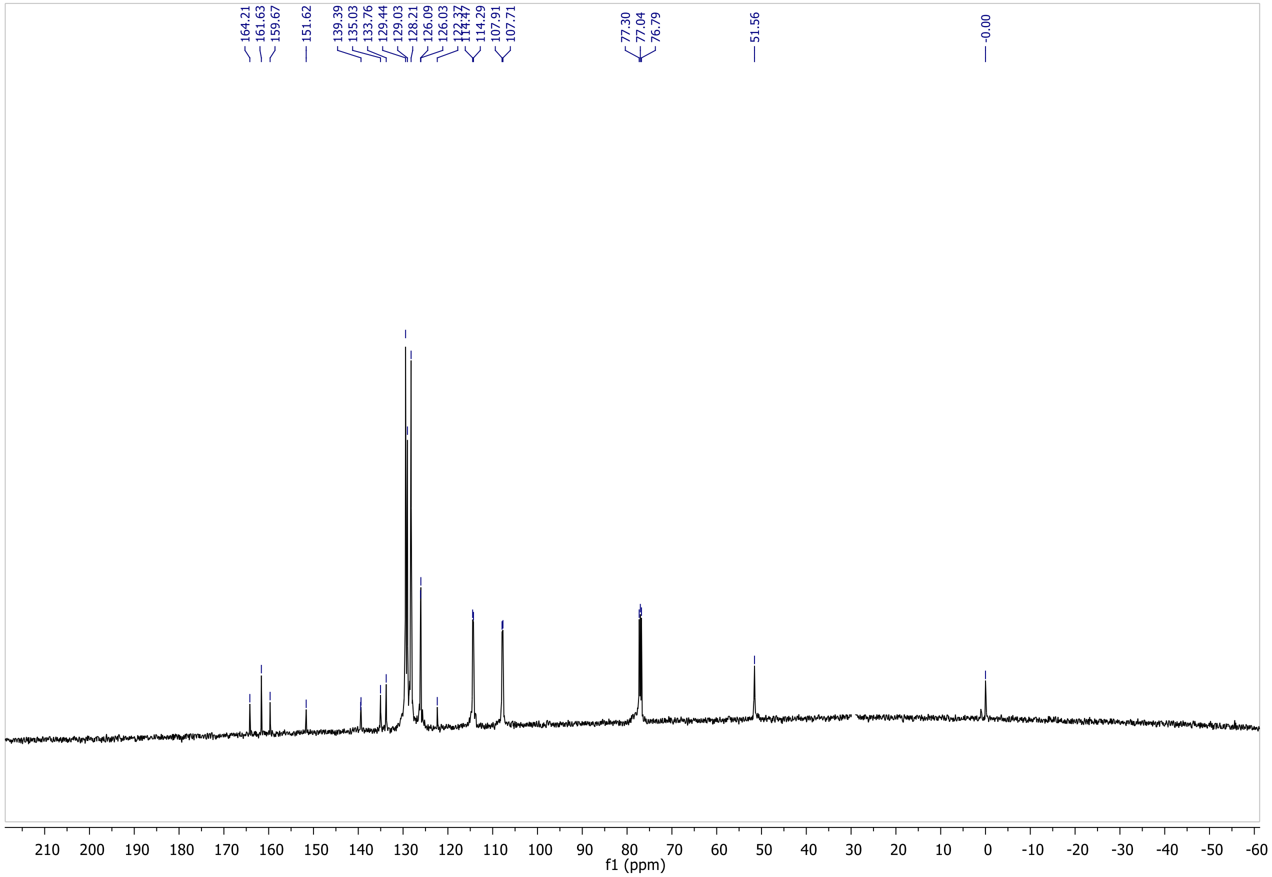

References

1. Liu, J.; Chen, G.; Xing, J.; Liao, J. *tert*-Butanesulfinylthioether ligands: synthesis and application in palladium-catalyzed asymmetric allylic alkylation. *Tetrahedron: Asymm.* **2011**, *22*, 575-579.

2. Peng, X.; Ma, C.; Tung, C.-H.; Xu, Z. Cu-catalyzed three-component coupling of aryne, alkyne, and benzenesulfonothioate: modular synthesis of *o*-alkynyl arylsulfides. *Org. Lett.* **2016**, *18*, 4154–4157.

3. Li, M.; Wang, T.; An, Z.; Yan, R. $B(C_6F_5)_3$-Catalyzed cyclization of alkynes: direct synthesis of 3-silyl heterocyclic compounds. *Chem. Commun.* **2020**, *56*, 11953-11956.

4. Warner, A. J.; Churn, A.; McGough, J. S.; Ingleson, M. J. BCl_3-induced annulative oxo- and thioboration for the formation of C3-borylated benzofurans and benzothiophenes. *Angew. Chem. Int. Ed.* **2017**, *56*, 354-358.

5. Li, Y.; Gryn'ova, F.; Saenz, F.; Jeanbourquin, X.; Sivula, K.; Corminboeuf, C.; Waser, J. Heterotetracenes: flexible synthesis and in silico assessment of the hole-transport properties. *Chem. Eur. J.* **2017**, *23*, 8058-8065.

6. An, S.; Zhang, Z.; Li, P. Metal-free synthesis of selenodihydronaphthalenes by selenoxide-mediated electrophilic cyclization of alkynes. *Eur. J. Org. Chem.* **2021**, *2021*, 3059-3070.

7. Wen, S.-M.; Lin, C.-H-; Chen, C.-C.; Wu, M.-J. Efficient synthesis of 3-benzoyl benzo[*b*]thiophenes and raloxifene via mercury(II)-catalyzed cyclization of 2-alkynylphenyl alkyl sulfoxides. *Tetrahedron* **2018**, *74*, 2493-2499.

8. Li, M.; Wang, T.; An, Z.; Yan, R. $B(C_6F_5)_3$-Catalyzed cyclization of alkynes: direct synthesis of 3-silyl heterocyclic compounds. *Chem. Commun.* **2020,** *56*, 11953-11956.
